# Ureas are identified as the first neutral O-donors broadly effective in stabilizing ionic liquids and other salts of boron-centred cations: synthesis and detailed characterization

**DOI:** 10.1039/d5ra05311k

**Published:** 2025-10-27

**Authors:** Margaret E. Crowley, Gabriel A. Merchant, James H. Davis, Christopher D. Stachurski, Matthias Zeller, E. A. Salter, A. Wierzbicki, Paul C. Trulove, David P. Durkin, Grace L. Kingrey, Edgar E. Escalante, Richard A. O'Brien, Novita M. Whillock

**Affiliations:** a Department of Chemistry, University of South Alabama Mobile AL 36688 USA jdavis@southalabama.edu; b Department of Chemistry, United States Naval Academy Annapolis MD 21402 USA; c Department of Chemistry, Purdue University West Lafayette IN 47907 USA; d Alabama School of Mathematics and Science Mobile AL 36604 USA

## Abstract

Stimulated by recent work showing them to be promising electrolytes in batteries and supercapacitors, and to have useful levels of antibacterial, antifungal, and antiviral activity, there is growing interest in salts of boron-centered cations – boronium ions. However, compared to the number of existing structural variants among molecular cations such as ammonium ions, that of boroniums is minescule. This likely stems from the narrow scope of Lewis base types – largely amines and *N*-heterocycles – known to stabilize cationic boron centres. We have now determined that ureas, in combination with amines, are effective in stabilizing boronium cations, greatly enhancing the scope of structural-geometric space which can be explored with these compounds. And, depending on the specific urea utilized, ionic liquids based upon the new boroniums are effective H-bond donors despite being ‘aprotic’ ILs in nature. The new materials have been assessed by a broad array of techniques, including TGA, DSC, CV, NMR, and X-ray crystallography.

## Abstract 2

Urea-stabilized boronium ions are good H-bond donors. They also can have multiple, orientationally flexible H-bond donor sites in a single cation. Unlike protic ILs, the boroniums are not subject to ready de-ionization in the presence of bases.

## Graphical abstract

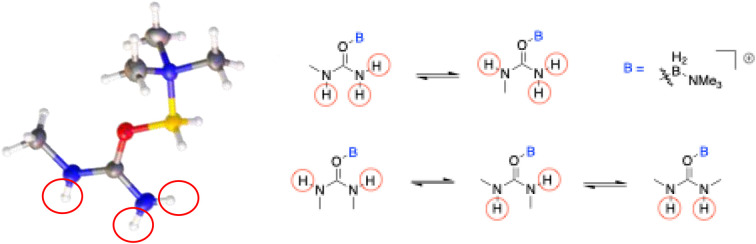

## Introduction

Ionic liquids and other salts derived from boronium (+1) ions are increasingly valued because of their very wide electrochemical windows when used as electrolytes, as well as their recently discovered antibacterial, antifungal, and antiviral properties.^1–12^ They are also potentially valuable building blocks for materials such as porous solids, hybrid organic–inorganic glasses/solids, and even plastic crystals. However, the recent upswing of research activity on boronium (+1) salts has been limited to work on cations that have two N-donor ligands along with two H-atoms bonded to the B-centre – *e.g.*, cations of the form L_2_BH_2_^+^ (L = tertiary amine and/or *N*-heterocycle). These are the archetype of boronium ion structure and they have been almost exclusively the basis for boronium ion studies since the 1960s. This is perhaps not surprising given that they are, in general, highly stable.^1–7^

In sharp contrast, while stable borane (BH_3_) complexes of neutral O-donors (*e.g.*, THF, Et_2_O, *etc.*) are well-known, there have been very few boron cations described which have neutral O-donors as ligands. A handful of examples of the type [Me_3_NBH_2_-X]^+^, wherein X = DMSO, trimethylphosphine oxide, or HMPA (hexamethylphosphoramidate) have been reported, but in comparison to typical L_2_BH_2_^+^ cations they are unstable, especially towards hydrolysis.^13,14^ There is also an inferred example of coordination of diethyl ether to a boronium cation, but the putative cation decomposes rapidly.^15^ Finally, in a 1969 paper by Miller, *et al.*, the formation of two “intriguing,” amide-containing boronium salts, one incorporating dimethylformamide (DMF) and the other dimethylacetamide (DMA) was claimed. The authors made a tentative case for O-coordination to the boron but went on to say that it was “premature” to regard their data as “confirming or negating a structure hypothesis.”^16^ Finally, there are established tetrahedral complexes of other Group 13 cations – specifically Al^+^ and Ga^+^ – that are supported by neutral O-donor ligands. However, these are notoriously water sensitive because their d-orbitals (which boron lacks) render them vulnerable to facile nucleophilic attack by water.^17^

**Fig. 1:**
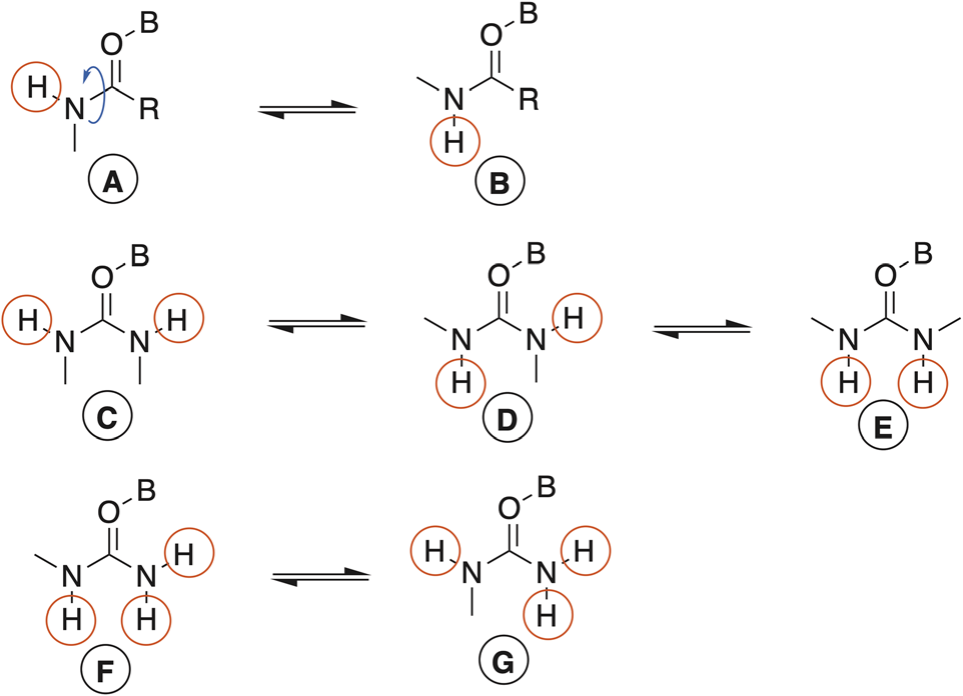
Possible orientations of N–H bonds in ureas coordinated to boron centres depending on the number and nature of the substituents.

Given the paucity of examples of and data on boron cations supported by neutral O-donors, we decided to see if the balance might be tilted towards improved stability by utilizing ureas as ligands, a class of neutral O-donors known to have high degrees of partial negative charge on O. By doing so, we hoped to generate new data of value concerning the chemistry of boron, as well as to broaden the scope of structurally and compositionally unique cations available for use in creating ionic liquids and hybrid organic–inorganic solids. Further, any new cations would be “aprotic” and resistant or perhaps inert to charge-neutralizing deprotonation reactions. At the same time, it was expected that these cations would nevertheless have strong H-bond donation capacity, with multiple sites in cations having more than one ureido N–H. Both of these are factors of significance to ionic liquids and engineered ionic solids.^18–22^ We likewise anticipated that in the case of species bearing two different substituents on the urea nitrogen (*e.g.*, H and alkyl), these cations would have dynamically mutable structures due to the well-known tendency of amide bonds to adopt *cis* or *trans* configurations (Fig. 1). Too, the steric impact on boron of urea, N-heterocycle, and tertiary amine ligands were expected to be very different. In the former, there is a one-atom separation (O) between the greatest bulk of the ligand and the boron. In the N-donors, the ligand – with its steric bulk – is bonded directly to the boron. Consequently, greater degrees of conformational freedom would be available to urea-supported boroniums, a factor known to impact the physical properties of ILs.

**Fig. 2:**
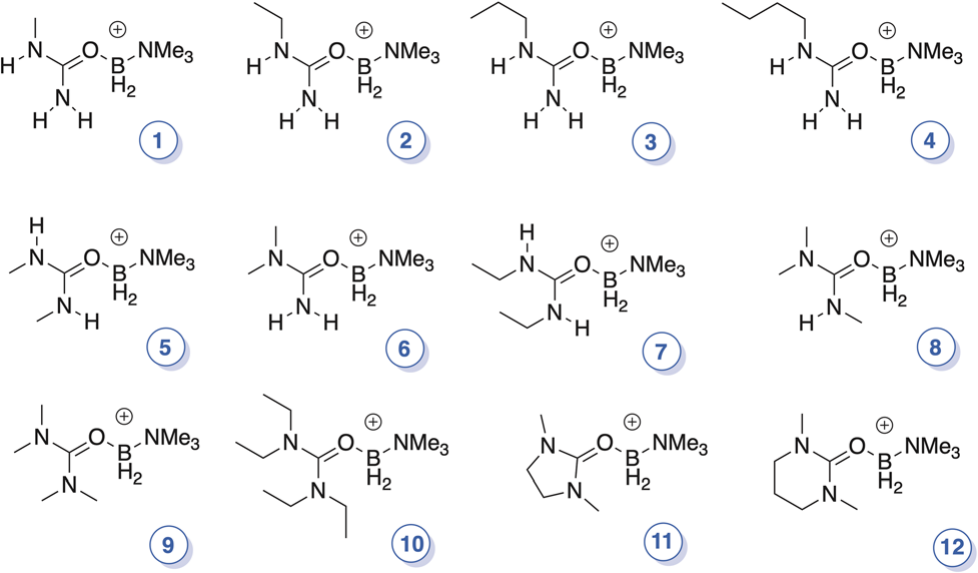
Structures of the new urea-ligated boronium cations; where applicable, *cis*/*trans* orientations are arbitrary.

Herein we report the successful synthesis of iodide salts of a dozen urea-supported boronium (+1) cations (Fig. 2); all are air-, water-, and thermally stable. From the iodides, bis(trifluoromethanesulfonyl)imide (Tf_2_N^−^) salts of the cations were then prepared and characterized by multinuclear NMR, TGA, DSC, CV, and X-ray crystallography††Depending upon the cation, X-ray structures were obtained on Tf_2_N^−^, I^−^, or BPh_4_^−^ salts. As shown in Fig. 2, the ureas include both cyclic and acyclic species, and examples bearing one, two, three, or four *N*-alkyl group(s).

## Experimental

### Representative synthetic procedure – I^−^ and Tf_2_N^−^ salts of 7

**Fig. 3:**
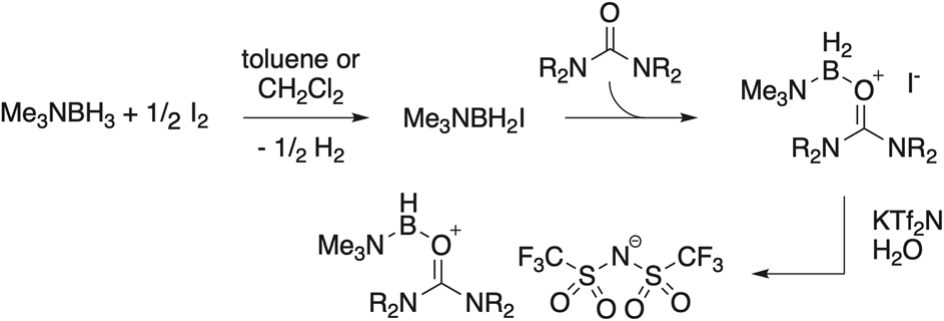
General synthesis of the new urea-ligated boronium salts.

The syntheses of the boronium salts (Fig. 3) followed a procedure introduced by Douglass, refined by Ryschkewitch, and routinely used in our boronium research.^2–4,8,9,23,24^ In a well-ventilated fume hood, a 1 L Erlenmeyer flask was charged with a magnetic stir bar and 300 mL of reagent grade toluene. While stirring, 10.0 g (0.137 mol) Me_3_NBH_3_ was added in one portion. Once dissolved (3–5 min), 17.3 g (0.068 mol) of I_2_ was added in portions, taking care that the rate of addition was such that foaming from H_2_ evolution was not so brisk as to allow the flask contents to overflow (note: we routinely perform these syntheses in air). Initially, the solution is an orange to dark brown color which fades to pale yellow within about 30 min. After 30 min from the time of the last I_2_ addition, 15.9 g (0.137 mol) *N*,*N*′-diethyl urea was added in one portion. Any color remaining in the solution was rapidly discharged. After stirring overnight, the toluene was removed using a rotary evaporator, leaving behind a viscous, opaque yellow liquid. [In the syntheses of many other complexes, the iodide salt was a solid that precipitated directly from the toluene and was isolated by simple filtration followed by washing with ether]. Water (*ca.* 200 mL) was added to the viscous liquid iodide salt, forming a light-yellow solution. To this solution was added, with stirring, 46.0 g (0.144 mol) KTf_2_N. A dense, colorless hydrophobic phase formed rapidly. After stirring overnight, the two phases were separated, and the lower (product) phase dissolved in 300 mL CH_2_Cl_2_ and dried over anhydrous MgSO_4_. After separating the drying agent and rotary evaporation of the CH_2_Cl_2_, the final product, 7 Tf_2_N, was obtained as a near-colorless, slightly viscous liquid (52 g, 0.11 mol, 82% [unoptimized]).

### NMR spectroscopy

High-field NMR spectra were acquired on all Tf_2_N^−^ salts of the new boronium ions to confirm their character. The spectral data are provided in the SI. The spectra – ^1^H, ^13^C, ^10^B, and ^19^F – were collected using a JEOL JNM-ECA series, 500 MHz FT-NMR spectrometer. For the iodide salts of compounds 2 and 7 used in a pH stability study (*vide infra*) the ^1^H spectra are reported in ppm referenced to residual HDO/H_2_O the D_2_O solvent (4.79 ppm). All other NMR analyses were performed in acetone-d_6_ referenced at 2.05 ppm in ^1^H and 206.26 ppm for ^13^C. Note that ^10^B spectra were acquired rather than the more commonly used ^11^B; in our experience with boronium ions, the former tends to produce spectra with narrower line widths and not requiring baseline correction.

### Thermogravimetric analysis

The thermal stability of the Tf_2_N^−^ salts of trications 1–12 were evaluated using thermal gravimetric analysis (TGA; TA Instruments TGA 5500). Samples were loaded into platinum pans and heated under nitrogen from 20–800 °C at a heating rate of 10 °C min^−1^. The decomposition temperature (*T*_5_) was taken as the point at which 5 wt% of the initial sample mass was lost.

### Differential scanning calorimetry

Thermal phase transitions were measured using differential scanning calorimetry (DSC; TA Instruments Q2000). Samples were sealed in hermetic aluminum pans and cycled from room temperature (RT) to −75 °C or below, then to 50 °C or 350 °C at 10 °C min^−1^ under a helium purge at a flow rate of 25 mL min^−1^.

### Electrochemistry

Electrochemical measurements were performed using either a Biologic SP-200 potentiostat or an Electrosyn 2.0. All samples were analysed using a three-electrode setup consisting of either a glassy carbon or a platinum working electrode (EDAQ, surface area = 7.8 × 10^−3^ cm^2^), a platinum mesh counter electrode, and a home-built Ag/Ag^+^ reference electrode made from a 100 mM solution of silver triflate dissolved in [EMI][Tf_2_N] or [BMI][Tf_2_N] contained within a glass tube sealed with a Vycor glass frit.

### Computational

Geometries of cations 1 and 5–12 were optimized in the gas phase using the wB97XD density functional and the cc-pvtz basis set, as implemented in Gaussian16.^25^ Analytic frequencies were computed to ensure that minima had been found. Then, Natural Bond Order (NBO) charges were assigned, and NMR properties were computed using the GIAO method. Natural Resonance Theory (NRT) analysis was used to assess ionic/covalent bond character using the NBO7 software plug-in for Gaussian16. For representative cations, electrostatic surface potential maps (ELSTATs) were generated by Spartan ’24.^26^

Optimizations for selected cations paired with the Tf_2_N^−^ anion and its oxidized radical counterpart were also carried out in the gas phase using Gaussian 16 and the wB97XD/cc-pvtz computational model.^25^ Spin densities were subsequently rendered using Spartan ’24.^26^

### Single crystal X-ray structure determination

Single crystals of the investigated compounds were coated with a trace of Fomblin oil and were transferred to either the goniometer head of a Bruker Quest diffractometer with a fixed chi angle, a Mo Kα wavelength (*λ* = 0.71073 Å) sealed tube fine focus X-ray source, single crystal curved graphite incident beam monochromator, and a Photon II area detector, or onto a Bruker Quest diffractometer with kappa geometry, a Cu Kα wavelength (*λ* = 1.54178 Å) I-μ-S 3.0 microsource X-ray tube, HELIOS multilayer Montel optics for monochromatization, and a Photon III C14 area detector. Both instruments were equipped with an Oxford Cryosystems low temperature device and examination and data collection were performed at 150 K. Data were collected, reflections were indexed and processed, and the files scaled and corrected for absorption using APEX5 and SADABS.^27,28^ The space groups were assigned using XPREP within the SHELXTL suite of programs and solved by dual methods using ShelXT and refined by full matrix least squares against F^2^ with all reflections using Shelxl2019 using the graphical interface Shelxle.^27–33^ H atoms attached to carbon and boron atoms were positioned geometrically and constrained to ride on their parent atoms. B–H bond lengths were allowed to refine unless stated otherwise. C–H bond distances were constrained to 0.95 Å for aromatic and alkene C–H and CH_2_, and to 1.00, 0.99 and 0.98 Å for aliphatic C–H, CH_2_ and CH_3_ moieties, respectively. Methyl CH_3_ were allowed to rotate but not to tip to best fit the experimental electron density. *U*_iso_(H) values were set to a multiple of *U*_eq_(C) with 1.5 for CH_3_, and 1.2 for C–H, CH_2_, B–H, N–H and NH_2_ units, respectively.

Additional data collection and refinement details, including description of disorder (where present) can be found in the SI. Complete crystallographic data, in CIF format, have been deposited with the Cambridge Crystallographic Data Centre. CCDC 2472950–2472961 contain the supplementary crystallographic data for this paper. These data can be obtained free of charge from The Cambridge Crystallographic Data Centre *via*www.ccdc.cam.ac.uk/data_request/cif.

## Results and discussion

In the ^13^C NMR, the C=O shifts in the new cations cluster in the 159–165 ppm range. In most cases, the C=O chemical shift of a given cation is only slightly impacted by coordination when compared to its shift in the native urea; however, in 5 and 6 the observed change is an upfield shift. Such changes to carbonyl ^13^C resonances tend to occur when nearby electrons are in conjugation with the C=O, or when there is appreciable H-bonding of the group to a nearby H-bond donor. In addition, there are correlations that are seen between structure and the ^10^B NMR values of cations 1–8, all of which incorporate a urea having one or more N-bonded H atoms. In comparison to those of the cations incorporating tetra(*N*-alkyl)ureas, the ^10^B resonances of the species retaining an N–H are comparatively shielded; whereas values for the former are generally around −0.5 ppm, a typical value in an N–H containing cation is around −2.0 ppm.

In the ^1^H spectra, the chemical shift of the NMe_3_ group varies only slightly, generally falling between 2.59 and 2.72 ppm. Likewise, the ^13^C resonance for the methyl groups of this ligand fall within a narrow range, 49.5 ppm ±0.5 ppm. Both the ^1^H and ^13^C resonances for the remaining organic structural elements parallel those in the native free ureas, although differences in chemical shifts are apparent (SI); in almost all cases, the resonances from the coordinated ureas are found downfield from those of their counterparts in the free species. Finally, the ^19^F spectra of the Tf_2_N^−^ salts are in keeping with those of this anion when paired with various cations; the singlet resonances are observed at 80 ± 1 ppm in each compound.^34^

Due to the partial double bond character known to exist between urea nitrogen and carbonyl carbon atoms, *cis–trans* isomerism (Fig. 1) was anticipated and was experimentally observed. For example, the ^1^H- and ^13^C-NMR spectra of neat 7 show that the N-1 and N-3 ethyl groups of the urea ligand are non-equivalent, and likewise the hydrogens on the two nitrogen atoms. This is consistent with a “transoid” orientation of the two as depicted in Fig. 1 (orientation D). The experimental ^1^H spectrum and that computed by the GIAO method were good matches, leading us to assign the N–H oriented towards the boronium centre as that generating the signal at 6.46 ppm, while the other N–H appears at 6.06.

**Fig. 4:**
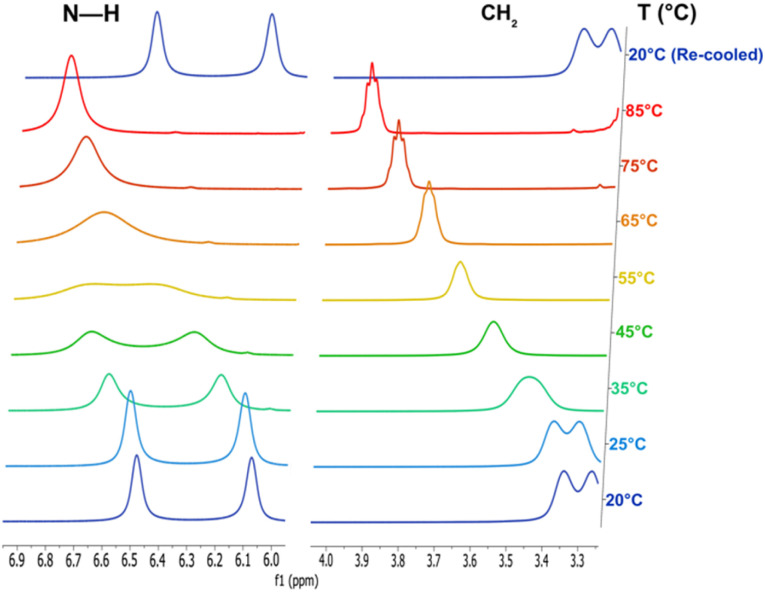
Stack plot of the NMR spectra from the VT experiments on compound 7. As T increases, the coalescence of the N–H and CH_2_ peaks of the urea ligand is clear.

Since *cis–trans* isomerization can be thermally induced in systems with amide bonds, we selected IL 7 to examine the possibility when a urea is coordinated to the cationic boron centre. Consequently, we acquired ^1^H spectra of the neat salt at 10 °C intervals between 25 °C and 85 °C. As evident from a stack plot (Fig. 4 and Table S1) of the N–H region of the ^1^H spectrum, signals for these hydrogens coalesce, consistent with thermally induced rotation about the N–C_carbonyl_ bond. Simultaneous coalescence of the resonances from the ethyl CH_2_ groups was also observed. However, the urea methyl groups are not sufficiently well resolved for the *cis*- and *trans*- configurations to be distinguished. As a result, little T-dependent change was noted with their signals. Significantly, the sample temperature could be serially cycled without any evidence of decomposition, consistent with good thermal stability.

**Fig. 5:**
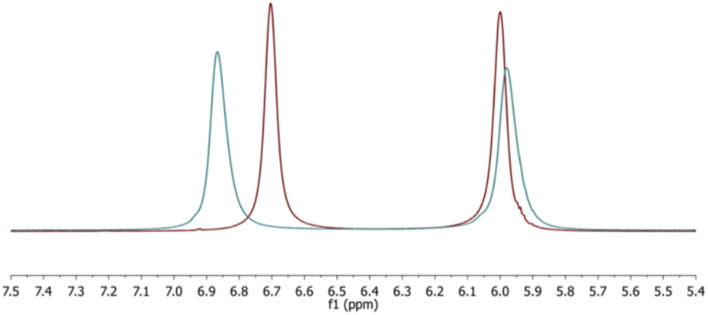
Chloride-induced change in chemical shift of the N–H resonances in 7. IL without chloride in red; post-chloride addition in blue. Spectra acquired at 30 °C.

Ionic liquids incorporating urea groups in cation side chains have been known since 2001, and ureas are common components of deep eutectic solvents (DESs).^19–22^ In the latter case, the capacity of the urea to function as a bidentate N–H hydrogen bond donor to a small, hard anion such as chloride is key to the formation of the low-melting eutectic. Given this background, we were interested in probing the capacity of the coordinated urea N–H moieties to engage in hydrogen bonding in the neat liquid. To do so, a sample of 7 was allowed to stand overnight over an excess of solid Me_4_NCl, our expectation being that the latter would dissolve in the former to the point of saturation (whatever that might be), and by doing so introduce strongly H-bond accepting chloride ions into the IL (Fig. 5). The effort was successful, and an apparent saturation ratio of ten boronium cations per chloride was reached (based upon comparative integration of the Me_4_N^+^ to boronium NMe_3_ signal intensities in the ^1^H–NMR).

The impact of the dissolved chloride on the ^1^H-spectrum was clear (Fig. 5). The N–H signal at *ca.* 6.7 ppm moved downfield by ∼0.2 ppm, while that at *ca.* 6.0 was almost unchanged. This suggests that the chloride anion is in proximity to the locus of positive charge (the O–B–N atom triad), since the most perturbed N–H resonance was previously identified as the one oriented towards that portion of the cation (*vide supra*).

**Fig. 6:**
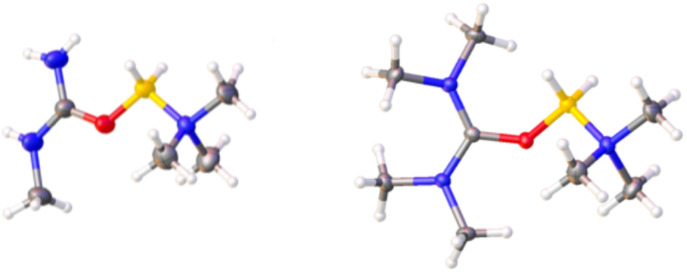
ORTEP of the cations in salts 1 and 9. Tf_2_N^−^ ions are omitted for sake of clarity. Full structure data provided in the SI. Grey = C; white = H; red = O; yellow = B; blue = N.

Single-crystal X-ray structures of salts of 1–12 (with various anions) were obtained (Fig. 6 and SI). In these, bond distances and angles between the boron and the N and O atoms of its ligands were generally unremarkable for four-coordinate boron. However, due to the lone electron pairs on the urea N-atoms being in conjugation with the carbonyl group, the C–N distances (1.23–1.36 Å) are shorter than those in ordinary organic amines (1.47–1.49 Å), and those between the C and O are longer (1.28–1.31 Å) than in typical carbonyl groups (1.20–1.23 Å). Bonds between the B and the N of the Me_3_N ligands in the new compounds range from 1.58 Å to 1.60 Å. These are in the same range as those between Me_3_N and boron in adducts with the highly Lewis acidic boron trihalides, BCl_3_ (1.61), BBr_3_ (1.60), and BI_3_ (1.58), as well as in previously characterized [Me_3_N–BH_2_–L]^+^ salts.^35^

Just as the hydrogen bonding character of ureas has been exploited to create DESs (*vide supra*), it has also been harnessed to bind and activate substrates for organocatalytic purposes. In several instances, the H-bond donor strength of the urea has been increased by incorporating an electron-withdrawing (*e.g.*, nitro) group into the molecular structure, or coordinating the ureido oxygen to a Lewis acid, including boron. In some specific cases, X-ray structures of molecules with intramolecularly B-coordinated ureas have been obtained, providing interesting data against which that of the present urea-boronium salts can be compared.^36–38^

**Fig. 7:**
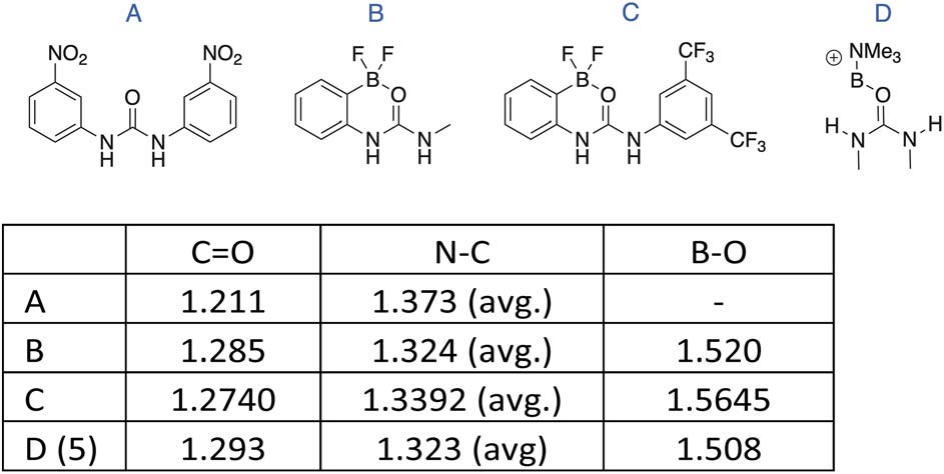
Select bond distances (Å) in compounds containing bonds between a urea oxygen and a boron centre.

In Fig. 7, the structures of four ureas are shown along with select, crystallographically determined bond distances. In urea A, there is no activation by O-coordination to an ancillary Lewis acid site, but this urea is activated compared to its counterpart without the strongly electron-withdrawing nitro groups. *Versus* the baseline values from this molecule, the C=O distances in B and C are longer by 0.07 Å (avg.), while their N–C distances are shorter by 0.04 Å (avg.). This strongly indicates that the boron is inductively pulling electron density from the O–C–N moiety, and by extension, the N-bonded H atoms. The differences are even more pronounced between A and D, the boronium cation of 5. Here, the C=O bond length in the boronium is longer by 0.08 Å, and the N–C distance shorter by 0.05 Å. Finally, the B–O bond distance of D (5) is 0.03 Å shorter than the average of those in B and C. Collectively, these data point to the boron in the cation being more Lewis acidic than those in B and C, although this outcome may also be influenced by the formation of a six-membered ring in the latter compounds. Overall, these data point to potential utility (not yet explored) on the part of the present boronium ions to function as organocatalysts.

**Fig. 8:**
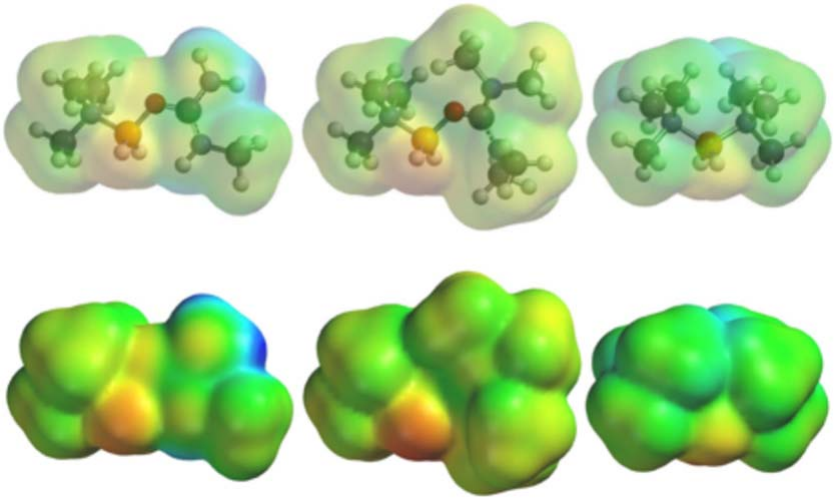
ELSTATs of (l–r) cations 1, 9 and the ‘conventional’ boronium cation [(Me_3_N)_2_BH_2_]^+^. Colors are on a common scale of +233.3 (red) to +582.2 (blue) [in kJ mol^−1^]. The respective minima and maxima are +268.4 and +582.2, +233.3 and +417.7, and +267.5 and +508.9 kJ mol^−1^. The most positive areas on any of the cations are from the N–H areas of 1, while the least positive is on the B–H area of 9. Note that the smallest *δ* between the greatest and least positively charged points on any of these is that of [(Me_3_N)_2_BH_2_]^+^, 241.4 kJ mol^−1^. The dipole moments are 2.36D (1), 1.36D (9), and 1.73D ([(Me_3_N)_2_BH_2_]^+^). Full ELSTAT views of each cation from several perspectives are shown in the SI.

Quantum calculations and follow-up analyses shed further light on the character of the present boronium ions. Electrostatic surface potentials (ELSTATs) (Fig. 8) were computed for cations 1 and 9 to compare with that of the ‘conventional’ [(Me_3_N)_2_BH_2_]^+^ cation. The ELSTATs of cations 1 and 9 span a wider range than the latter, and the potential near the NH_2_ group of 1 is highly positive. In all three cations, the least positive region is found near the BH_2_ moiety.

Using cation 7 as an exemplar, we carried out NBO analysis to assess the nature of bonding in the series. It was found that there was greater double bond character to its ureic C–N bonds (bond orders = 1.33 and 1.30) than to that of free urea (1.06), with a concomitant loss of double bond character of the C–O bond (1.26 *vs.* 1.81). Also, the B–O bond of cation 7 is assigned high ionic character (64.7%). Thus, the ligation can be viewed as chiefly the result of the interaction between BH_2_^+^ and the oxygen of the ureic zwitterion. The B–N bond, also highly ionic (57.3%), can be viewed primarily as the result of cation–dipole interaction between BH_2_^+^ and the neutral amine. The other urea-boronium cations share these attributes, although in the cations that lack an N-bonded H atom (9–12), the B–O bond tends to be more ionic, as in cation 11 at 70.6%. Computations and X-ray structures both show that in 1–8, the N-bonded H atom is preferentially positioned near the boron, directly opposite the NMe_3_ ligand. NBO perturbation analysis suggests that this geometry permits electronic density donation from the σ(N–H) bonding orbital into the σ*(O–B) and σ*(B–N) antibonding orbitals, which are heavily weighted to boron. This may at least in part explain why the assigned NBO charges on boron in 1–8 (*e.g.* +0.286 in 7) fall below the range found for cations 9–12 (+0.307 to +0.326), a result consistent with greater shielding observed in ^10^B NMR in 1–8.

The thermal stabilities of the Tf_2_N^−^ salts of the new cations were determined using thermal gravimetric analysis (TGA), each salt being heated at a rate of 10 °C min^−1^ from ambient *T* to 800 °C (Table 1).

**Table 1:** Collected thermal properties. *T*_c_ = temperature of crystallization; *T*_g_ = glass transition temperature; *T*_m_ = melting temperature; *T*_s–s_ = solid–solid transition temperature; *T*_5_ = temperature at which 5% of the sample mass is lost

Compound	*T*_g_ (°C)	*T*_c_ (°C)	*T*_m_ (°C)	*T*_5_ (°C)	*T*_s–s_ (°C)
1	−59.6	—	47.21	228.89	—
2	−61.56	—	37.53	262.51	—
3	−50.63	15.54	59.26	254.29	—
4	−57.97	−2.07	36.78	259.03	—
5	—	—	70.6	191 ± 4	−3.8
6	−67.95	—	—	162.49	—
7	−67.8	—	—	177 ± 3	—
8	—	—	—	179.93	—
9	−76.7	—	—	208 ± 18	—
10	—	−18.0	14.0	244 ± 3	−6.0
11	−76.5	−41.8	21.3	240 ± 2	—
12	−72.6	—	—	247 ± 17	—

**Fig. 9:**
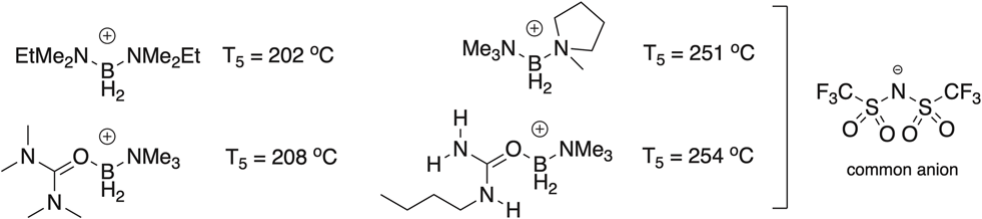
Comparative *T*_5_ values of two ‘conventional’ [L_1_L_2_BH_2_]^+^ type (L_1_, L_2_ = tertiary amine) boronium ions (upper) *versus* (lower) those of 9 and 4. All are as paired with the Tf_2_N^−^ anion.

In general, the thermal stability, as judged by their *T*_5_ values (the temperature by which point 5% mass loss has occurred), is comparable to common boronium Tf_2_N^−^ salts in which both B-supporting ligands are tertiary amines. Eight materials (1, 2, 3, 4, 9, 10, 11, 12) have *T*_5_ values between 207 °C and 263 °C; these compare favourably with typical boroniums supported by two tertiary amine ligands, *e.g.*, the [L_1_L_2_BH_2_]^+^ type (L_1_, L_2_ = tertiary amine). In turn, four salts (5, 6, 7, 8) have sub-200 °C values (162 °C – 191 °C). Overall, the replacement of a tertiary amine ligand in a boronium ion by a urea appears to have at most a modest impact on the thermal stability of the cation (Fig. 9). Parenthetically, we note here that attempts to prepare and isolate boroniums in which both supporting ligands were ureas were unsuccessful.

In addition to thermal decomposition metrics, phase transitions for each of the boronium Tf_2_N^−^ salts were measured using DSC. Each sample was first cooled to −75 °C or below, followed by heating to either 50 °C or 150 °C for room temperature liquids and solids, respectively. Across all samples, consistent behaviour was observed across repeat cycling, which is not always the case with glass-forming boronium cation-containing ionic liquids. In this new suite of ILs, glass transitions were observed in cations with short chain alkyl groups (7, 9) and cyclic moieties (11, 12), and the two examples (3, 6) having two hydrogens retained on a single urea N atom. In certain boronium cations, additional behaviour is observed, both in place of and in addition to glass transitions. Compound 10 undergoes crystallization at −18 °C upon cooling, followed by an apparent solid–solid transition (−6 °C) upon warming, and then it melts at 14 °C on the return cycle – possibly indicating plastic crystalline behaviour by this salt. A similar s–s phase transition is observed with salt 5. Compound 11 exhibits crystallization/melting behaviour (−41.8 °C/21.3 °C) in addition to a prominent glass transition (−76.5 °C). The Tf_2_N^−^ salt of cation 5, which exists as a solid at room temperature, undergoes melting at 70.6 °C, suggesting that higher symmetry and fewer degrees of freedom can lead to elevated melting points, as is generally observed in ionic liquids.

Having recently studied the pH-dependent aqueous stability of boronium (3+) salts we thought it useful to extend our investigation to the present family.^39^ Consequently, the iodide salt of 10 was used as the initial platform for doing so by dissolving it in D_2_O at pHs (pDs) varied monotonically from 1–14, the approach used previously. Its stability was then assessed by acquiring multinuclear NMR spectra on each solution immediately upon dissolution (*t* = 0) and after one week.

Remarkably, the salt proved to be resistant to hydrolysis between pH 1–12 over the length of the study. Specifically, between pH 1–12, no change was observed in the ^1^H, ^13^C, or ^10^B spectra. Emphasising this point, we note that the ^10^B shifts at both *t* = 0 (initial) and *t* = 1 week, across the specified pH values, averaged −0.56 ± 0.04 and −0.58 ± 0.04, ppm. However, pH 12 appears to be the upper limit of its stability; At pH 13, even at *t* = 0, a second boron peak is apparent, and at pH 14 and *t* = 0 these two peaks were replaced by a single very sharp peak at −1.05 ppm, consistent with boron in a tetrahedrally symmetrical environment, possibly [B(OH)_4_]^−^.^40,41^

**Fig. 10:**
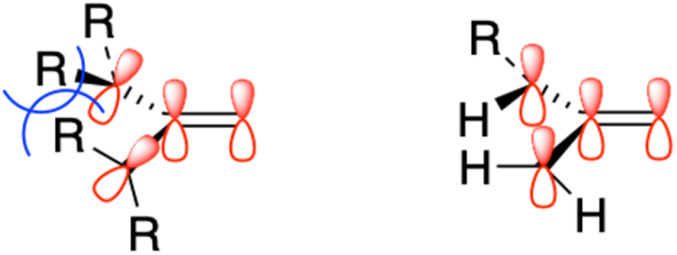
Steric repulsion between *N*-ethyl groups in 2 are posited to diminish the degree of nitrogen lone-pair conjugation with the urea carbonyl group. In contrast, the –NH_2_ and –NHEt nitrogens in 10 can achieve co-planarity with the carbonyl, maximizing conjugation.

We replicated this study using the iodide salt of cation 2, a counterpart in which three of the four ethyl groups of the former are replaced by H. In this instance, the salt was fully stable through pH 13 – an order of magnitude more basic than the maximum pH at which 10 was stable. We posit that this may be due to the ability of the –NH_2_ group in 2 to remain fully coplanar and in conjugation with the urea carbonyl; molecular modelling and the X-ray structure of 10 suggest that based on steric considerations the –NEt_2_ groups are constrained to be somewhat canted with respect to its carbonyl (Fig. 10), thus limiting its conjugation as a result. The NMR spectra from these pH experiments are provided as SI.

We note that the signals from the N–H protons of 2 gradually disappear beginning at *ca.* pH 9, presumably from a base-catalysed exchange with D_2_O. Supporting this interpretation, the solution was subsequently acidified with HCl in water. On doing so, the urea N–H protons were observed once again, at their original chemical shift values and with appropriate integrated intensities. It also bears mention that at pH 14, after one week, a sharp peak at −1.50 ppm is clearly growing in alongside that from the boron of the apparently still intact cation 2. In sum, we reason that both urea boronium cations are decomposed or decomposing after one week at pH 14, but that the rate of decomposition of 2 is slower than that of 10.

As mentioned, there is growing interest in utilizing ionic liquids as electrolytes in energy storage devices because of their favourable electrochemical stability and overall safety compared to organic and aqueous systems. Boronium-based ionic liquids are proving to be especially promising in this regard.^3–9^ Consequently, we investigated the electrochemical behaviour of the present boronium salts which are liquids, or which tend to exist as long-lived supercooled phases at ambient T. These were evaluated by cyclic voltammetry (CV) as neat electrolytes and cycled until apparent breakdown of the salt was observed. Each IL exhibited moderate electrochemical stability, and all have operational windows ranging from 4.0 V to 4.7 V, with the most negative cathodic limiting potential seen being that of salt 7 (Table 2). Significantly, these windows are narrower than those previously reported for boronium IL electrolytes (as great as 6.3 V) featuring two N-ligand donors on the BH_2_ center.^4^ However, perhaps the most significant observation associated with the electrochemical experiments has to do with the unexpected oxidation limiting potentials of the Tf_2_N^−^ anion. In the present compounds, these drop below 1 V in some instances (11 and 12), and in almost all cases are *ca.* 1 V lower than is observed for this anion in association with [L_1_L_2_BH_2_]^+^ (L_1_, L_2_ = tertiary amine) type boroniums. This suggests a profound cation impact on the oxidative stability of this anion, which is commonly valued for its resistance to oxidation.^2–9^ In light of these unexpected observations, further calculations were performed in the hope of gaining insight into the phenomenon.

**Table 2:** *E*_ox_ and *E*_red_ of the neat salts which were intrinsic liquids at ambient *T* were measured as the potentials where the current density reached 1 mA cm^−2^ at a glassy carbon electrode using CV (at 45 °C); Δ*E* is reported as the difference between *E*_ox_ and *E*_red_

Compound	*E*_ox_ (V)	*E*_red_ (V)	Δ*E* (V)
6	1.86	−2.75	4.61
7	1.38	−3.39	4.77
8	1.69	−2.66	4.35
9	1.13	−3.21	4.32
10	1.17	−3.09	4.26
11	0.96	−3.13	4.09
12	0.93	−3.15	4.08

**Fig. 11:**
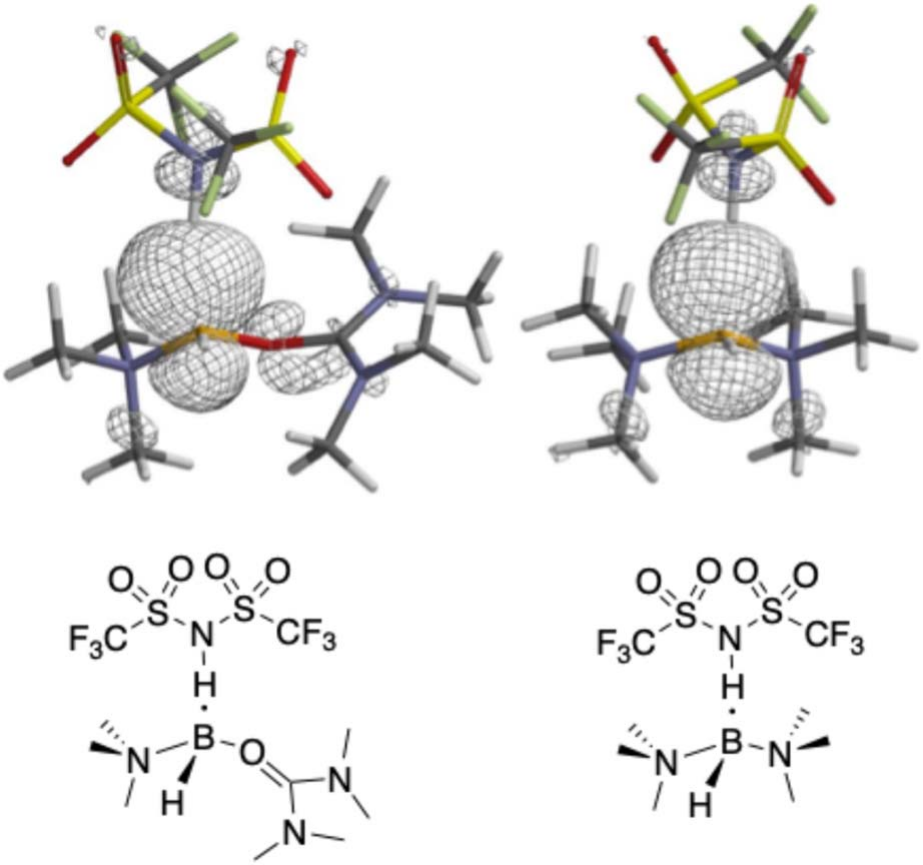
Spin density plots of HTf_2_N with cations 9 (left) and the bis(trimethylamine)-based boronium cation (right) after oxidation. Note the participation of the urea oxygen, carbonyl carbon, and nitrogen atoms in delocalizing the spin density of the unpaired electron in the urea-supported cation.

In principle, oxidation of an isolated Tf_2_N^−^ anion leads to a radical with an unpaired electron localized on the central nitrogen atom. However, our calculations indicate that when a Tf_2_N^−^ anion is proximal to one of the urea-supported boronium cations, oxidation occurs with the concerted transfer of a B-bonded hydrogen atom from the boronium to the Tf_2_N^−^ nitrogen. The products of the oxidation are thus the parent acid (HTf_2_N) in complex with a radical boronium cation. In this complex, better delocalization of the unpaired electron on the boron atom in the urea-supported boroniums appears to account for the lower measured oxidation potentials of the Tf_2_N^−^ anion. For example, the NBO spin charge (Table S2) on B in the cation 9 complex is just +0.765 *versus* +0.865 in the L_1_ = L_2_ = trimethylamine boronium complex, indicating greater delocalization in the former. The spin densities of these two complexes (Fig. 11) show that the primary lobe of the former on the boron is comparatively smaller and that spin density is shared by its ureic oxygen (spin charge = +0.023). Delocalization onto the nitrogen of HTf_2_N occurs in both complexes, but to a greater degree in the cation 9 complex. Further, we suggest that our proposal that oxidation of Tf_2_N^−^ generates a radical boronium cation by H atom transfer is reasonable given the known capacity of boroniums to undergo free-radical replacement of B–H by halogens.^1^

## Conclusions

A library of salts incorporating boronium cations of the type [L_2_BH_2_]^+^ (L = Lewis base), in which one L is a mono-, di-, tri- or tetra-*N*-alkyl urea, has been prepared and thoroughly characterized (multinuclear NMR, TGA, DSC, CV, sc-X-ray diffraction). Insofar as we are aware, these are the first urea-containing boronium ions to be prepared, their closest counterparts being two incompletely characterized amide containing species.^16^ It must be stressed that there is profound distinction between amides and ureas as ligands, since Inada, *et al.*,^41^ showed that ureas are profoundly stronger (better) ligands to Lewis acid centres than are amides. The impact of this is clearly seen in the high degree of water, air, and thermal stability manifested by the urea boroniums, these attributes being quite similar to those observed with ‘classical’ boronium salts in which both ligands of the [L_2_BH_2_]^+^ cation are amines.

Those members of the present cation family which retain one or more hydrogen atoms on a urea nitrogen are good H-bond donors, and in that way resemble “protic” ionic liquids, PILs. However, to paraphrase Swadźba-Kwaśny, the physico-chemical properties of the latter are dictated by the presence of labile protons, an attribute which can limit their use to low pH conditions or conditions in which a stronger Lewis base is present than that from which the PIL is made.^42^ In marked contrast, exemplars from the new urea boroniums are stable from pH = 1 to pH = 13 and appear to be immune to such limitations. This has profound implications for the possibility of further development of urea boronium ILs as solvents in, for example, the extraction of species from biomass which are rich in H-bond acceptor centres such as alkaloids.

Finally, it bears mention that while the electrochemical window of the new boroniums is narrower than that observed for [L_2_BH_2_]^+^ in which both ligands are amines, the coordinated ureas can have an unexpected impact on the oxidation potential of the highly oxidation-resistant Tf_2_N^−^ anion. Consequently, follow-on work to look for similar impacts on other anions associated with urea boronium cations are clearly merited.

## Author contributions

The project was conceived by J. H. D. Synthetic work was done by M. E. C., G. A. M., R. A. O., N. M. W., and J. H. D. N. M. R. studies were carried out by M. E. C. and G. A. M. Electrochemical and thermal studies were conducted, and the results analysed by, C. D. S., P. C. T., D. P. D., and M. E. C. X-ray crystal structures were acquired by M. Z. Computations were carried out by E. A. S. and A. W. Funding was acquired by J. H. D., P. C. T., and D. P. D. All authors reviewed and corrected the manuscript as needed.

## Conflicts of interest

There are no conflicts to declare.

## Supplementary Material

RA-015-D5RA05311K-s001

RA-015-D5RA05311K-s002

## Data Availability

The data supporting this article have been included as part of the supplementary information (SI). CCDC 2472950–2472961 contain the supplementary crystallographic data for this paper.^43*a–l*^ Supplementary information is available. See DOI: https://doi.org/10.1039/d5ra05311k.
