# Supplementary material for: Ureas are identified as the first neutral O-donors broadly effective in stabilizing ionic liquids and other salts of boron-centred cations: synthesis and detailed characterization
Source: RSC Adv. 2025 Oct 27;15(48):40519–27. doi: 10.1039/d5ra05311k (PMC12557308; doi:10.1039/d5ra05311k)

Supplementary Information (SI) for RSC Advances.
This journal is © The Royal Society of Chemistry 2025

Ureas are identified as the first neutral O-donors broadly effective in stabilizing salts ionic liquids and other salts of boron-centred cations: Synthesis and detailed characterization

Supporting Information

James H. Davis, Jr.,*[a] Christopher D. Stachurski,*[b] Matthias Zeller,[c] Margaret E. Crowley,[a] Gabriel A. Merchant,[a] E. A. Salter,[a] A. Wierzbicki,[a] Paul C. Trulove,[b] David P. Durkin,[b] Grace L. Kingrey,[a] Edgar E. Escalante,[a] R. A. O'Brien,[a] and Novita M. Whillock[d]

a Department of Chemistry, University of South Alabama
b Department of Chemistry, United States Naval Academy
c Department of Chemistry, Purdue University
d Alabama School of Math and Science

Contents

Compiled room-temperature NMR data

NMR chemical shift, coordinated and free O-donor ligands

Table S1

Table S2

Cyclic voltammograms

Calorimetric scans (DSC))

Thermogravimetric analyses (TGA)

pH-dependent aqueous stability NMR data

Crystallographic data

Raw NMR spectra

Compiled NMR data

NMR : ^{1}H, ^{13}C, ^{10}B*, ^{19}F

All spectra were acquired on a JEOL JNM-ECA 500 in 5 mm tubes (neat samples in 5 mm coaxial tubes with deuterated lock/reference solvent in the inner tube). All deuterated solvents purchased from MilliporeSigma, Cambridge Isotope Laboratories, or Oakwood Chemical and used as received.

*In our experience, ^{10}B NMR spectra of boronium ions have narrower line widths and smoother baselines than do those produced by ^{11}B NMR. No baseline corrections were applied.

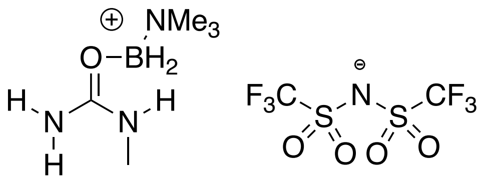

Compound 1. ^{1}H (acetone-d_6) ppm: 2.59 and 2.66, singlets (amide rotational isomers), collectively 9H, NMe_3; 2.86 and 2.91, d, collectively 3H, NMe; 6.77, br s, 1H, NH; 7.03 and 7.12, overlapping d, collectively 2H, NH_2. ^{13}C (acetone-d6) ppm: 28.52 and 29.65, NCH_3 (amide rotational isomers); 49.34 and 49.60 (amide rotational isomers), NMe_3; 120.50, q, CF_3; 161.0 and 161.4, (amide isomers), CO. ^{10}B (acetone-d_6) ppm: -2.20, B; ^{19}F (acetonitrile-d_3) ppm: -79.8, F.

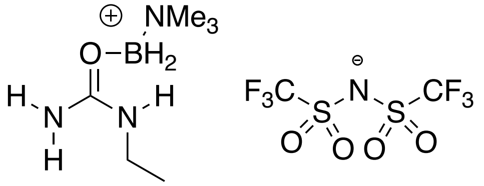

Compound 2. ^{1}H (acetone-d_6) ppm: 1.13 and 1.18, t, (amide rotational isomers), collectively 3H, $CH_2\mathit{CH_3}$; 2.59 and 2.79, s, (amide rotational isomers), collectively 9H, NMe_3; 3.33, overlapping q, $\mathit{CH_2}CH_3$; 6.68, br s, 1H, NH; 7.04, br, 2H, NH_2. ^{13}C (acetone-d_6) ppm: 13.75 and 14.60, (amide isomers), $CH_2\mathit{CH_3}$; 36,33 and 37.37, NCH_3 (amide rotational isomers), $\mathit{CH_2}CH_3$; 49.32 and 49.62 (amide rotational isomers), NMe_3; 120.51, q, CF_3; 160.3 and 160.4, (amide isomers), CO. ^{10}B (acetone-d_6) ppm: -2.09, B; ^{19}F (acetonitrile-d_3) ppm: -79.6.

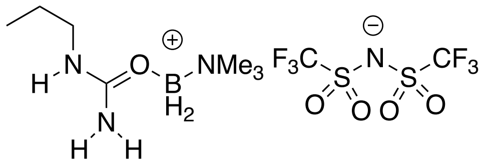

Compound 3. Spectra acquired at 20.1C; at this temperature, peaks are doubled due to cis-trans orientations around the urea N(H, Pr)-C(CO) bond. ^{1}H (acetone-d$_6$) ppm: 0.91, complex m, 3H, CH_3; 1.57, complex m, "2H", CH_2; 1.65, complex m, "2H", CH_2; 2.65, s, "9H", NMe_3; 2.71, s, "9H", NMe_3; 3.31, complex m, 2H, CH_2; [6.93, br, 7.31 br, NH and NH_2]. ^{13}C (acetone-d$_6$) ppm: [11.06 &11.12], CH_3; [23.24 & 23.45], CH_2; [43.08 & 44.19], [49.44 & 49.74], NMe_3; 120.2, q, CF_3; [160.66 & 160.87], CO. ^{10}B (acetone-d$_6$) ppm: -2.05, B; ^{19}F (acetonitrile-d$_3$) ppm: -79.8, F.

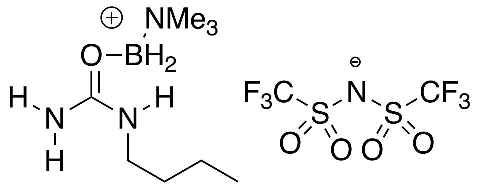

Compound 4. ^{1}H (acetone-d$_6$) ppm: 0.86, t, 3H, N-$CH_2CH_2CH_2\mathit{CH_3}$; 1.33, overlapping m, 2H, N-$CH_2CH_2\mathit{CH_2}CH_3$; 1.50 and 1.56, separated multiplets, 2H, N-$CH_2\mathit{CH_2}CH_2CH_3$ (amide rotational isomers); 2.59 and 2.65, s (amide rotational isomers) collectively 9H, NMe_3; 3.28, overlapping m, 2H, N-$\mathit{CH_2}CH_2CH_2CH_3$; 6.69, br s, 1H, NH; 7.03 and 7.08, br s, 2H, NH_2. ^{13}C (acetone-d6) ppm: 13.75 and 14.60, (amide isomers), $CH_2\mathit{CH_3}$; 36,33 and 37.37, NCH_3 (amide rotational isomers), $\mathit{CH_2}CH_3$; 49.32 and 49.62 (amide rotational isomers), NMe_3; 120.45, q, CF_3; 160.4 and 160.6, (amide isomers), CO. ^{10}B (acetone-d$_6$) ppm: -2.15, B; ^{19}F (acetonitrile-d$_3$) ppm: -79.8, F.

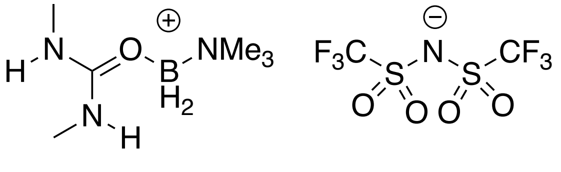

Compound 5. ^{1}H (acetone-d$_6$) ppm: 2.66, s, 9H, NMe_3; 2.88, d, 3H, NMe; 2.91, d, 3H, NMe; 6.86, br, 1H, NH; 7.31, br, NH. ^{13}C (acetone-d$_6$) ppm: 27.51, CH_3; 28.62, CH_3; 49.60 NMe_3; 120.6, quartet, CF_3; 159.97, CO. ^{10}B (acetone-d$_6$) ppm: -2.11, B; ^{19}F (acetone-d$_6$) ppm: -79.8

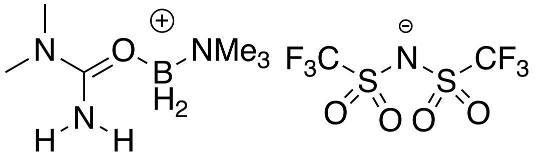

Compound 6. ^{1}H (acetone-d$_6$) ppm: 2.69, s, 9H, NMe_3; 3.12, s, 6H, NMe_2; 7.05, br, 2H, NH_2. ^{13}C (chloroform-d) ppm: 37.09, NMe; 37.40, NMe; 49.67, NMe_3; 120.70, (quartet), CF_3; 159.70, CO. ^{10}B (chloroform-d) ppm: -1.89, B; ^{19}F (chloroform-d) ppm: -79.8

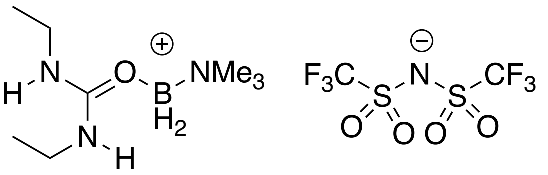

Compound 7. Neat, D_2O external standard. ^{1}H ppm: 1.42, overlapping t, 6H, $NCH_2\mathit{CH_3}$; 2.59, s, 9H, NMe_3; 3.24, br, 2H, $N\mathit{CH_2}CH_3$; 3.32, br, 2H, $N\mathit{CH_2}CH_3$; 6.06, br, 1H, NH; 6.46, br, 1H, NH. ^{13}C ppm: 13.06, 14.13, CH_3; 36.17, 36.43, CH_2; 49.07, NMe_3; 121.2 (quartet), CF_3; 157.79, CO. ^{10}B ppm: s, -0.72; ^{19}F ppm: s, -79.9

Compound 7. (acetone-d_6) ppm: 1.17, t, 6H, $NCH_2\mathit{CH_3}$; 2.66, s, 9H, NMe_3; 3.29, br, 2H, $N\mathit{CH_2}CH_3$; 3.38, br, 2H, $N\mathit{CH_2}CH_3$; 6.61, br, 1H, NH; 7.11, br, 1H, NH. ^{13}C (acetone-d_6) ppm: 13.90, CH_3; 14.91, CH_3; 36.78, CH_2; 37.14, CH_2; 49.61, NMe_3; 121.2 (quartet), CF_3; 164.65, CO. ^{10}B (acetone-d_6) ppm: s, -2.13; ^{19}F (acetone-d_6) ppm: s, -79.8

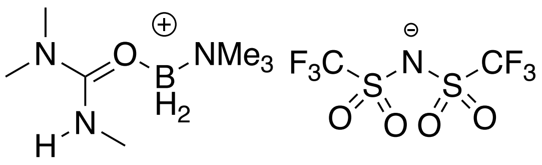

Compound 8. ^{1}H (acetone-d_6) ppm: 2.71, s, 9H, NMe_3; 2.96, d, 3H, NMe; 3.07, s, 6H, NMe_2; 7.10, br, 1H, NH. ^{13}C (acetone-d_6) ppm: 29.77, NMe; 38.33, NMe; 49.54, NMe_3; 121.50, (quartet), CF_3; 148.37, CH; 162.04, CO. ^{10}B (acetone-d6) ppm: -0.68, B; ^{19}F (acetone-d_6) ppm: -79.8 F.

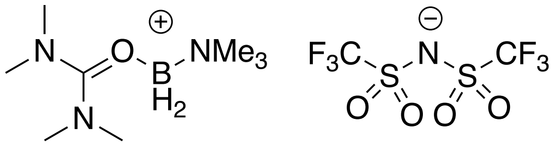

Compound 9. ^{1}H (acetone-d_6) ppm: 2.72, s, 9H, NMe_3; 3.07, s, 12H, NMe_2. ^{13}C (acetone-d_6) ppm: 40.17, NMe_2; 49.72, NMe_3; 121.2 (quartet), CF_3; 164.65, CO. ^{10}B (acetone-d_6) ppm: s, -0.72; ^{19}F (acetone-d_6) ppm: s, -79.8

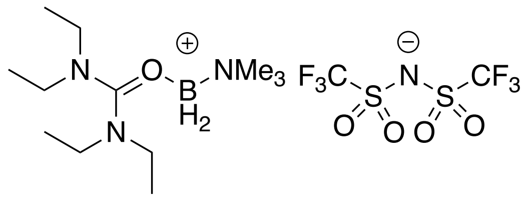

Compound 10. ^{1}H (acetone-d_6) ppm: 1.21, t, 12H, CH_3; 2.72, s, 9H, NMe_3; 3.44, q, 8H, CH_2. ^{13}C (acetone-d_6) ppm: 13.12, CH_3; 44.49, NMe_3; 49.79, CH_2; 121.3 (quartet), CF_3; 164.98, CO. ^{10}B (acetone-d_6) ppm: -0.57, s. ^{19}F (acetone-d_6) ppm: -79.7.

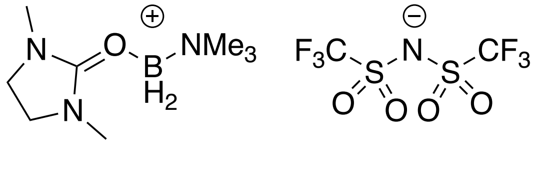

Compound 11. ^{1}H (acetone-d_6) ppm: 2.68, s, 9H, NMe_3; 3.00, s, 6H, NMe; 3.71, s, 4H, CH_s; 3.32; ^{13}C (acetone-d_6) ppm: 32.86, CH_2; 47.26, NMe; 49.40, NMe_3; 121.2 (quartet), CF_3; 162.68, CO. ^{10}B (acetone-d_6) ppm: s, -0.38; ^{19}F (acetone-d_6) ppm: s, -79.9

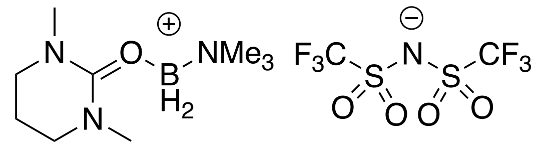

Compound 12. ^{1}H (acetone-d_6) ppm: 2.04, m, 2H, CH_2; 2.71, s, 9H, NMe_3; 3.08, s, 6H, NMe; 3.47, s, 4H, CH_2. ^{13}C (acetone-d_6) ppm: 20.67, CH_2; 37.90, CH_2; 48.79, NMe; 49.55, NMe_3; 121.4 (quartet), CF_3; 159.02, CO. ^{10}B (acetone-d_6) ppm: -0.67, s. ^{19}F (acetone-d_6) ppm: -81.5.

NMR chemical shift differences between coordinated vs. corresponding free urea ligands.

Relevant chemical shifts of the urea ligand, along with the corresponding free (uncomplexed) ligand shifts in parentheses. Note: Of necessity, the NMRs of the free ureas used in compounds 1-3 were acquired in D_2O due to poor solubility in other solvents. Cations paired with Tf_2N^- unless otherwise noted.

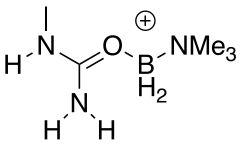

Compound 1. Spectra acquired at 20.1C; at this temperature, peaks for the ligand in the boronium are doubled due to cis-trans orientations around the urea N(H, Me)-C(CO) bond. ^{1}H (acetone-d_6) ppm: **2.87, 2.91** (**2.50**), singlets, NCH_3; **6.77** (**5.45**, br, NH), br, **6.99** (**5.94**, br d, NH).^{13}C (acetone-d_6) ppm: [**28.52** & **29.65** (**26.37**)], NCH_3; [**160.92** & **161.40** (**162.11**)], CO.

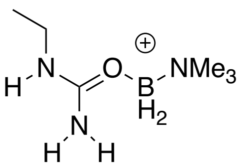

Compound 2. Spectra acquired at 20.1C; at this temperature, peaks for the ligand in the boronium are doubled due to cis-trans orientations around the urea N(H, Et)-C(CO) bond. ^{1}H (acetone-d_6) ppm: **1.13** & **1.18** (**0.92**), triplets, 3H, CH_3; **3.33** (**2.93**), overlapping q, 2H, CH_2; [**6.68** (**5.40**, br, NH), **7.04** (**6.06**, br, NH_2]. ^{13}C (acetone-d_6) ppm: [**13.78** &**14.60** (**14.43**)], CH_3; [**36.33** & **37.37** (**35.03**)], CH_2; [**160.28** & **160.41** (**161.25**)], CO.

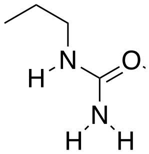

Compound 3. Spectra acquired at 20.1C; at this temperature, peaks for the ligand in the boronium are doubled due to cis-trans orientations around the urea N(H, Pr)-C(CO) bond. ^{1}H (acetone-d_6) ppm: **0.91** (**0.85**), complex m, 3H, CH_3; [**1.57** (**1.43**), complex m, “2H”, CH_2; **1.65**, complex m, “2H”, CH_2]; **3.31** (**3.01**), complex m, 2H, CH_2; [**6.93** (**5.42**, br, NH_2), br, **7.31** (**6.00**, br, NH) NH and NH_2]. ^{13}C (acetone-d_6) ppm: [**11.06** &**11.12** (**11.59**)], CH_3; [**23.24** & **23.45** (**24.18**)], CH_2; [**43.08** & **44.19** (**42.29**)]; [**160.66** & **160.87** (**160.41**)], CO.

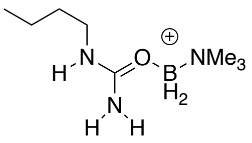

Compound 4. Spectra acquired at 20.1C; at this temperature, peaks for the ligand in the boronium are doubled due to cis-trans orientations around the urea N(H, Bu)-C(CO) bond. ^{1}H (acetone-d$_6$) ppm: **0.86** (**0.86**), complex m, 3H, CH_3; [**1.32**, (**1.30**), complex m, CH_2]; [**1.50** (**1.39**), complex m, "2H", CH_2; **1.58**, complex m, "2H", CH_2]; **3.30** (**3.05**), complex m, 2H, CH_2; [**6.69** (**5.48**, br, NH_2), **7.03** (**6.03**, br, NH). ^{13}C (acetone-d$_6$) ppm: [**13.57** (**14.07**)], CH_3; [**29.80** & **31.79** (**33.2, 40.19**)], CH_2; [**160.40** & **160.55** (**160.53**)], CO.

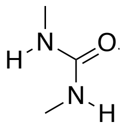

Compound 5. ^{1}H (acetone-d$_6$) ppm: **2.88** (**2.63**), d, 3H, NMe; **2.91** (2.64), d, 3H, NMe; [**6.86**, br, 1H, NH; **7.31**, br, NH] (**5.91 single broad peak**). ^{13}C (acetone-d$_6$) ppm: [**27.51**, CH_3; **28.62**, CH_3;] (**26.82 single peak**); **159.97** (**161.04**), CO.

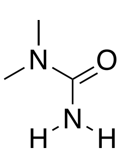

Compound 6. ^{1}H (acetone-d$_6$)* ppm: **3.12** (**2.70**), s, 6H, NMe_2; **7.05** (**4.65**), br, 2H, NH_2. ^{13}C (chloroform-d)* ppm: [**37.09**, NMe; **37.40**, NMe] (**35.99** – single resonance);**159.70** (**160.10**), CO.
*Free ligand spectra acquired in D_2O; insoluble in acetone.

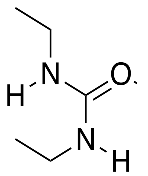

Compound 7. ^{1}H ppm: 1.17 (**1.02**), t, 6H, NCH$_2$*CH$_3$*; 3.29 br, 2H, N*CH$_2$*CH$_3$; 3.38 br, 2H, N*CH$_2$*CH$_3$ (**3.11, q, 4H – single peak for CH$_2$ groups**); 6.61, br, 1H, NH; 7.11, br, 1H, NH (**5.92, br s, 2H – single peak for NH groups**). ^{13}C ppm: 13.90 CH_3; 14.91, CH_3 (**single peak for CH$_3$ groups, 16.04**); 36.78, CH_2; 37.14, CH_2; (**35.20 – single peak for CH$_2$ groups**); 164.65 (**159.64**), CO.

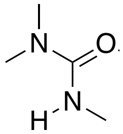

Compound 8. ^{1}H (acetone-d$_6$) ppm: **2.96** (**2.63**), d, 3H, NMe; **3.07** (**2.82**), s, 6H, NMe_2; **7.10** (**6.14**), br, 1H, NH. ^{13}C (chloroform-d) ppm: **29.77** (**27.68**), NMe; **38.33** (**36.19**), NMe; **162.04** (**160.10**), CO.

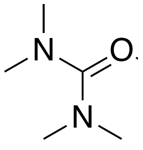

Compound 9. ^{1}H (acetone-d$_6$) ppm: **3.07** (**2.80**), s, 12H, NMe_2. ^{13}C (acetone-d$_6$) ppm: **40.17** (**38.40**), NMe_2; **164.65** (**160.60**), CO.

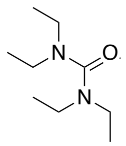

Compound 10. ^{1}H (acetone-d$_6$) ppm: **1.21** (**1.04**), t, 12H, CH_3; **3.44** (**3.11**), q, 8H, CH_2. ^{13}C (acetone-d$_6$) ppm: **13.12** (**13.49**), CH_3; **49.79** (**42.86**), CH_2; **164.98** (**164.77**), CO.

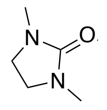

Compound 11. ^{1}H (acetone-d$_6$) ppm: **3.00** (**2.63**), s, 6H, NMe; **3.71** (**3.18**), s, 4H, CH_s. ^{13}C (acetone-d$_6$) ppm: **32.86** (31.58), CH_2; **47.26** (**45.42**), NMe; **162.68** (**162.15**), CO.

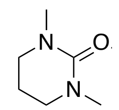

Compound 12. ^{1}H (acetone-d_6) ppm: **2.04** (**1.90**), m, 2H, CH_2; **3.08** (**2.77**), s, 6H, NMe; **3.47** (**3.19**), s, 4H, CH_2. ^{13}C (acetone-d_6) ppm: **20.67** (**22.94**), CH_2; **37.90** (**35.38**), CH_2; **48.79** (**48.37**), NMe; **159.02** (**156.74**), CO.

Table S1. Select VT NMR data, compound **7**

T (°C)	N-H(A)	N-H(B)	CH_2(A)	CH_2(B)
20	6.46	6.05	3.32	3.24
25	6.49	6.08	3.35	3.28
35	6.60	6.20	3.45 - coalesced	
45	6.67	6.31	3.56 - coalesced	
55	6.67	6.47	3.66 - coalesced	
65	6.65 - coalesced		3.75 - coalesced	
75	6.72 - coalesced		3.85 - coalesced	
85	6.78 - coalesced		3.93 - coalesced	
20 (re-cooled)	6.48	6.08	3.34	3.27

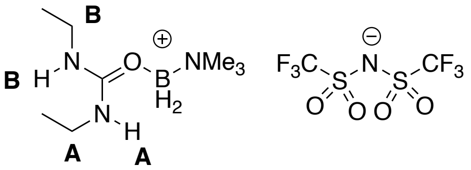

"A" and "B" assignments made using computational modelling. See main text.

\

Table S2. Computed NBO spin charge values

Cations - structures and numbers (below)

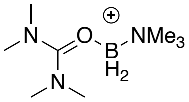

9

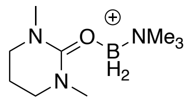

12

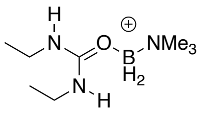

7

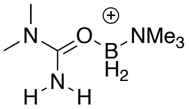

6

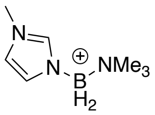

A

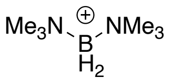

B

9	12	7	6	A	B
+0.765	+0.765	+0.761	+0.799	+0.813	+0.865

Above: NBO spin charge on B in complex with Tf_2N^-. Compounds A and B have been made in previous work.[2,23,45]

Cyclic Voltammograms (Tf_2N^- salts)

Compound 1

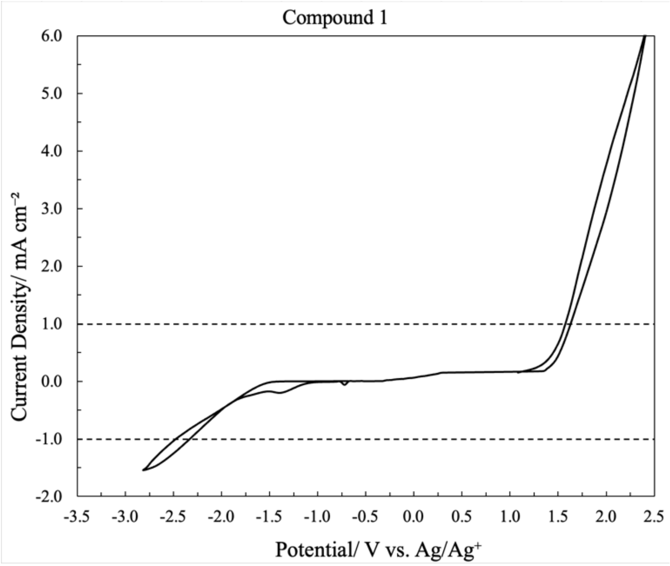

Compounds 2-5

No CV data. Solids at ambient T.

Compound 6

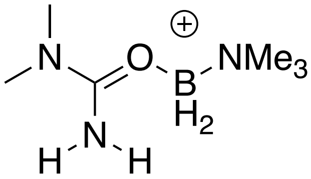

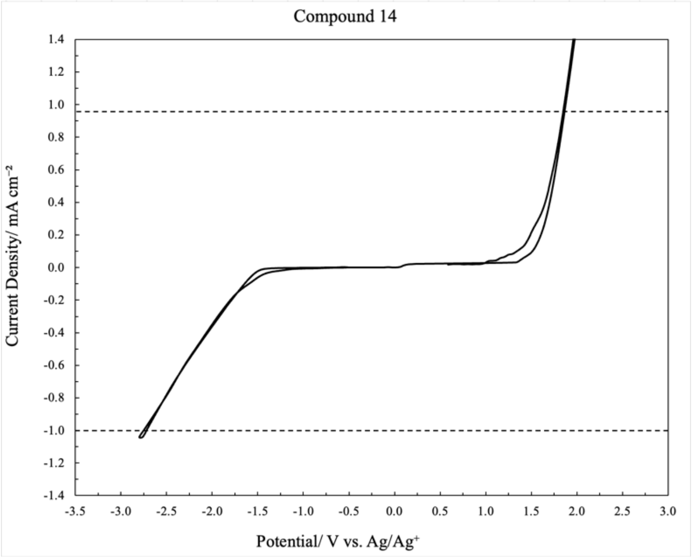

Compound 7

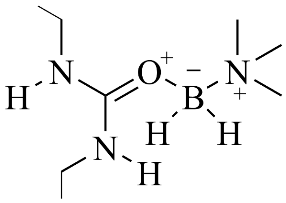

N
H
O
+
−
B
N
+
H
H
N
H

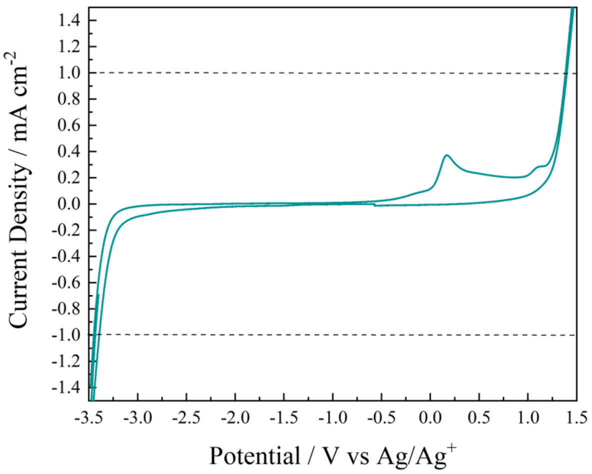

Current Density / mA cm-2
Potential / V vs Ag/Ag+
1.4
1.2
1.0
0.8
0.6
0.4
0.2
0.0
-0.2
-0.4
-0.6
-0.8
-1.0
-1.2
-1.4
-3.5
-3.0
-2.5
-2.0
-1.5
-1.0
-0.5
0.0
0.5
1.0
1.5

Compound 8

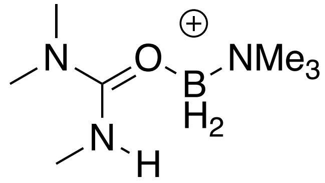

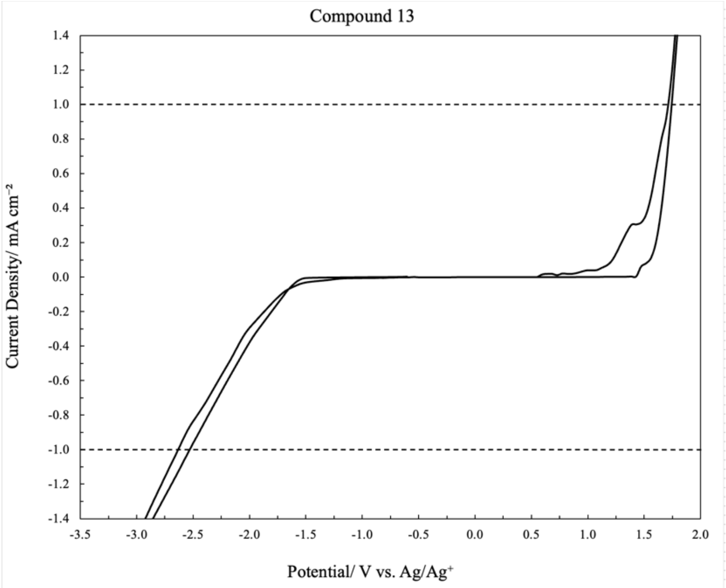

Compound 9

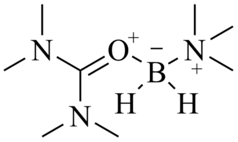

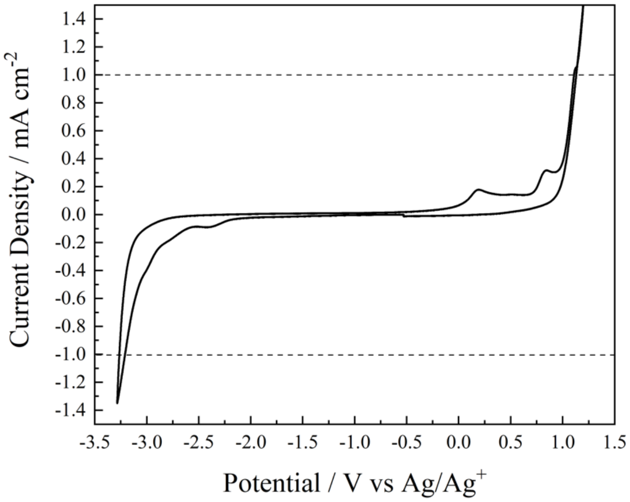

Compound 10

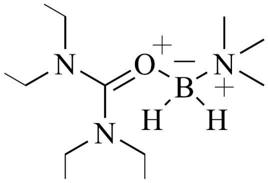

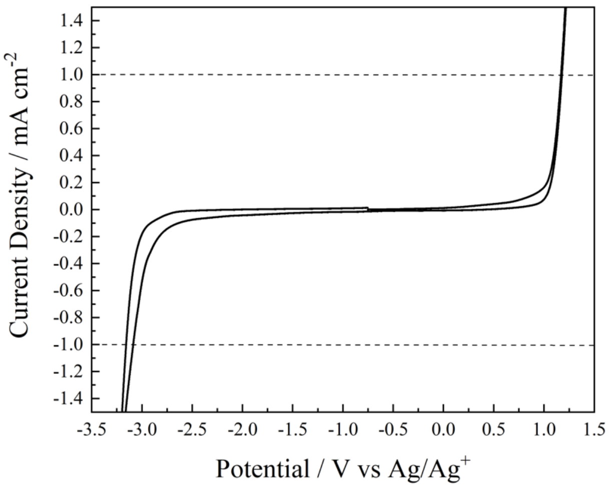

Compound 11

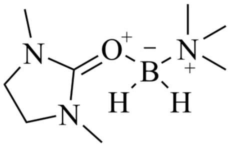

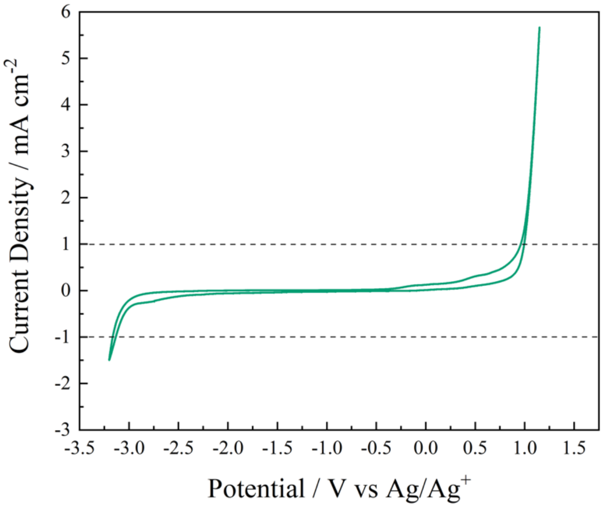

Compound 12

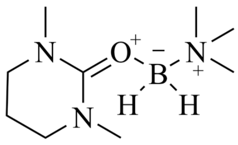

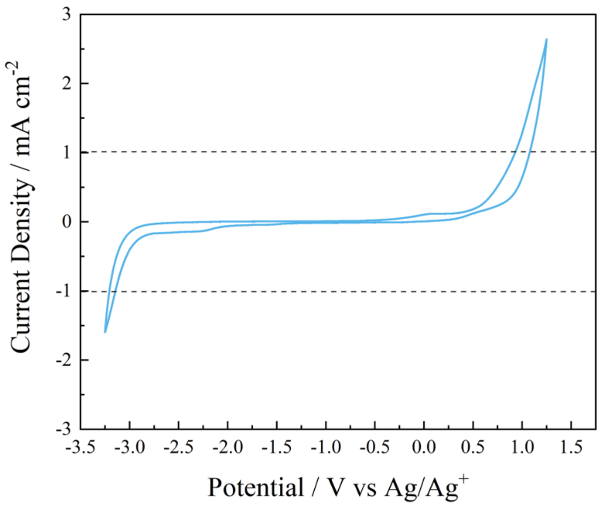

Differential Scanning Calorimetry (DSC)

Compound 1

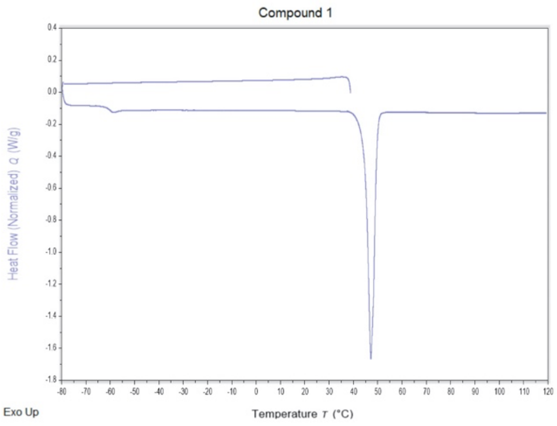

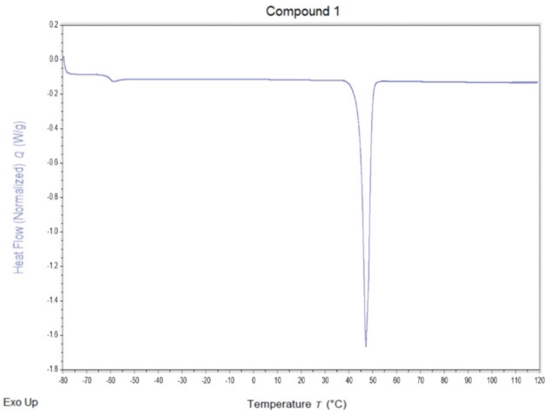

Compound 2

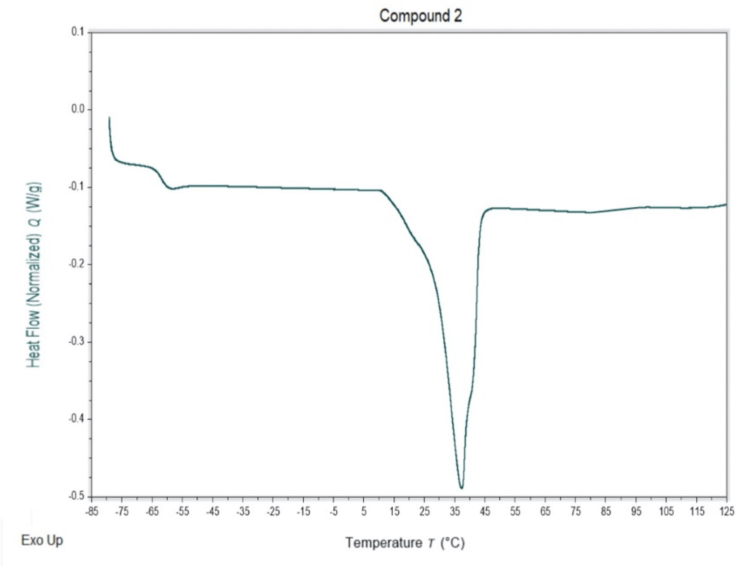
Compound 2
Heat Flow (Normalized) Q (W/g)
Temperature T (°C)
Exo Up

Compound 3

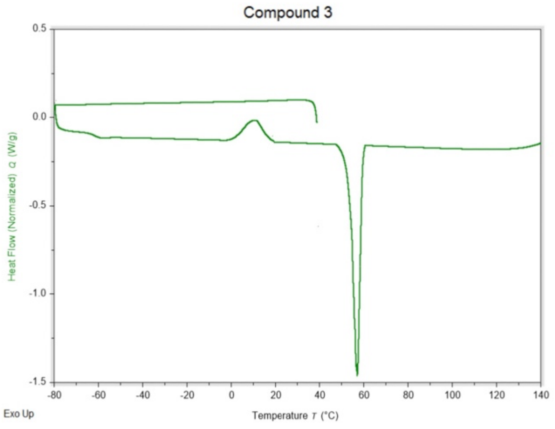
Compound 3
Heat Flow (Normalized) Q (W/g)
Temperature T (°C)
Exo Up

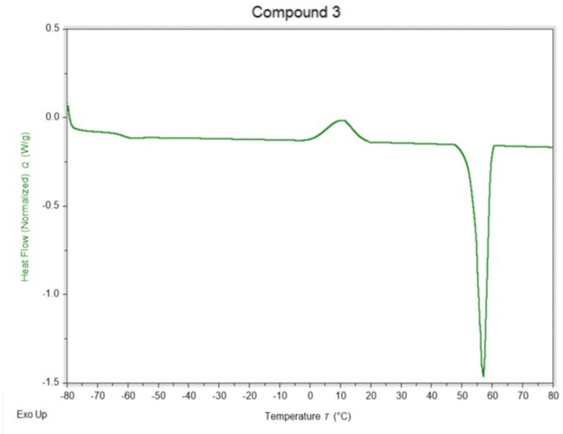
Compound 3
Heat Flow (Normalized) Q (W/g)
Temperature T (°C)
Exo Up

Compound 4

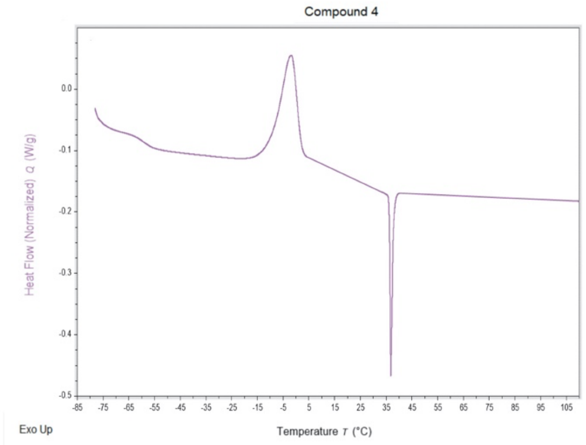

Compound 5

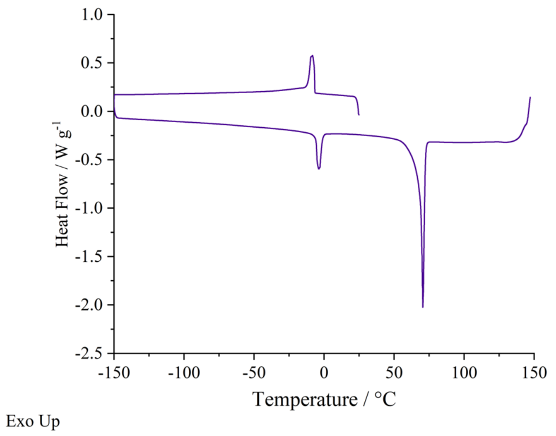

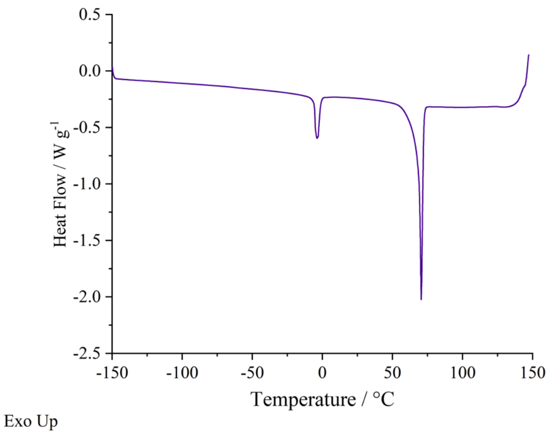

Compound 6

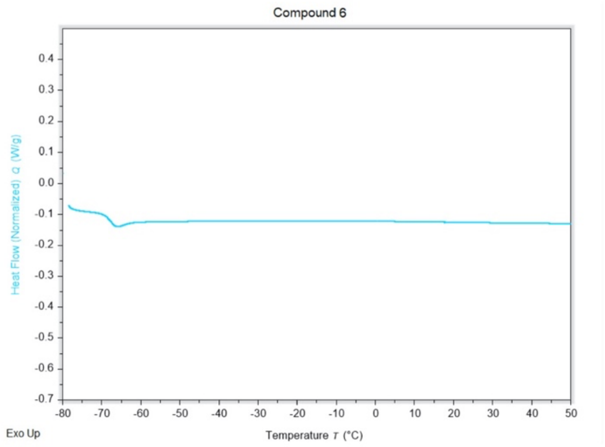

Compound 7

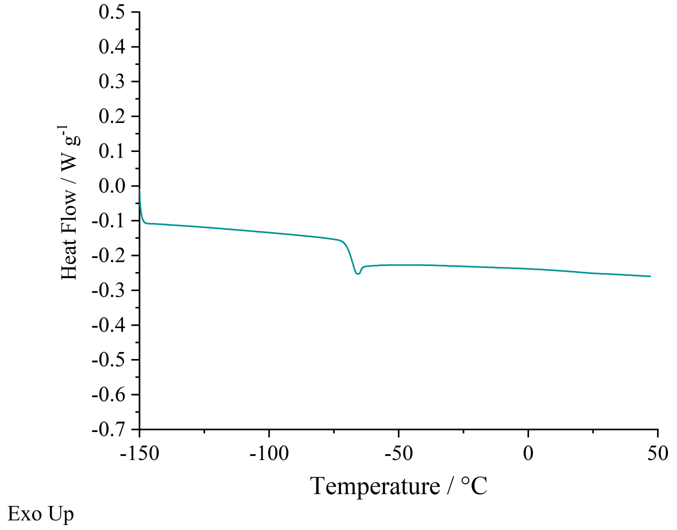

Compound 8

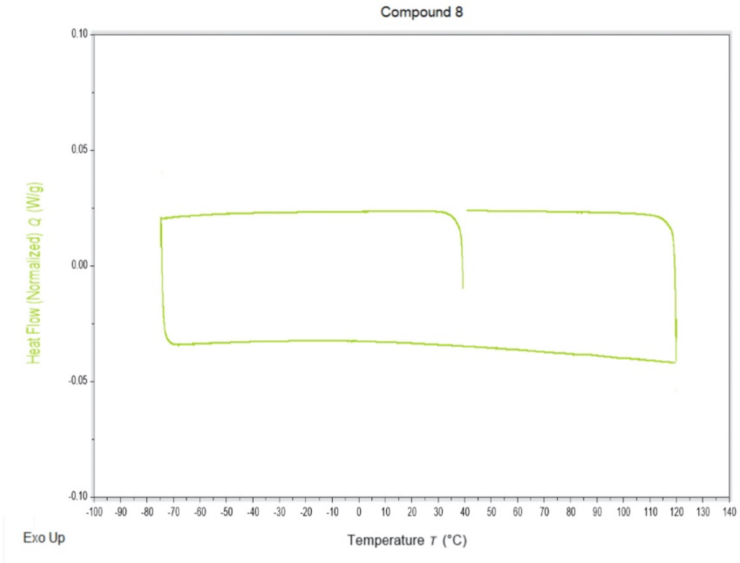

Compound 9

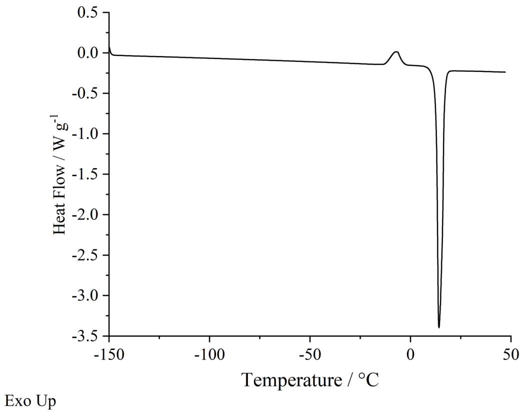

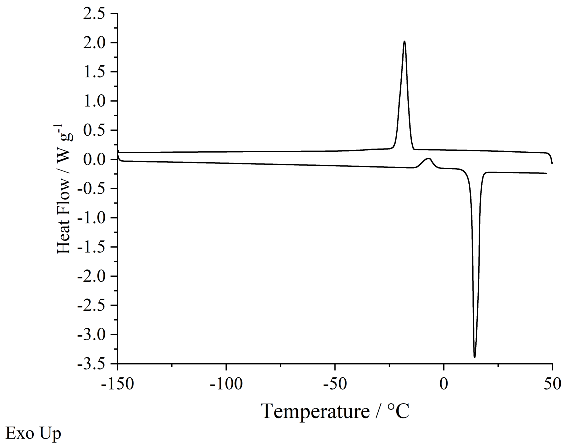

Compounds 10-12

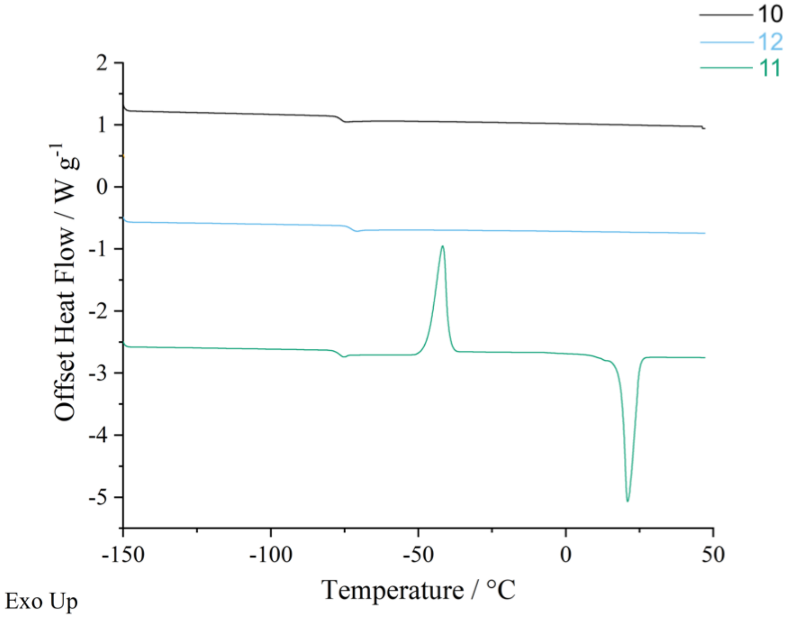

Thermogravimetric analysis (TGA)

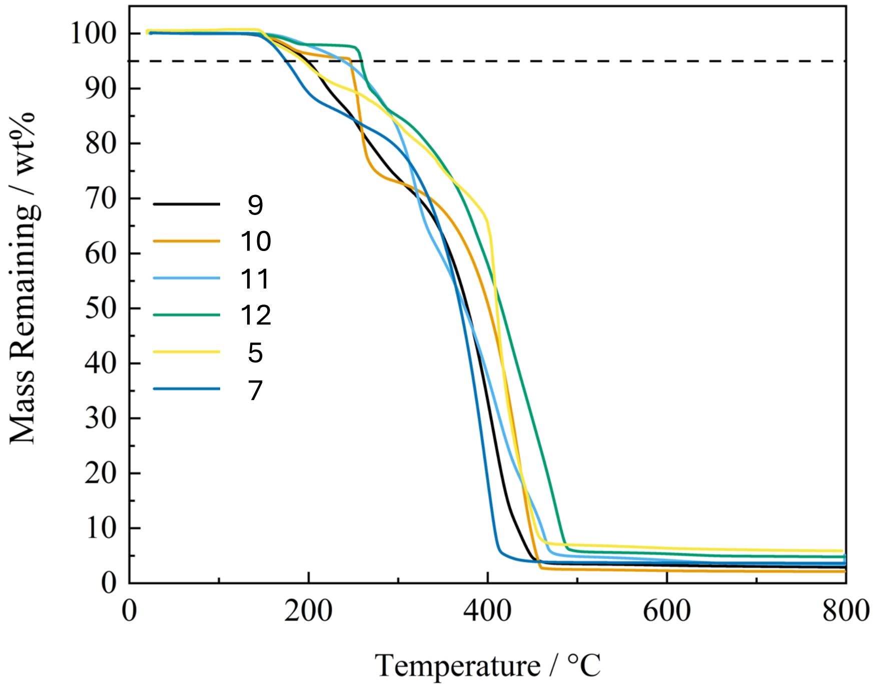

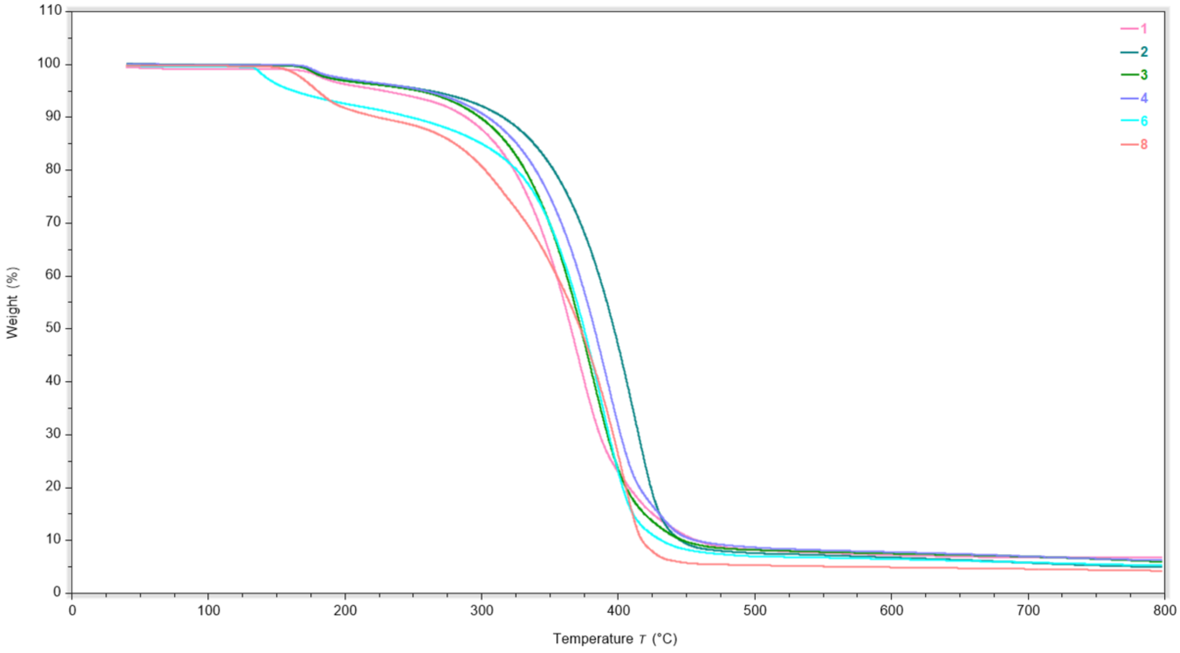

pH-dependent aqueous stability study, Compound 10 (iodide salt)

Baseline reference spectra, pH = 8

^{1}H

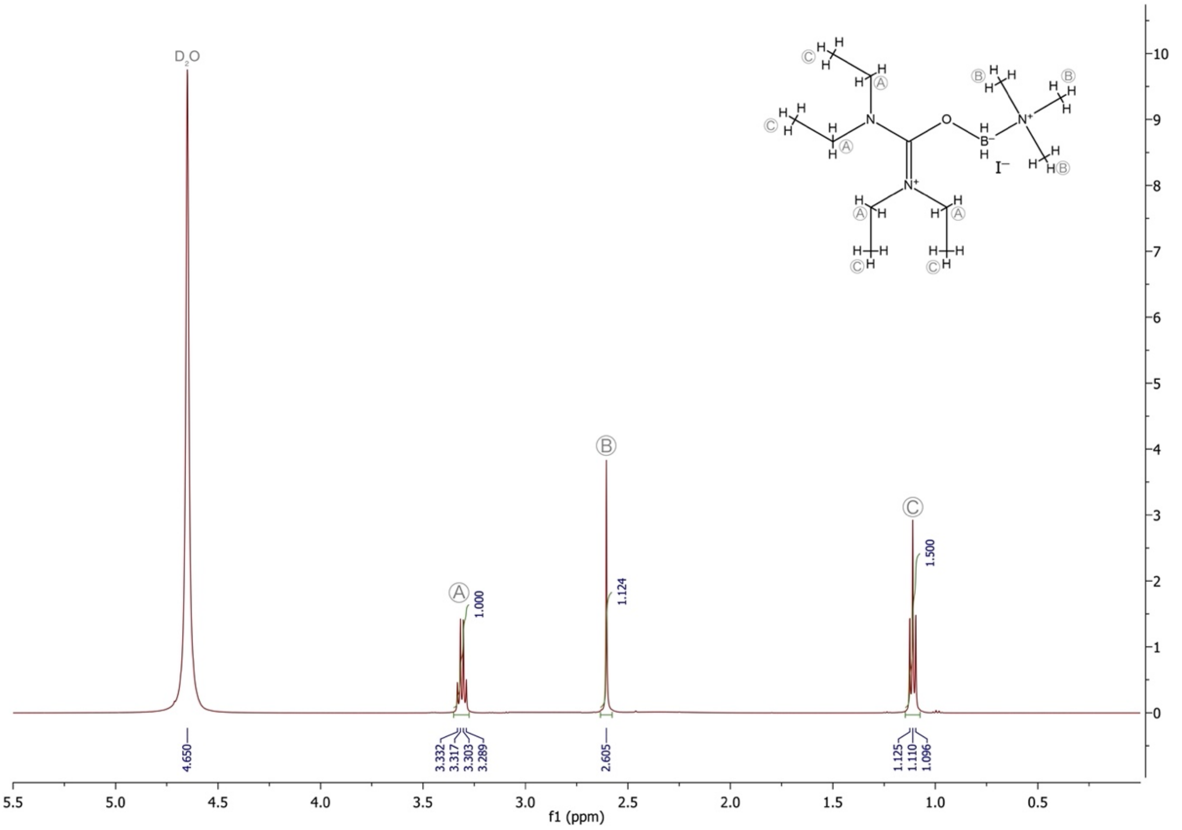

^{13}C

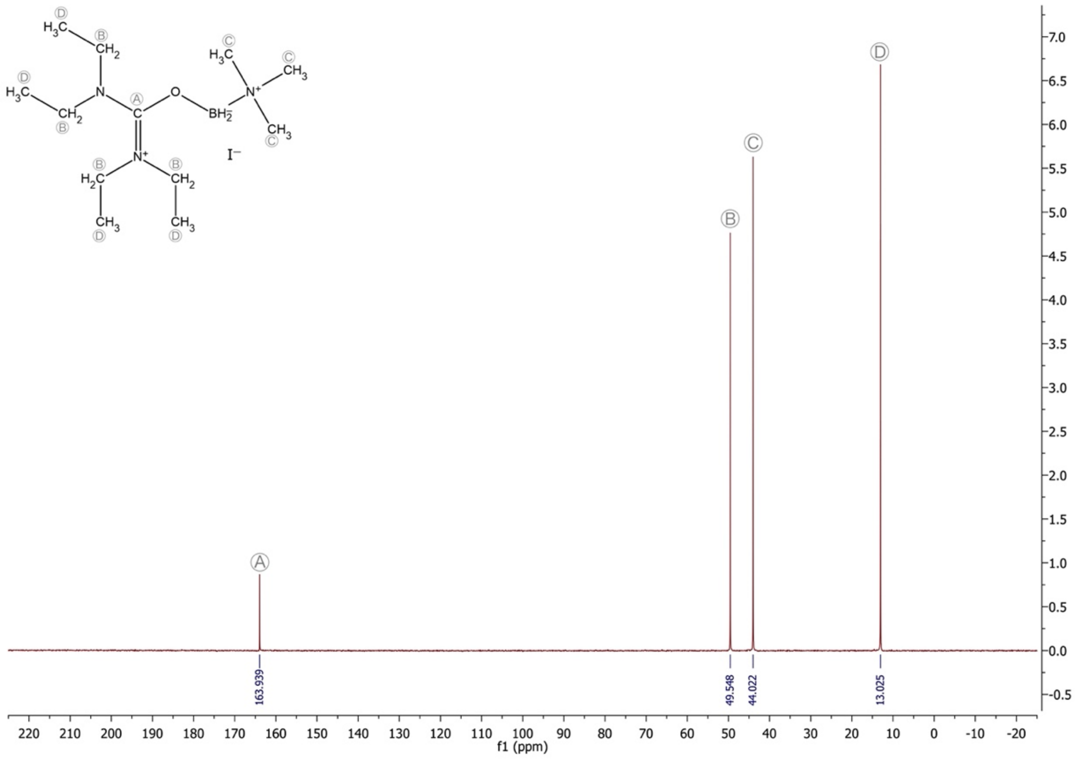

^{10}B

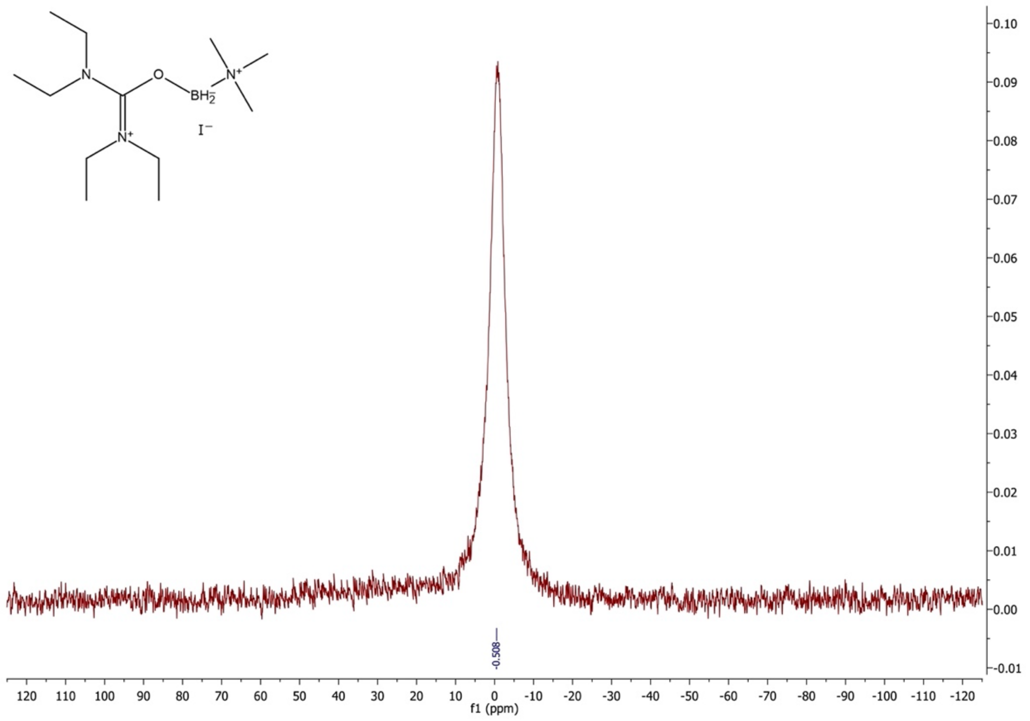

[1]H, pH = 1-14, immediately after dissolution

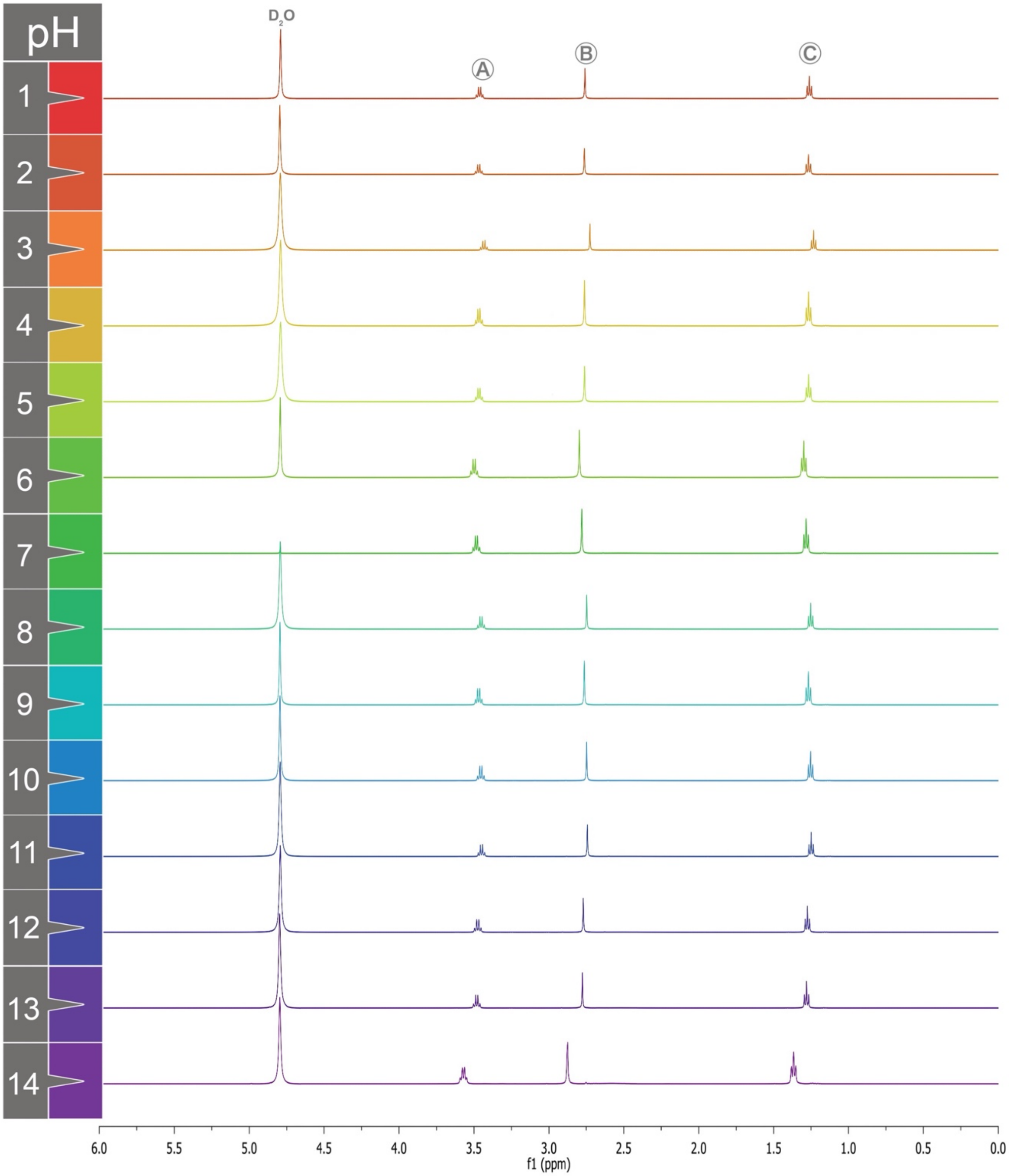

^{13}C, pH = 1-14, immediately after dissolution

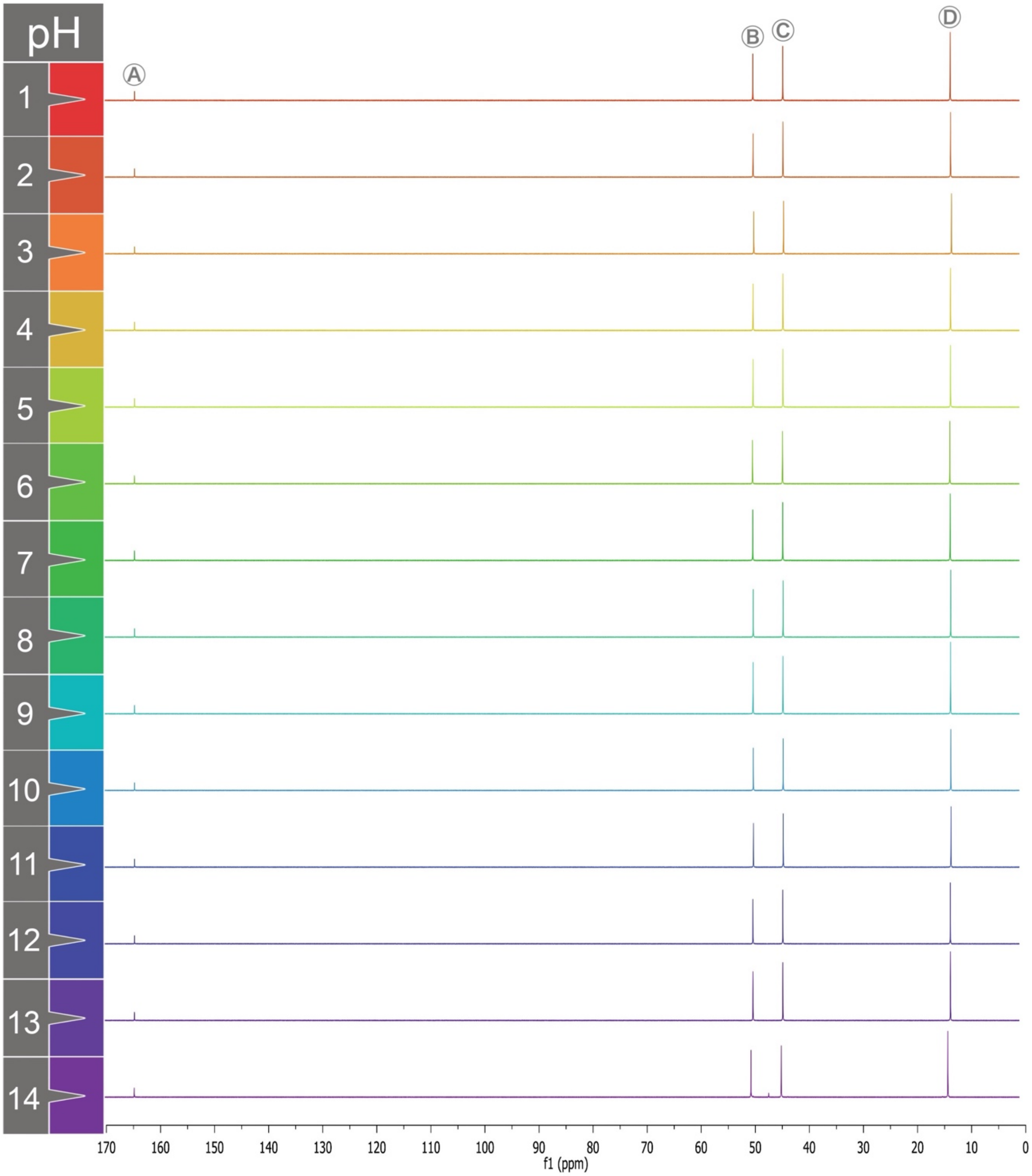

^{10}B, pH = 1-14, immediately after dissolution

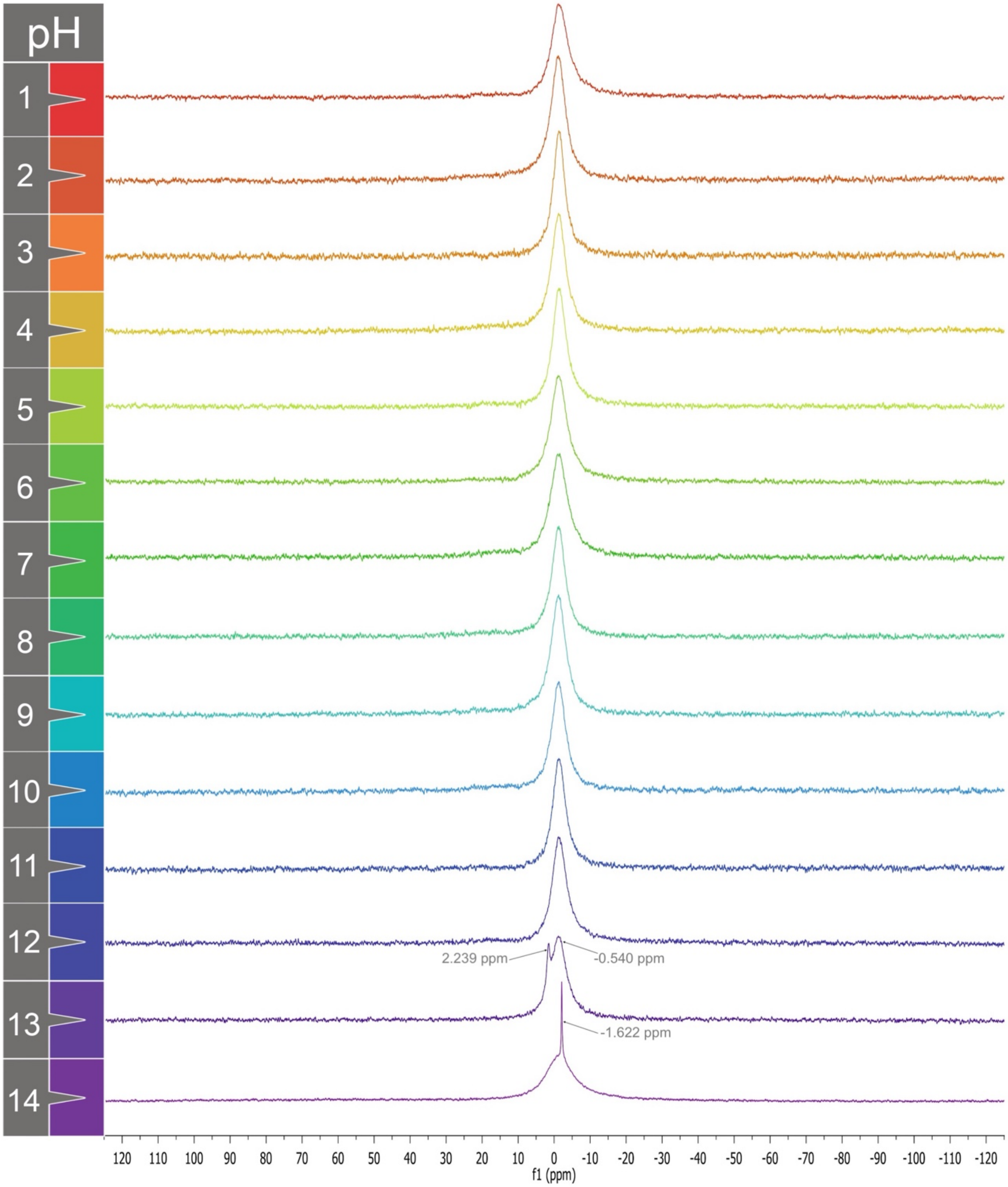

[1]H, pH = 1-14, at 1 week

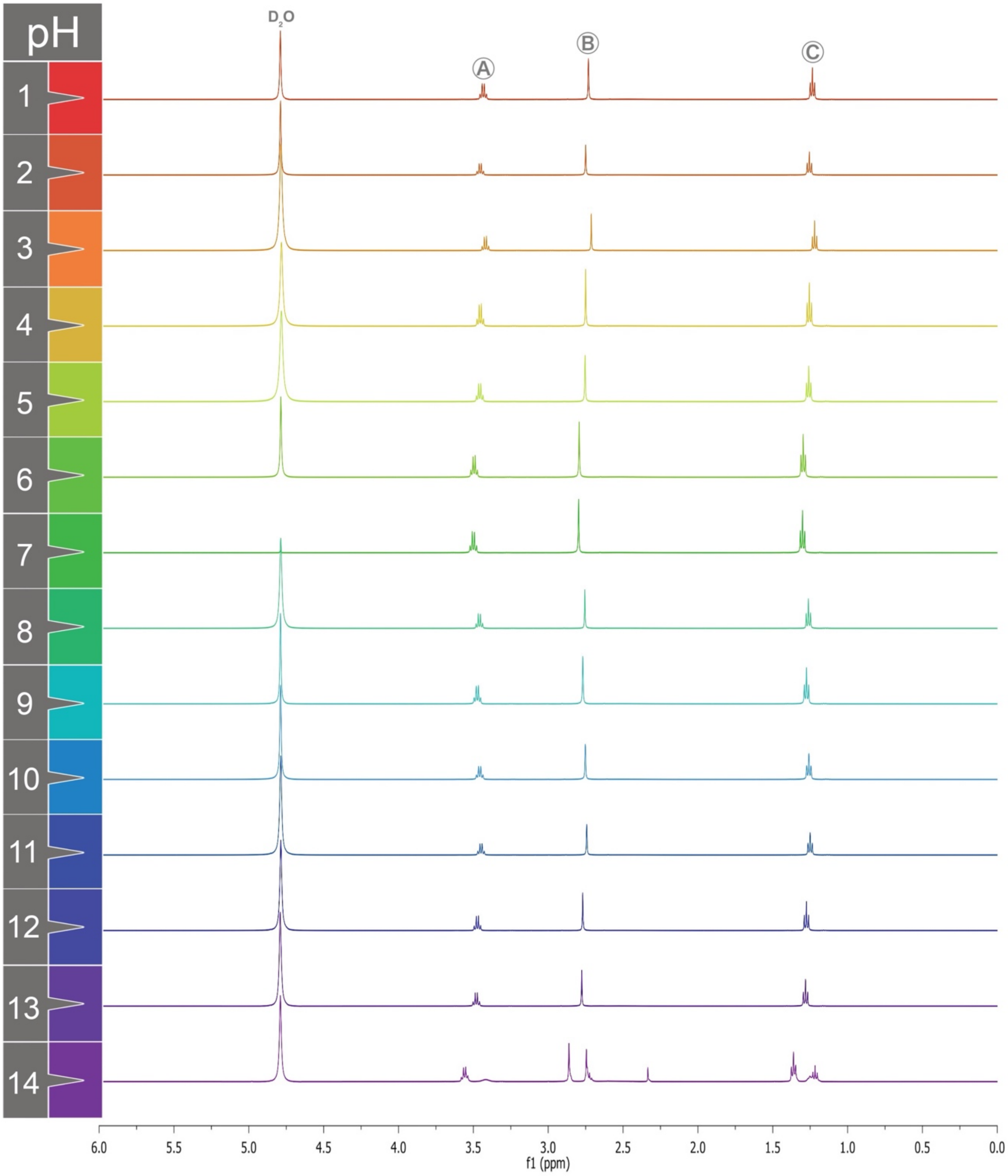

^{13}C, pH = 1-14, at 1 week

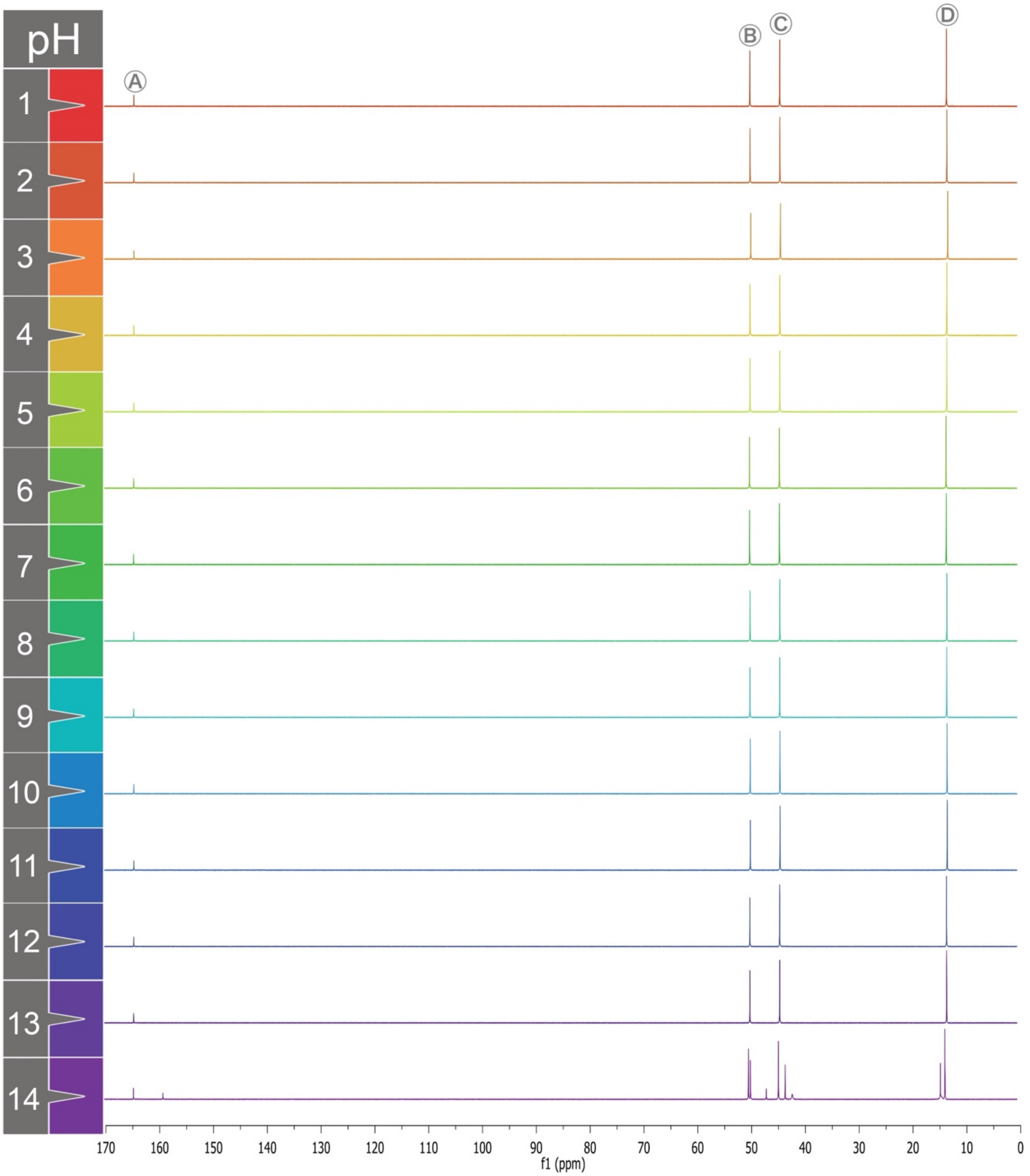

^{10}B, pH = 1-14, at 1 week

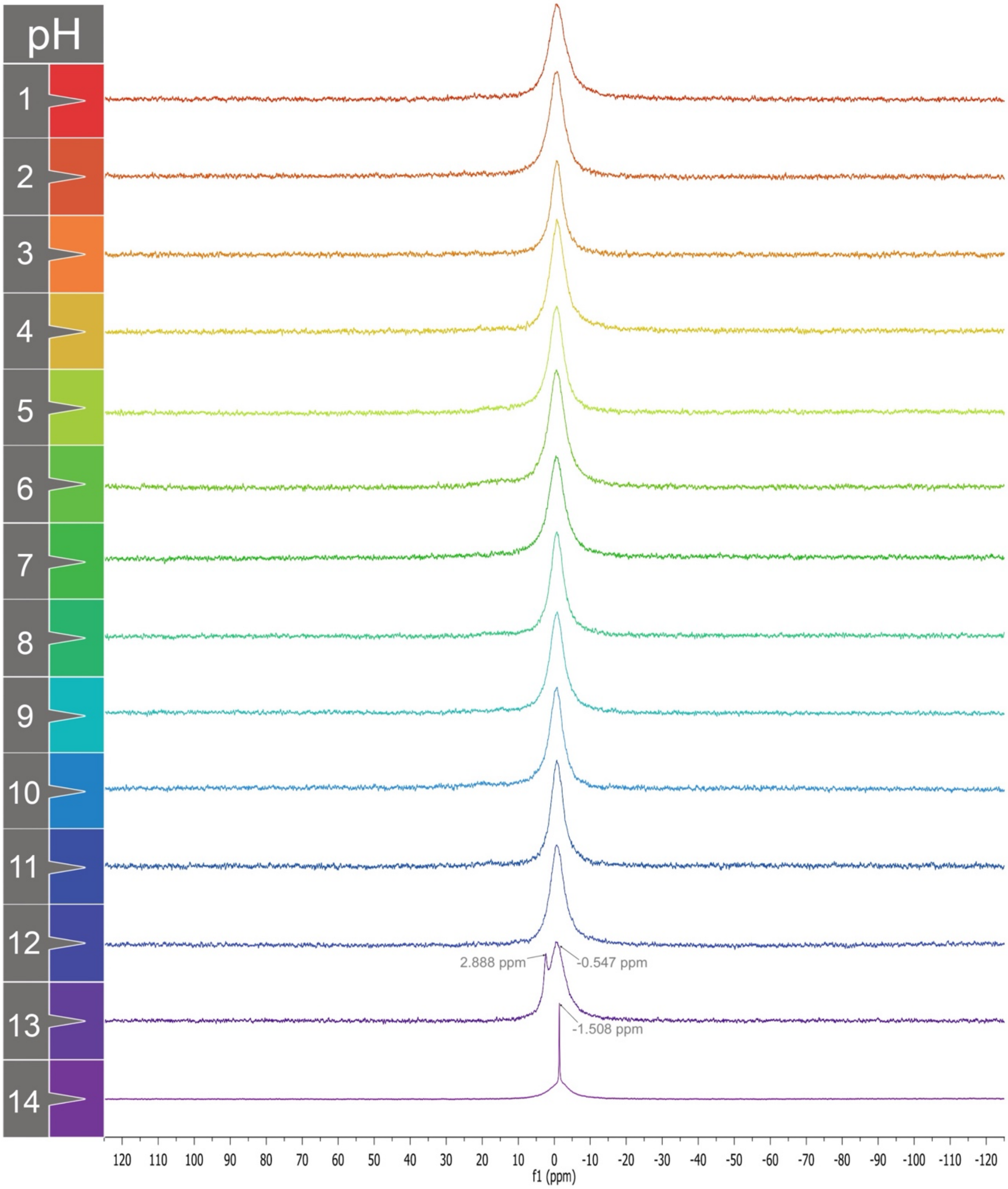

Change in the ^{10}B spectrum at pH = 14 over 1 week

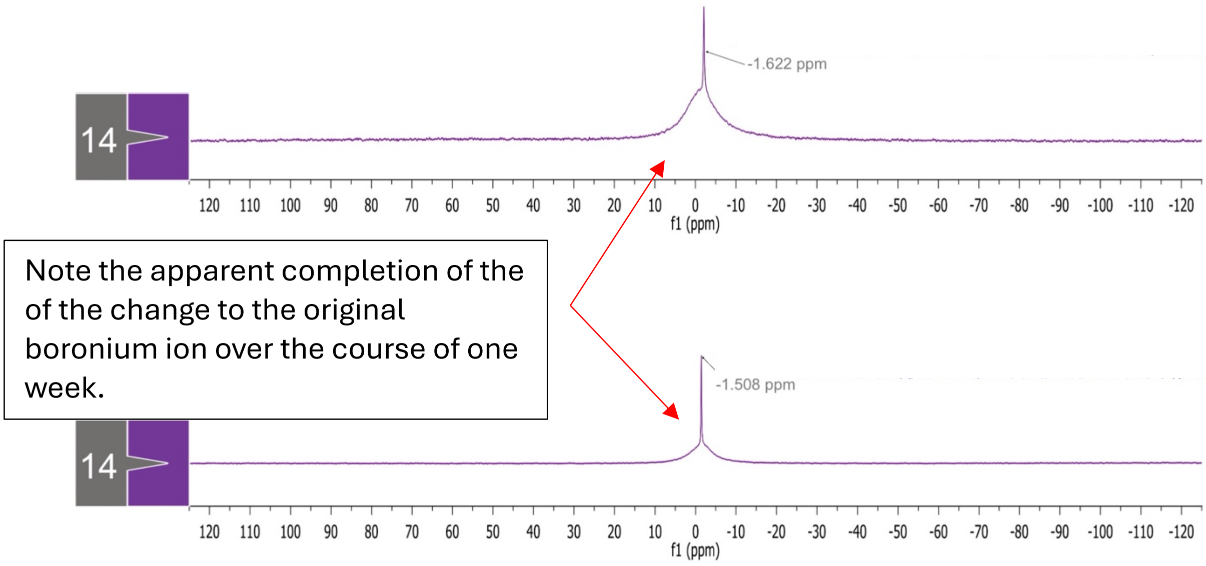

pH-dependent aqueous stability study, Compound 2 (iodide salt)

Baseline reference spectra, pH = 8

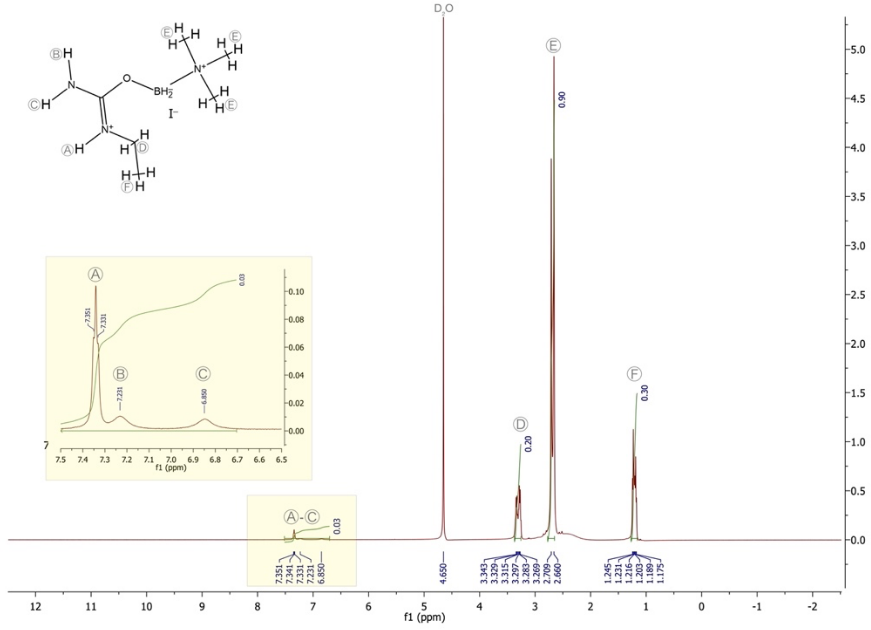

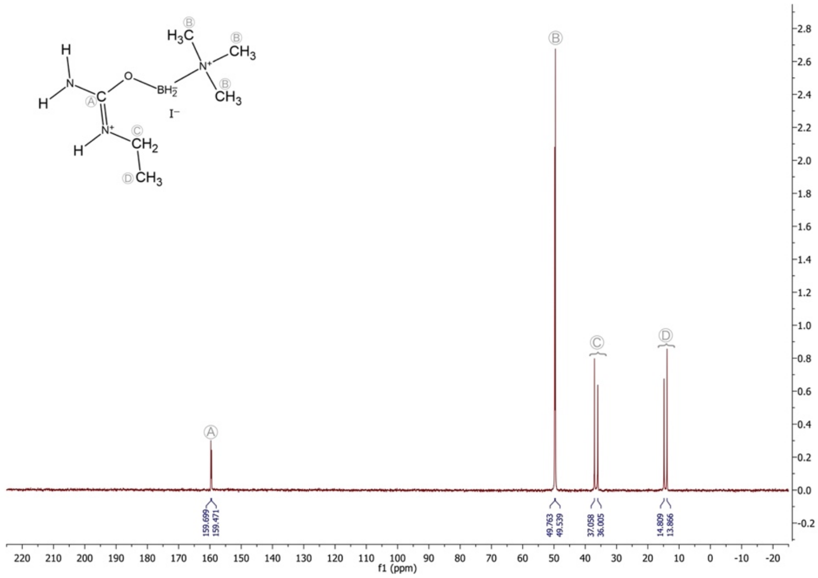

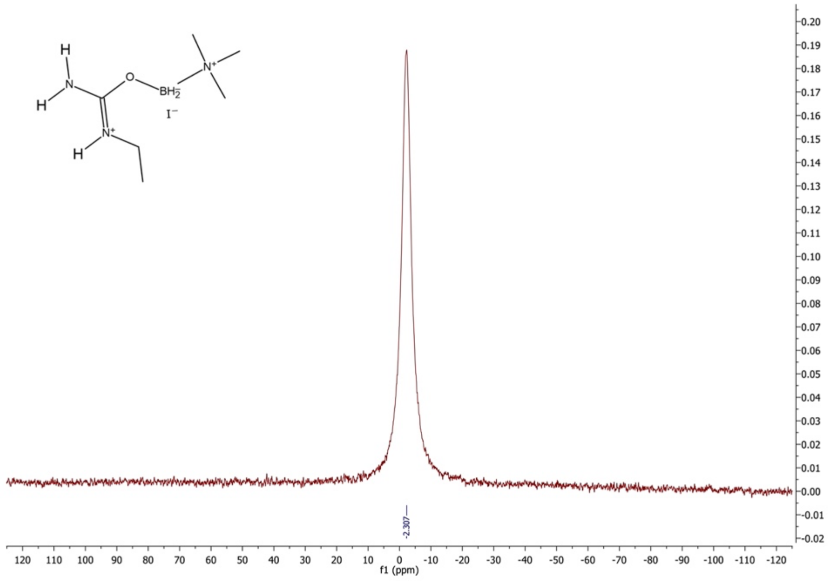

BH2−
I−
N+
-2.307
f1 (ppm)

[1]H, pH = 1-14, immediately after dissolution

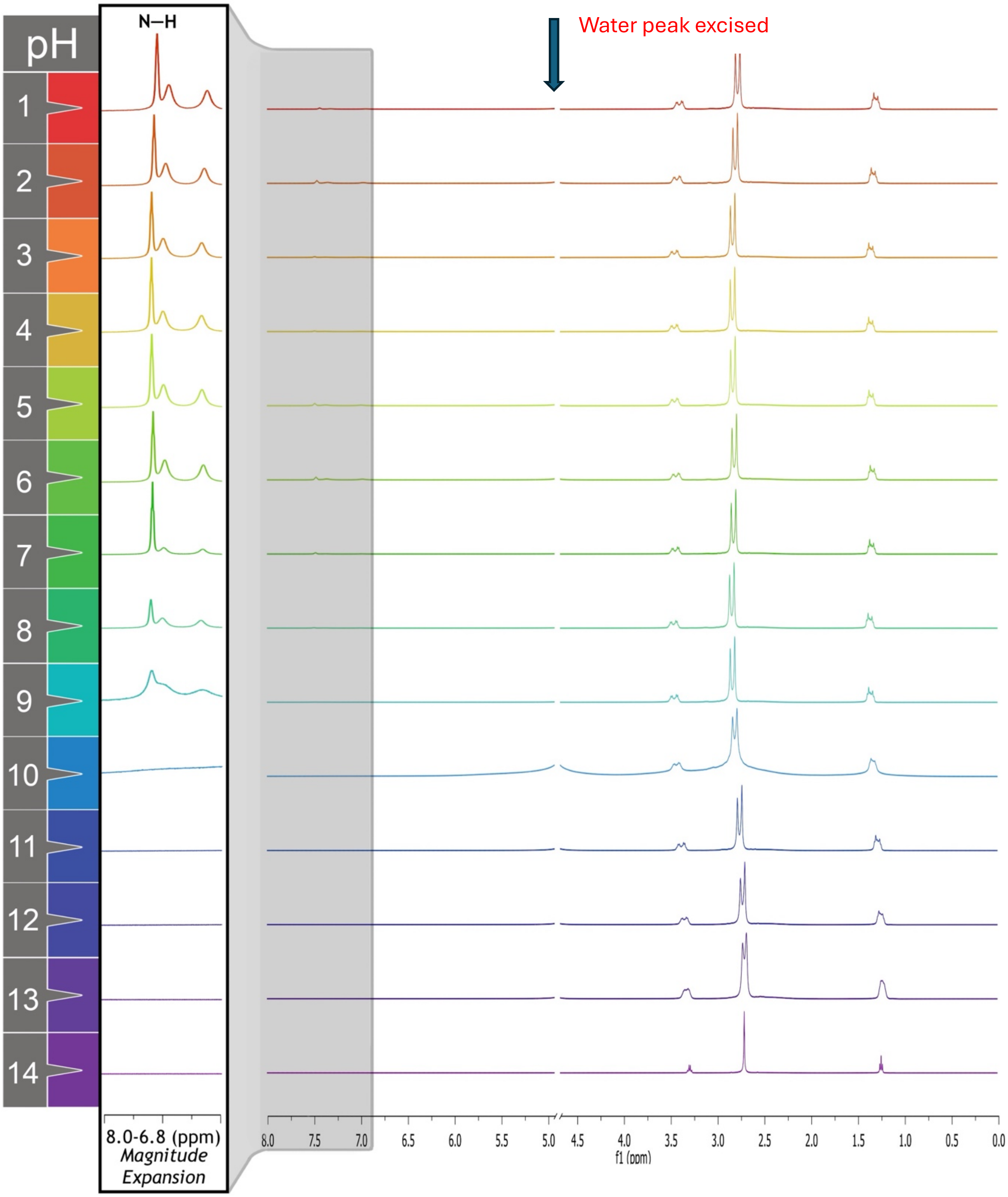

^{10}B, pH = 1-14, immediately after dissolution

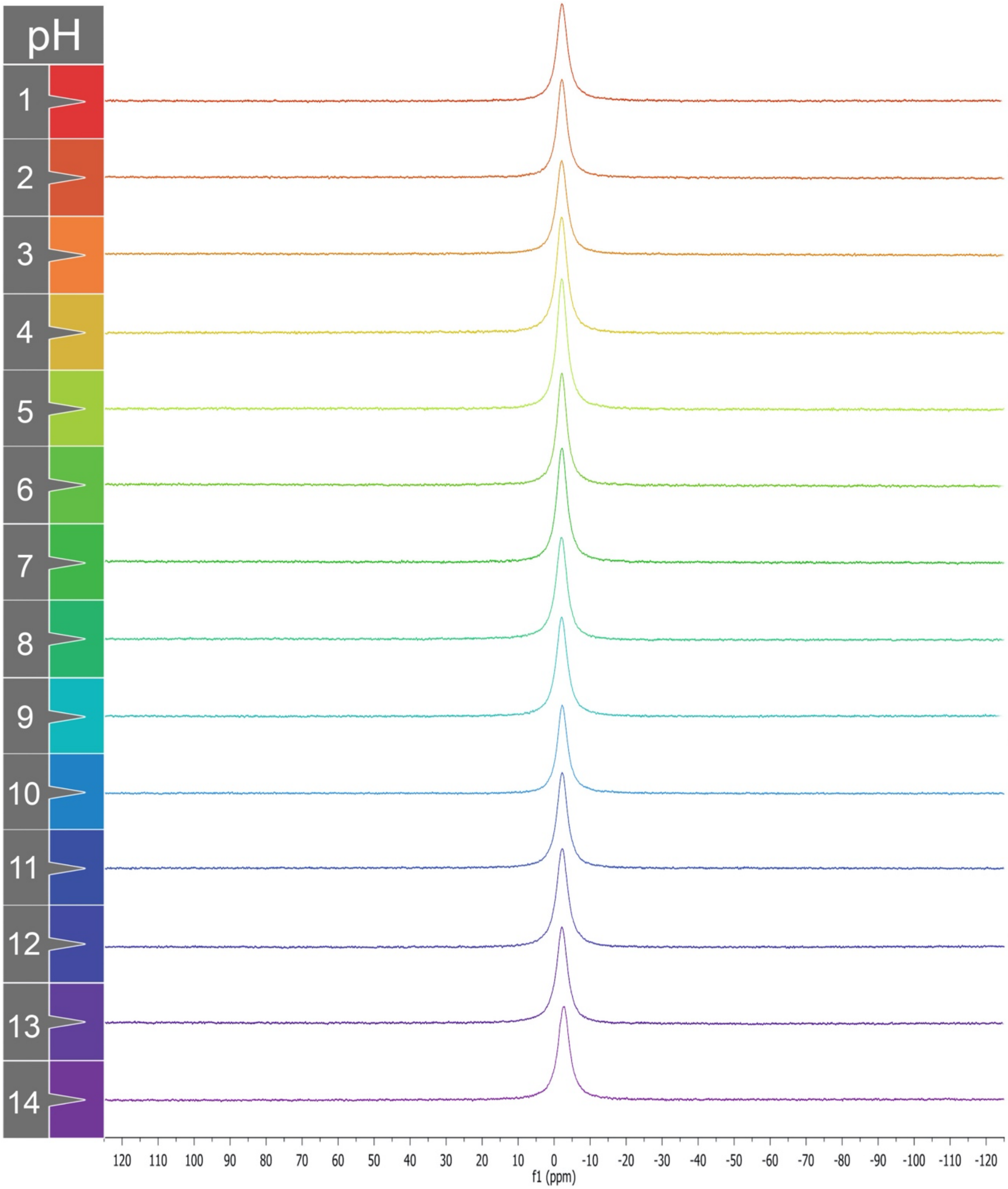

^{13}C, pH = 1-14, immediately after dissolution

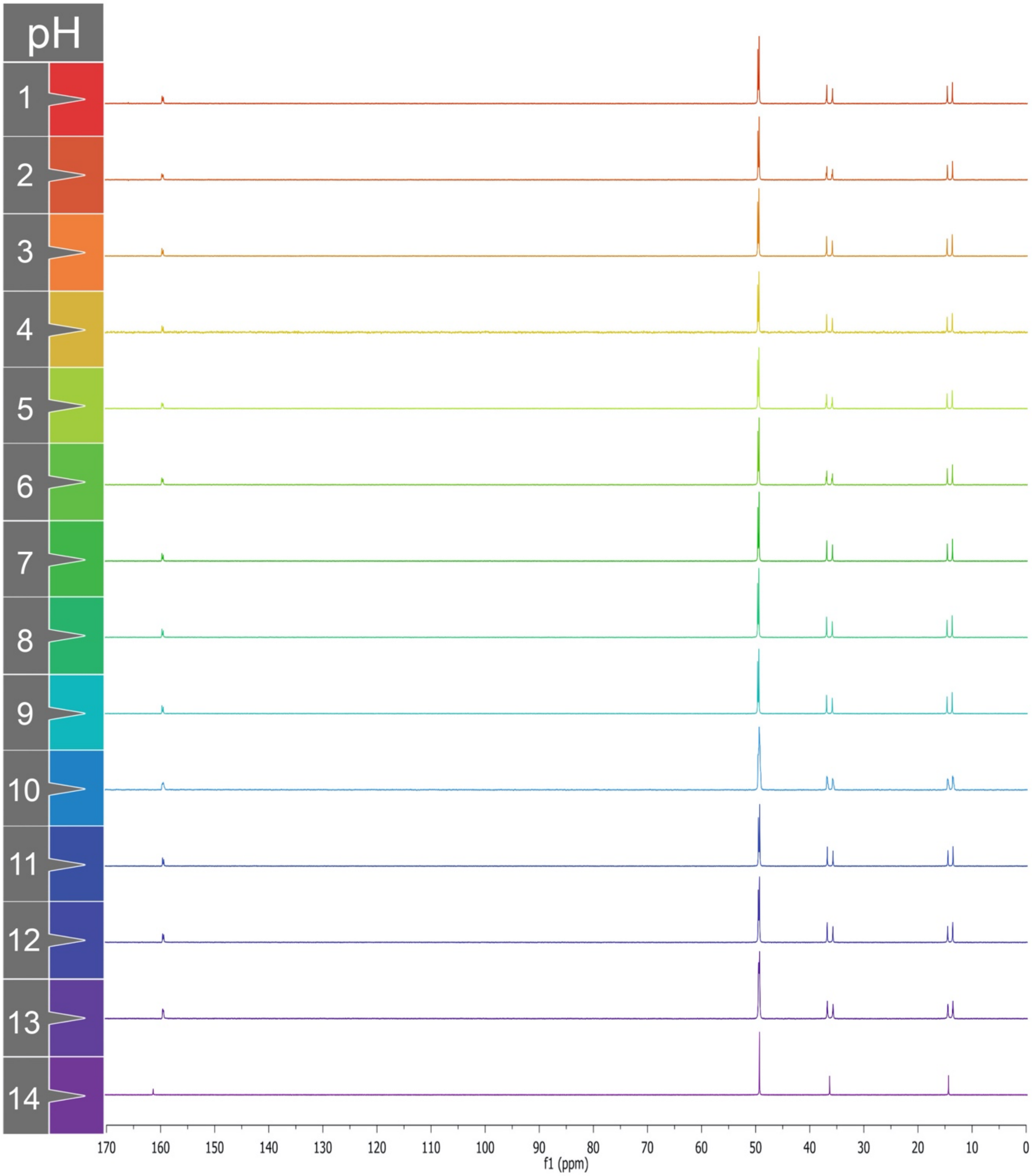

^1H, pH = 1-14, after 1 week

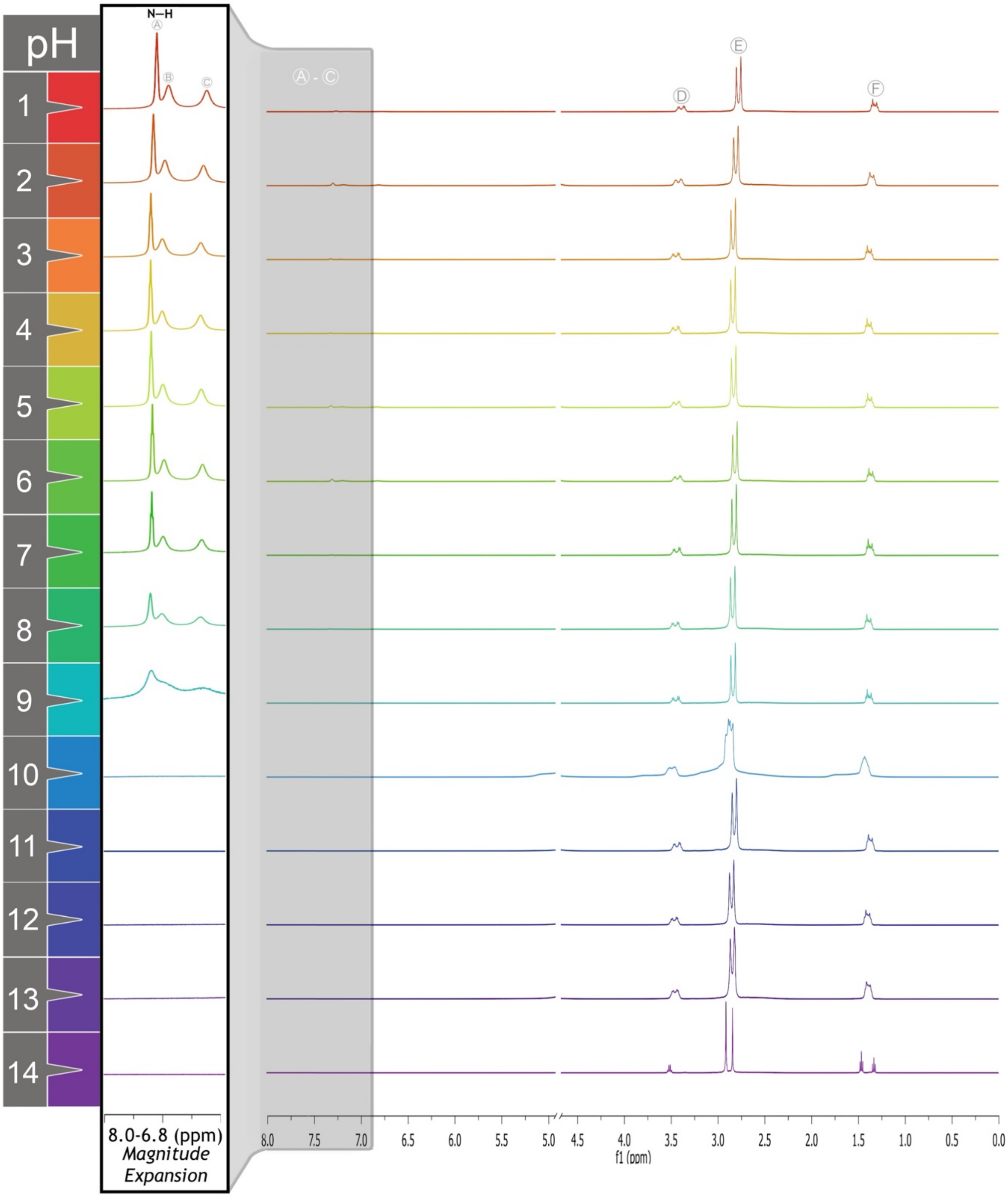

^{13}C, pH = 1-14, after 1 week

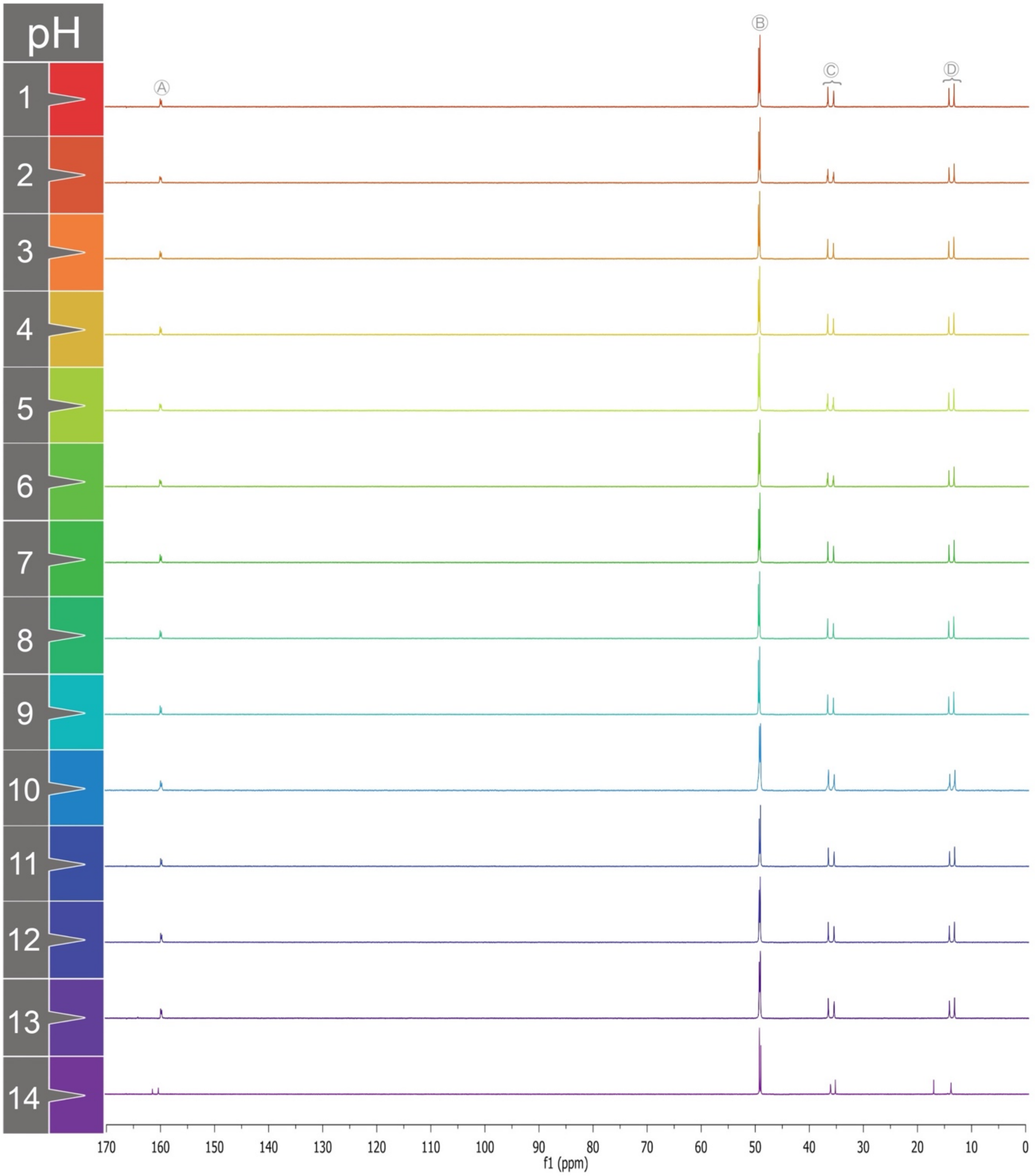

^{10}B, pH = 1-14, after 1 week

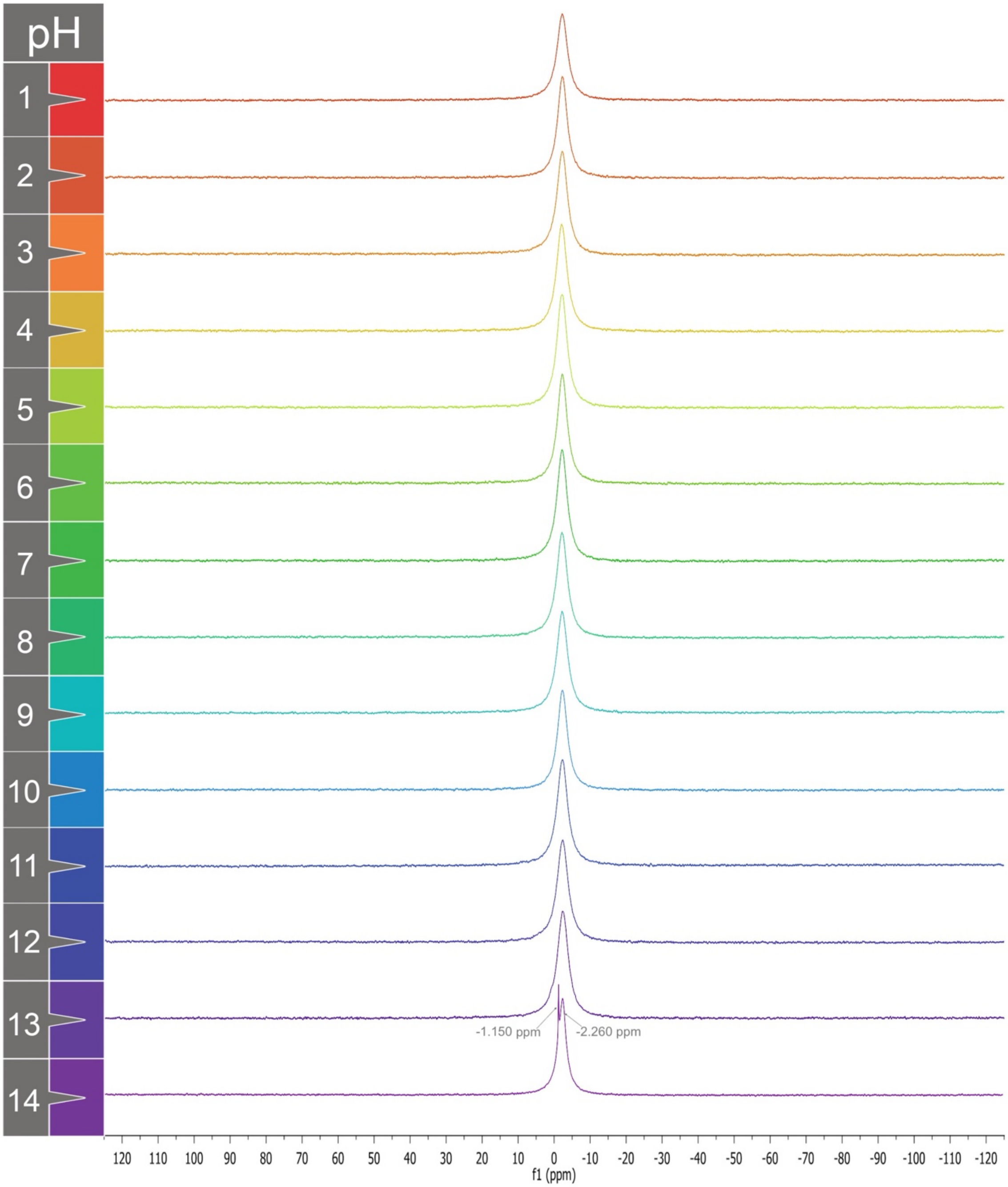

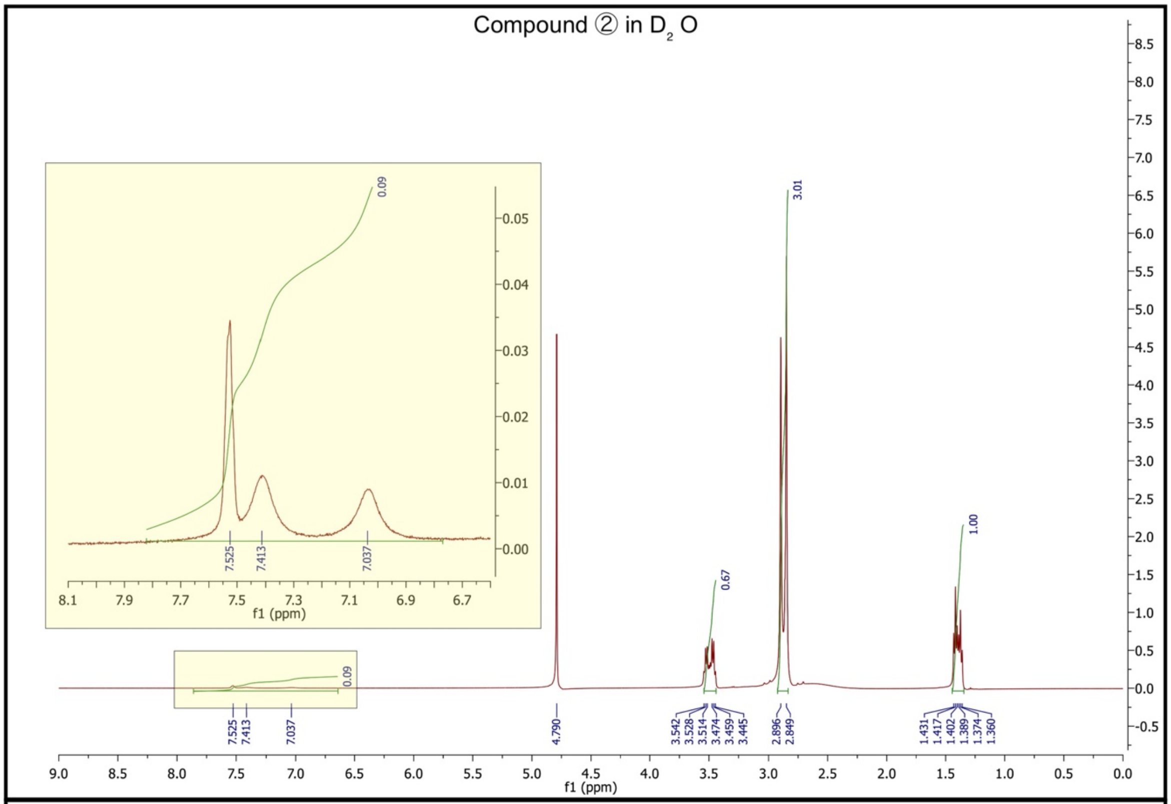
Compound ② in D_2O
7.525
7.413
7.037
0.09
8.1 7.9 7.7 7.5 7.3 7.1 6.9 6.7
f1 (ppm)
0.05 0.04 0.03 0.02 0.01 0.00
4.790
3.542 3.528 3.514 3.474 3.459 3.445
0.67
2.896 2.849
3.01
1.431 1.417 1.402 1.389 1.374 1.360
1.00
9.0 8.5 8.0 7.5 7.0 6.5 6.0 5.5 5.0 4.5 4.0 3.5 3.0 2.5 2.0 1.5 1.0 0.5 0.0
f1 (ppm)

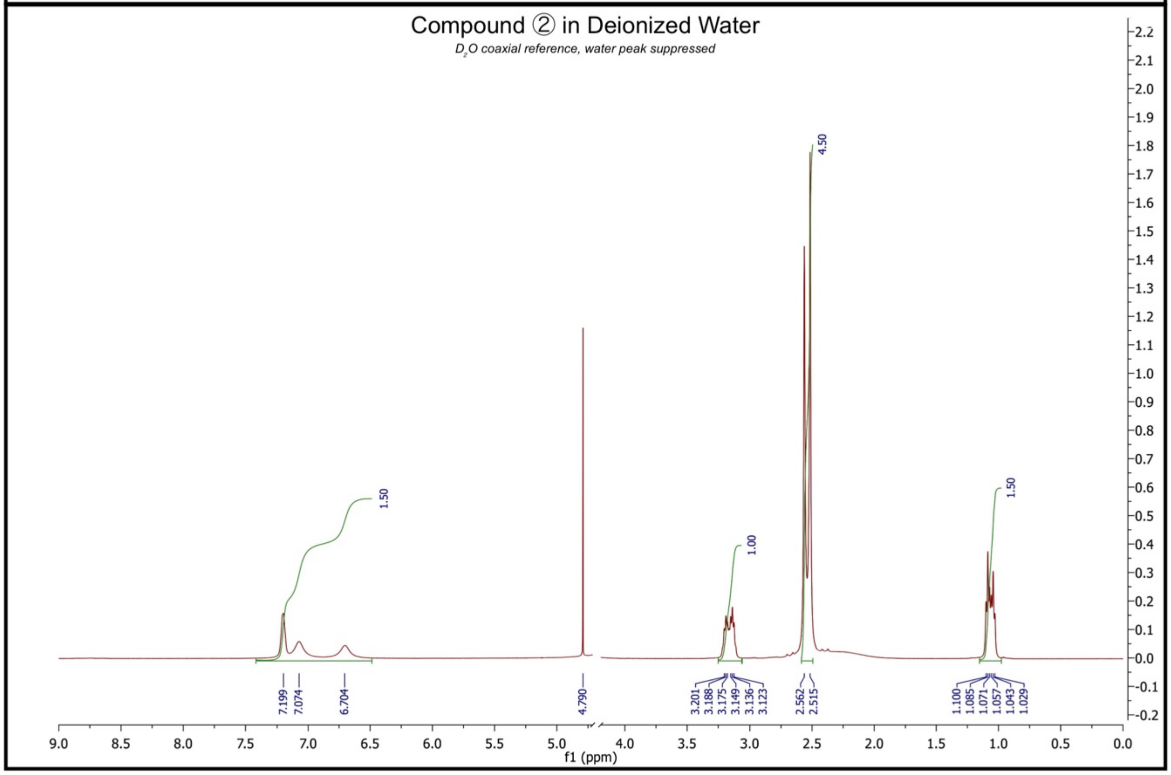
Compound ② in Deionized Water
D_2O coaxial reference, water peak suppressed
7.199 7.074 6.704
1.50
4.790
3.201 3.188 3.175 3.149 3.136 3.123
1.00
2.562 2.515
4.50
1.100 1.085 1.071 1.057 1.043 1.029
1.50
9.0 8.5 8.0 7.5 7.0 6.5 6.0 5.5 5.0 4.0 3.5 3.0 2.5 2.0 1.5 1.0 0.5 0.0
f1 (ppm)

checkCIF (basic structural check) running

Checking for embedded fcf data in CIF ...
Found embedded fcf data in CIF. Extracting fcf data from uploaded CIF, please wait . . .

checkCIF/PLATON (basic structural check)

Structure factors have been supplied for datablock(s) JD_02_01_24_0m

THIS REPORT IS FOR GUIDANCE ONLY. IF USED AS PART OF A REVIEW PROCEDURE FOR PUBLICATION, IT SHOULD NOT REPLACE THE EXPERTISE OF AN EXPERIENCED CRYSTALLOGRAPHIC REFEREE.

No syntax errors found. CIF dictionary
Please wait while processing .... Interpreting this report

Structure factor report

Datablock: JD_02_01_24_0m

Bond precision:	N- C = 0.0016 A		Wavelength=0.71073
Cell:	a=10.2265(5)	b=9.5381(5)	c=14.3703(6)
	alpha=90	beta=95.782(2)	gamma=90
Temperature:	150 K		

	Calculated	Reported
Volume	1394.57(12)	1394.57(12)
Space group	P 21/c	P 21/c
Hall group	-P 2ybc	-P 2ybc
Moiety formula	C8 H23 B N3 O, I	C8 H23 B N3 O, I
Sum formula	C8 H23 B I N3 O	C8 H23 B I N3 O
Mr	315.00	315.00
Dx,g cm-3	1.500	1.500
Z	4	4
Mu (mm-1)	2.276	2.276
F000	632.0	632.0
F000'	630.27	
h,k,lmax	15,14,22	15,14,22
Nref	5346	5324
Tmin,Tmax	0.488,0.620	0.598,0.747
Tmin'	0.478	

Correction method= # Reported T Limits: Tmin=0.598 Tmax=0.747 AbsCorr = MULTI-SCAN

Data completeness= 0.996 Theta(max)= 33.187

R(reflections)= 0.0169(4540) wR2(reflections)= 0.0373(5324)

S = 1.038 Npar= 136

The following ALERTS were generated. Each ALERT has the format
test-name_ALERT_alert-type_alert-level.
Click on the hyperlinks for more details of the test.

Alert level G

PLAT910_ALERT_3_G Missing # of FCF Reflection(s) Below Theta(Min). 1 Note
1 0 0,
PLAT912_ALERT_4_G Missing # of FCF Reflections Above STh/L= 0.600 21 Note
PLAT933_ALERT_2_G Number of HKL-OMIT Records in Embedded .res File 1 Note
1 0 0,
PLAT969_ALERT_5_G The 'Henn et al.' R-Factor-gap value .... 1.66 Note

Predicted wR2: Based on SigI**2 2.25 or SHELX Weight 3.64

---

0 **ALERT level A** = Most likely a serious problem - resolve or explain
0 **ALERT level B** = A potentially serious problem, consider carefully
0 **ALERT level C** = Check. Ensure it is not caused by an omission or oversight
4 **ALERT level G** = General information/check it is not something unexpected

0 ALERT type 1 CIF construction/syntax error, inconsistent or missing data
1 ALERT type 2 Indicator that the structure model may be wrong or deficient
1 ALERT type 3 Indicator that the structure quality may be low
1 ALERT type 4 Improvement, methodology, query or suggestion
1 ALERT type 5 Informative message, check

It is advisable to attempt to resolve as many as possible of the alerts in all categories. Often the minor alerts point to easily fixed oversights, errors and omissions in your CIF or refinement strategy, so attention to these fine details can be worthwhile. In order to resolve some of the more serious problems it may be necessary to carry out additional measurements or structure refinements. However, the purpose of your study may justify the reported deviations and the more serious of these should normally be commented upon in the discussion or experimental section of a paper or in the "special_details" fields of the CIF. checkCIF was carefully designed to identify outliers and unusual parameters, but every test has its limitations and alerts that are not important in a particular case may appear. Conversely, the absence of alerts does not guarantee there are no aspects of the results needing attention. It is up to the individual to critically assess their own results and, if necessary, seek expert advice.

Publication of your CIF in IUCr journals

A basic structural check has been run on your CIF. These basic checks will be run on all CIFs submitted for publication in IUCr journals (*Acta Crystallographica*, *Journal of Applied Crystallography*, *Journal of Synchrotron Radiation*); however, if you intend to submit to *Acta Crystallographica Section C* or *E* or *IUCrData*, you should make sure that full publication checks are run on the final version of your CIF prior to submission.

Publication of your CIF in other journals

Please refer to the *Notes for Authors* of the relevant journal for any special instructions relating to CIF submission.

PLATON version of 06/01/2024; check.def file version of 05/01/2024

Datablock JD_02_01_24_0m - ellipsoid plot

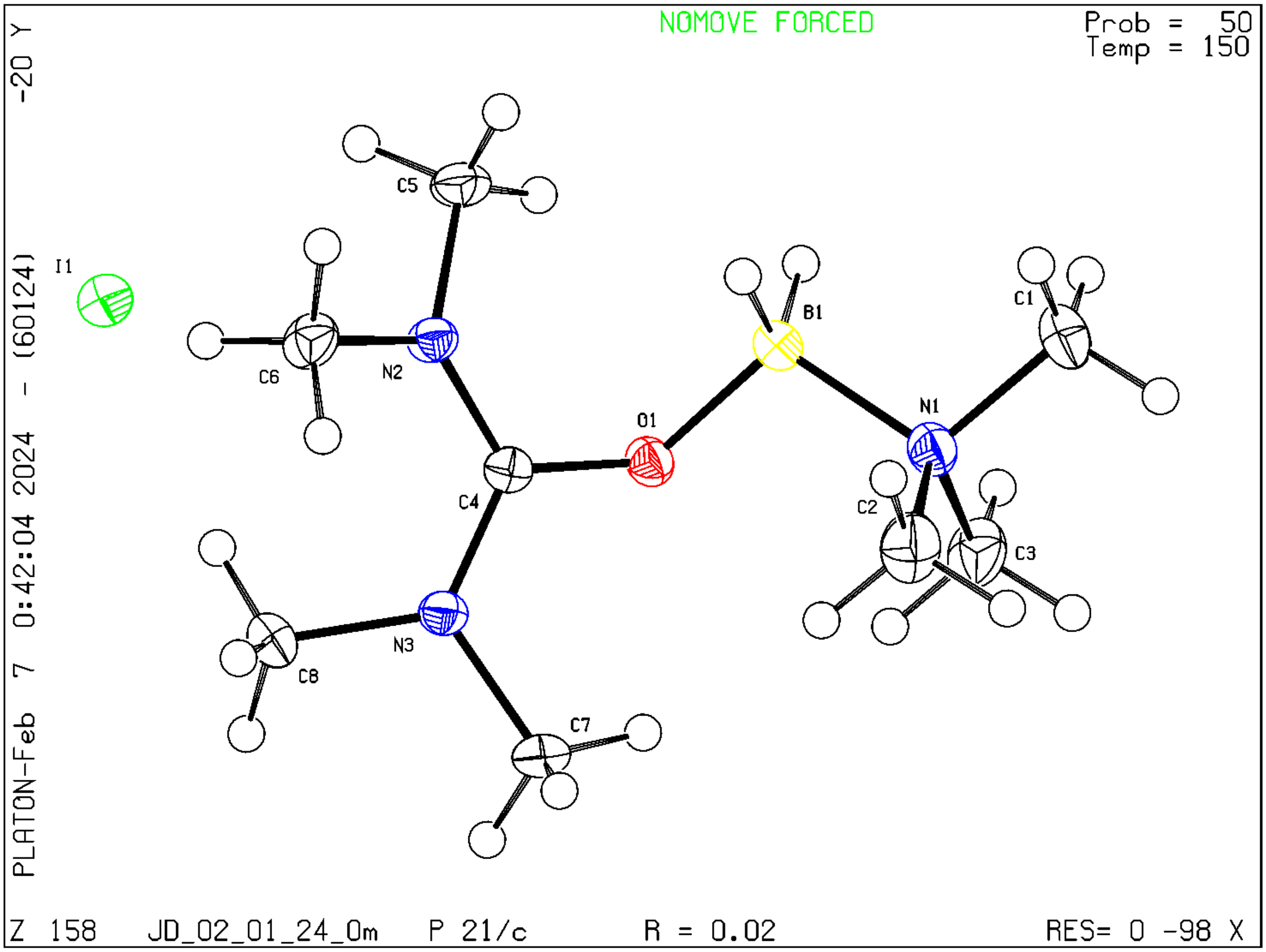

Download CIF editor (publCIF) from the IUCr
Download CIF editor (enCIFer) from the CCDC
Test a new CIF entry

checkCIF (basic structural check) running

Checking for embedded fcf data in CIF ...
Found embedded fcf data in CIF. Extracting fcf data from uploaded CIF, please wait . . . .

checkCIF/PLATON (basic structural check)

Structure factors have been supplied for datablock(s) JD_01_30_24_0m

THIS REPORT IS FOR GUIDANCE ONLY. IF USED AS PART OF A REVIEW PROCEDURE FOR PUBLICATION, IT SHOULD NOT REPLACE THE EXPERTISE OF AN EXPERIENCED CRYSTALLOGRAPHIC REFEREE.

No syntax errors found. CIF dictionary
Please wait while processing .... Interpreting this report

Structure factor report

Datablock: JD_01_30_24_0m

Bond precision:	C-C = 0.0020 A		Wavelength=0.71073
Cell:	a=10.2903(4)	b=8.0230(3)	c=21.9128(8)
	alpha=90	beta=102.169(1)	gamma=90
Temperature:	150 K		

	Calculated	Reported
Volume	1768.45(12)	1768.45(12)
Space group	P 21/c	P 21/c
Hall group	-P 2ybc	-P 2ybc
Moiety formula	C12 H31 B N3 O, I	C12 H31 B N3 O, I
Sum formula	C12 H31 B I N3 O	C12 H31 B I N3 O
Mr	371.11	371.11
Dx,g cm-3	1.394	1.394
Z	4	4
Mu (mm-1)	1.806	1.806
F000	760.0	760.0
F000'	758.30	
h,k,lmax	15,12,33	15,12,33
Nref	6751	6745
Tmin,Tmax	0.465,0.710	0.536,0.747
Tmin'	0.455	

Correction method= # Reported T Limits: Tmin=0.536 Tmax=0.747 AbsCorr = MULTI-SCAN

Data completeness= 0.999 Theta(max)= 33.169

R(reflections)= 0.0229(6220) wR2(reflections)= 0.0487(6745)

S = 1.208 Npar= 193

The following ALERTS were generated. Each ALERT has the format
test-name_ALERT_alert-type_alert-level.
Click on the hyperlinks for more details of the test.

Alert level C

PLAT906_ALERT_3_C Large K Value in the Analysis of Variance ...... 3.356 Check

Alert level G

PLAT002_ALERT_2_G Number of Distance or Angle Restraints on AtSite 11 Note
PLAT003_ALERT_2_G Number of Uiso or Uij Restrained non-H Atoms ... 6 Report
PLAT171_ALERT_4_G The CIF-Embedded .res File Contains EADP Records 1 Report

PLAT175_ALERT_4_G The CIF-Embedded .res File Contains SAME Records 2 Report
PLAT176_ALERT_4_G The CIF-Embedded .res File Contains SADI Records 2 Report
PLAT178_ALERT_4_G The CIF-Embedded .res File Contains SIMU Records 1 Report
PLAT188_ALERT_3_G A Non-default SIMU Restraint Value has been used 0.0100 Report
PLAT301_ALERT_3_G Main Residue Disorder ....(Resd 1) 18% Note
PLAT367_ALERT_2_G Long? C(sp?)-C(sp?) Bond C4 - C5 . 1.52 Ang.
PLAT410_ALERT_2_G Short Intra H...H Contact H4A ..H2D . 2.13 Ang.
x,y,z = 1_555 Check
PLAT790_ALERT_4_G Centre of Gravity not Within Unit Cell: Resd. # 2 Note
I
PLAT860_ALERT_3_G Number of Least-Squares Restraints .... 77 Note
PLAT910_ALERT_3_G Missing # of FCF Reflection(s) Below Theta(Min). 2 Note
1 0 0, 0 0 2,
PLAT912_ALERT_4_G Missing # of FCF Reflections Above STh/L= 0.600 3 Note
PLAT933_ALERT_2_G Number of HKL-OMIT Records in Embedded .res File 1 Note
1 0 0,
PLAT969_ALERT_5_G The 'Henn et al.' R-Factor-gap value .... 2.60 Note
Predicted wR2: Based on SigI**2 1.87 or SHELX Weight 4.09
PLAT978_ALERT_2_G Number C-C Bonds with Positive Residual Density. 3 Info

---

0 **ALERT level A** = Most likely a serious problem - resolve or explain
0 **ALERT level B** = A potentially serious problem, consider carefully
1 **ALERT level C** = Check. Ensure it is not caused by an omission or oversight
17 **ALERT level G** = General information/check it is not something unexpected

0 ALERT type 1 CIF construction/syntax error, inconsistent or missing data
6 ALERT type 2 Indicator that the structure model may be wrong or deficient
5 ALERT type 3 Indicator that the structure quality may be low
6 ALERT type 4 Improvement, methodology, query or suggestion
1 ALERT type 5 Informative message, check

It is advisable to attempt to resolve as many as possible of the alerts in all categories. Often the minor alerts point to easily fixed oversights, errors and omissions in your CIF or refinement strategy, so attention to these fine details can be worthwhile. In order to resolve some of the more serious problems it may be necessary to carry out additional measurements or structure refinements. However, the purpose of your study may justify the reported deviations and the more serious of these should normally be commented upon in the discussion or experimental section of a paper or in the "special_details" fields of the CIF. checkCIF was carefully designed to identify outliers and unusual parameters, but every test has its limitations and alerts that are not important in a particular case may appear. Conversely, the absence of alerts does not guarantee there are no aspects of the results needing attention. It is up to the individual to critically assess their own results and, if necessary, seek expert advice.

Publication of your CIF in IUCr journals

A basic structural check has been run on your CIF. These basic checks will be run on all CIFs submitted for publication in IUCr journals (*Acta Crystallographica*, *Journal of Applied Crystallography*, *Journal of Synchrotron Radiation*); however, if you intend to submit to *Acta Crystallographica Section C* or *E* or *IUCrData*, you should make sure that full publication checks are run on the final version of your CIF prior to submission.

Publication of your CIF in other journals

Please refer to the *Notes for Authors* of the relevant journal for any special instructions relating to CIF submission.

PLATON version of 06/01/2024; check.def file version of 05/01/2024

Datablock JD_01_30_24_0m - ellipsoid plot

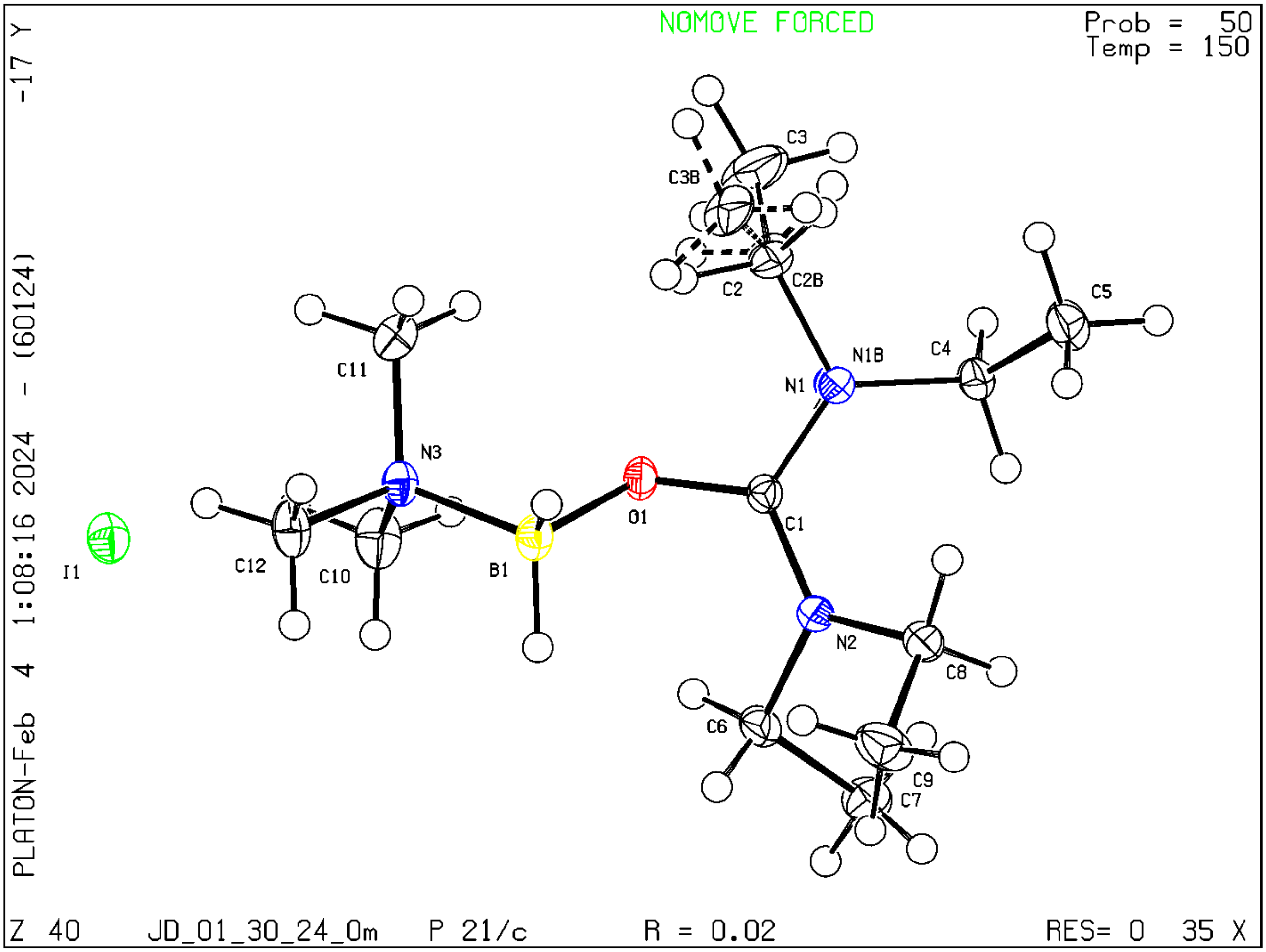

Download CIF editor (publCIF) from the IUCr
Download CIF editor (enCIFer) from the CCDC
Test a new CIF entry

checkCIF (basic structural check) running

Checking for embedded fcf data in CIF ...
Found embedded fcf data in CIF. Extracting fcf data from uploaded CIF, please wait . . .

checkCIF/PLATON (basic structural check)

Structure factors have been supplied for datablock(s) JD_01_05_24_0m

THIS REPORT IS FOR GUIDANCE ONLY. IF USED AS PART OF A REVIEW PROCEDURE FOR PUBLICATION, IT SHOULD NOT REPLACE THE EXPERTISE OF AN EXPERIENCED CRYSTALLOGRAPHIC REFEREE.

No syntax errors found. CIF dictionary
Please wait while processing .... Interpreting this report

Structure factor report

Datablock: JD_01_05_24_0m

Bond precision:	C-C = 0.0050 A		Wavelength=0.71073
Cell:	a=9.6367(6)	b=12.7626(7)	c=11.3363(6)
	alpha=90	beta=107.192(3)	gamma=90
Temperature:	150 K		

	Calculated	Reported
Volume	1331.95(13)	1331.95(13)
Space group	P 21/c	P 21/c
Hall group	-P 2ybc	-P 2ybc
Moiety formula	C8 H21 B N3 O, I	C8 H21 B N3 O, I
Sum formula	C8 H21 B I N3 O	C8 H21 B I N3 O
Mr	312.99	312.99
Dx,g cm-3	1.561	1.561
Z	4	4
Mu (mm-1)	2.382	2.382
F000	624.0	624.0
F000'	622.27	
h,k,lmax	14,19,17	14,18,17
Nref	5086	4954
Tmin,Tmax	0.281,0.909	0.502,0.747
Tmin'	0.259	

Correction method= # Reported T Limits: Tmin=0.502 Tmax=0.747 AbsCorr = MULTI-SCAN

Data completeness= 0.974 Theta(max)= 33.162

R(reflections)= 0.0362(3637) wR2(reflections)= 0.0962(4954)

S = 1.018 Npar= 134

The following ALERTS were generated. Each ALERT has the format
test-name_ALERT_alert-type_alert-level.
Click on the hyperlinks for more details of the test.

Alert level G

PLAT910_ALERT_3_G Missing # of FCF Reflection(s) Below Theta(Min). 1 Note
1 0 0,
PLAT912_ALERT_4_G Missing # of FCF Reflections Above STh/L= 0.600 107 Note
PLAT933_ALERT_2_G Number of HKL-OMIT Records in Embedded .res File 1 Note
1 0 0,
PLAT969_ALERT_5_G The 'Henn et al.' R-Factor-gap value .... 1.48 Note

Predicted wR2: Based on SigI**2 6.48 or SHELX Weight 9.58
PLAT978_ALERT_2_G Number C-C Bonds with Positive Residual Density. 0 Info

0 **ALERT level A** = Most likely a serious problem - resolve or explain
0 **ALERT level B** = A potentially serious problem, consider carefully
0 **ALERT level C** = Check. Ensure it is not caused by an omission or oversight
5 **ALERT level G** = General information/check it is not something unexpected

0 ALERT type 1 CIF construction/syntax error, inconsistent or missing data
2 ALERT type 2 Indicator that the structure model may be wrong or deficient
1 ALERT type 3 Indicator that the structure quality may be low
1 ALERT type 4 Improvement, methodology, query or suggestion
1 ALERT type 5 Informative message, check

It is advisable to attempt to resolve as many as possible of the alerts in all categories. Often the minor alerts point to easily fixed oversights, errors and omissions in your CIF or refinement strategy, so attention to these fine details can be worthwhile. In order to resolve some of the more serious problems it may be necessary to carry out additional measurements or structure refinements. However, the purpose of your study may justify the reported deviations and the more serious of these should normally be commented upon in the discussion or experimental section of a paper or in the "special_details" fields of the CIF. checkCIF was carefully designed to identify outliers and unusual parameters, but every test has its limitations and alerts that are not important in a particular case may appear. Conversely, the absence of alerts does not guarantee there are no aspects of the results needing attention. It is up to the individual to critically assess their own results and, if necessary, seek expert advice.

Publication of your CIF in IUCr journals

A basic structural check has been run on your CIF. These basic checks will be run on all CIFs submitted for publication in IUCr journals (*Acta Crystallographica*, *Journal of Applied Crystallography*, *Journal of Synchrotron Radiation*); however, if you intend to submit to *Acta Crystallographica Section C* or *E* or *IUCrData*, you should make sure that full publication checks are run on the final version of your CIF prior to submission.

Publication of your CIF in other journals

Please refer to the *Notes for Authors* of the relevant journal for any special instructions relating to CIF submission.

PLATON version of 13/12/2023; check.def file version of 13/12/2023

Datablock JD_01_05_24_0m - ellipsoid plot

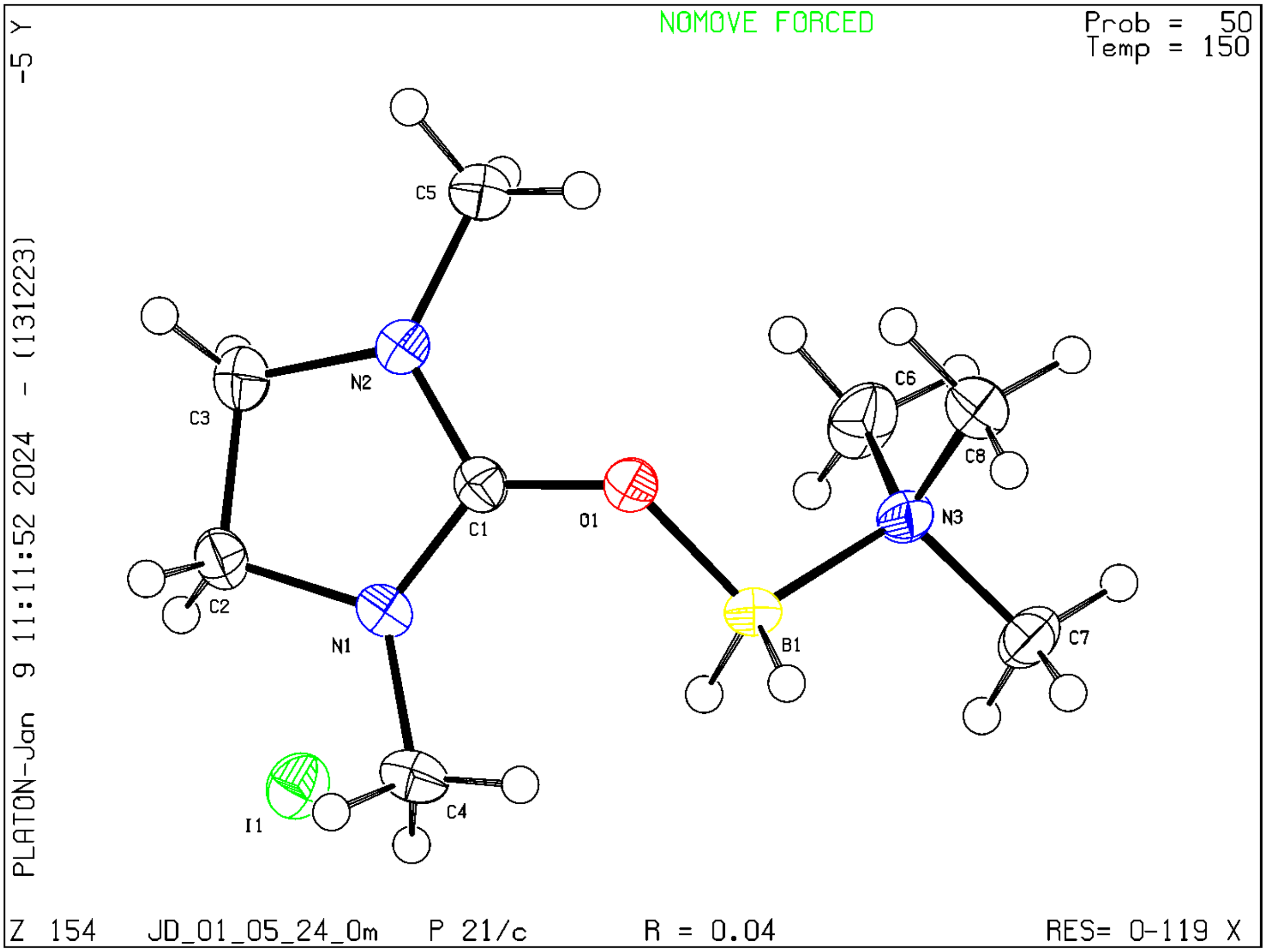

Download CIF editor (publCIF) from the IUCr
Download CIF editor (enCIFer) from the CCDC
Test a new CIF entry

checkCIF (basic structural check) running

Checking for embedded fcf data in CIF ...
Found embedded fcf data in CIF. Extracting fcf data from uploaded CIF, please wait . . . . . .

checkCIF/PLATON (basic structural check)

Structure factors have been supplied for datablock(s) JD_01_10_24_A_0m

THIS REPORT IS FOR GUIDANCE ONLY. IF USED AS PART OF A REVIEW PROCEDURE FOR PUBLICATION, IT SHOULD NOT REPLACE THE EXPERTISE OF AN EXPERIENCED CRYSTALLOGRAPHIC REFEREE.

No syntax errors found. CIF dictionary
Please wait while processing .... Interpreting this report

Structure factor report

Datablock: JD_01_10_24_A_0m

Bond precision:	C-C = 0.0020 A		Wavelength=0.71073
Cell:	a=19.249(12)	b=16.089(3)	c=19.492(6)
	alpha=90	beta=90	gamma=90
Temperature:	150 K		

	Calculated	Reported
Volume	6037(4)	6036(4)
Space group	P b c a	P b c a
Hall group	-P 2ac 2ab	-P 2ac 2ab
Moiety formula	C24 H20 B, C9 H23 B N3 O	?
Sum formula	C33 H43 B2 N3 O	C33 H43 B2 N3 O
Mr	519.32	519.32
Dx,g cm-3	1.143	1.143
Z	8	8
Mu (mm-1)	0.068	0.068
F000	2240.0	2240.0
F000'	2240.74	
h,k,lmax	25,21,25	25,21,25
Nref	7490	7484
Tmin,Tmax	0.978,0.990	0.671,0.746
Tmin'	0.978	

Correction method= # Reported T Limits: Tmin=0.671 Tmax=0.746 AbsCorr = MULTI-SCAN

Data completeness= 0.999 Theta(max)= 28.278

R(reflections)= 0.0434(5831) wR2(reflections)= 0.1203(7484)

S = 1.038 Npar= 390

The following ALERTS were generated. Each ALERT has the format
test-name_ALERT_alert-type_alert-level.
Click on the hyperlinks for more details of the test.

Alert level C

PLAT905_ALERT_3_C Negative K value in the Analysis of Variance ... -0.217 Report
PLAT911_ALERT_3_C Missing FCF Refl Between Thmin & STh/L= 0.600 2 Report
2 1 0, 4 3 2,

Alert level G

PLAT002_ALERT_2_G Number of Distance or Angle Restraints on AtSite 8 Note

```
PLAT003_ALERT_2_G Number of Uiso or Uij Restrained non-H Atoms ...          8 Report
PLAT168_ALERT_4_G The CIF-Embedded .res File Contains EXYZ Records          1 Report
PLAT171_ALERT_4_G The CIF-Embedded .res File Contains EADP Records          1 Report
PLAT175_ALERT_4_G The CIF-Embedded .res File Contains SAME Records          1 Report
PLAT178_ALERT_4_G The CIF-Embedded .res File Contains SIMU Records          1 Report
PLAT188_ALERT_3_G A Non-default SIMU Restraint Value has been used     0.0100 Report
PLAT302_ALERT_4_G Anion/Solvent/Minor-Residue Disorder (Resd    2)        29% Note
PLAT412_ALERT_2_G Short Intra XH3 .. XHn     H1B      ..H9E      .       1.65 Ang.
                                          x,y,z  =      1_555 Check
PLAT413_ALERT_2_G Short Inter XH3 .. XHn     H8A      ..H17      .       2.12 Ang.
                                 1-x,-1/2+y,3/2-z  =      3_646 Check
PLAT860_ALERT_3_G Number of Least-Squares Restraints ............        114 Note
PLAT910_ALERT_3_G Missing # of FCF Reflection(s) Below Theta(Min).          3 Note
         2  0  0,   1  1  1,   0  0  2,
PLAT912_ALERT_4_G Missing # of FCF Reflections Above STh/L=  0.600          1 Note
PLAT933_ALERT_2_G Number of HKL-OMIT Records in Embedded .res File          3 Note
         0  0  2,   4  3  2,   1  1  1,
PLAT969_ALERT_5_G The 'Henn et al.' R-Factor-gap value ..........       4.80 Note
           Predicted wR2: Based on SigI**2  2.51 or SHELX Weight 11.90
PLAT978_ALERT_2_G Number C-C Bonds with Positive Residual Density.         10 Info
```

---

0 **ALERT level A** = Most likely a serious problem - resolve or explain
0 **ALERT level B** = A potentially serious problem, consider carefully
2 **ALERT level C** = Check. Ensure it is not caused by an omission or oversight
16 **ALERT level G** = General information/check it is not something unexpected

0 ALERT type 1 CIF construction/syntax error, inconsistent or missing data
6 ALERT type 2 Indicator that the structure model may be wrong or deficient
5 ALERT type 3 Indicator that the structure quality may be low
6 ALERT type 4 Improvement, methodology, query or suggestion
1 ALERT type 5 Informative message, check

It is advisable to attempt to resolve as many as possible of the alerts in all categories. Often the minor alerts point to easily fixed oversights, errors and omissions in your CIF or refinement strategy, so attention to these fine details can be worthwhile. In order to resolve some of the more serious problems it may be necessary to carry out additional measurements or structure refinements. However, the purpose of your study may justify the reported deviations and the more serious of these should normally be commented upon in the discussion or experimental section of a paper or in the "special_details" fields of the CIF. checkCIF was carefully designed to identify outliers and unusual parameters, but every test has its limitations and alerts that are not important in a particular case may appear. Conversely, the absence of alerts does not guarantee there are no aspects of the results needing attention. It is up to the individual to critically assess their own results and, if necessary, seek expert advice.

Publication of your CIF in IUCr journals

A basic structural check has been run on your CIF. These basic checks will be run on all CIFs submitted for publication in IUCr journals (*Acta Crystallographica*, *Journal of Applied Crystallography*, *Journal of Synchrotron Radiation*); however, if you intend to submit to *Acta Crystallographica Section C* or *E* or *IUCrData*, you should make sure that full publication checks are run on the final version of your CIF prior to submission.

Publication of your CIF in other journals

Please refer to the *Notes for Authors* of the relevant journal for any special instructions relating to CIF submission.

PLATON version of 13/12/2023; check.def file version of 13/12/2023

Datablock JD_01_10_24_A_0m - ellipsoid plot

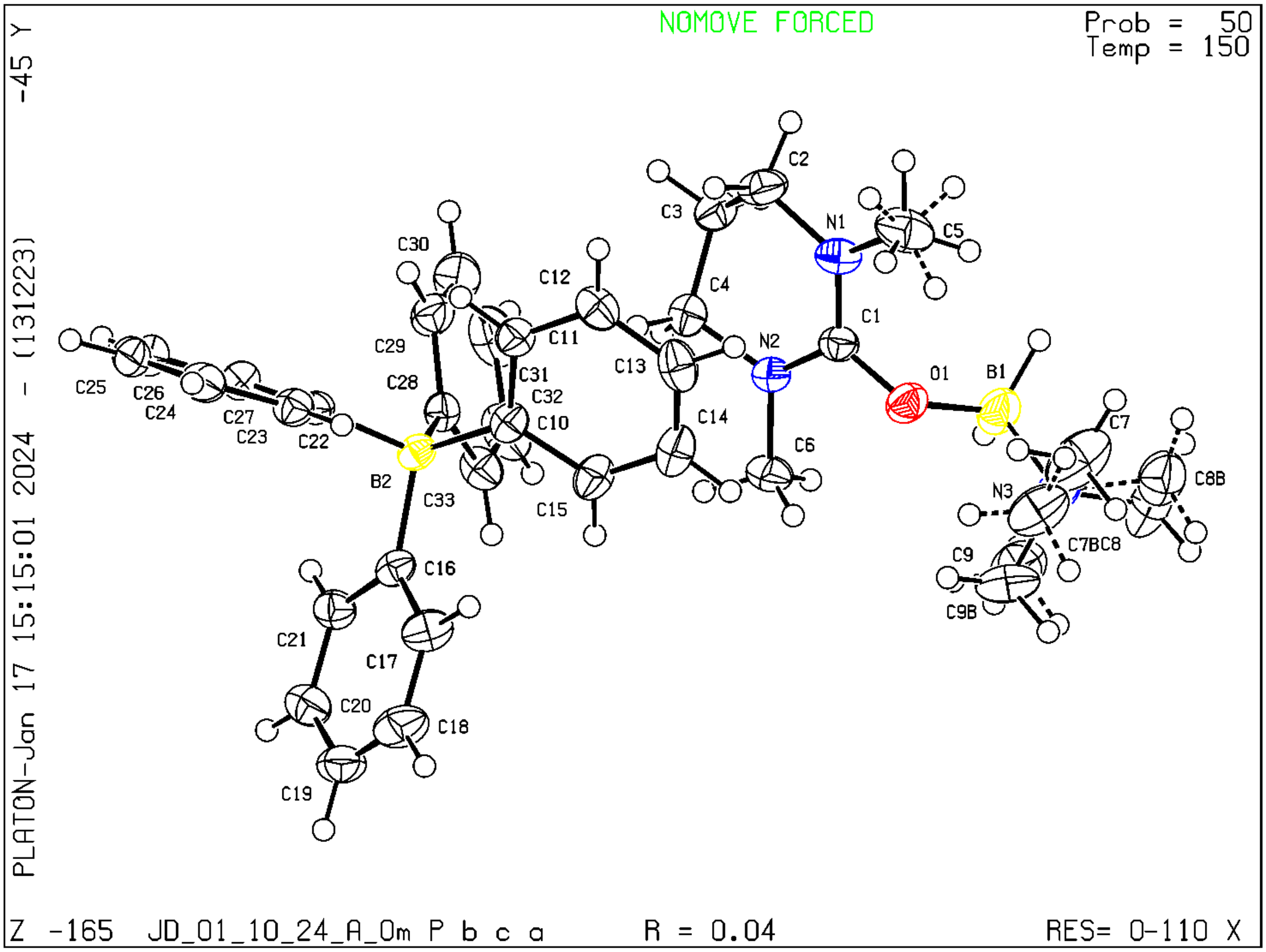

Download CIF editor (publCIF) from the IUCr
Download CIF editor (enCIFer) from the CCDC
Test a new CIF entry

checkCIF (basic structural check) running

Checking for embedded fcf data in CIF ...
Found embedded fcf data in CIF. Extracting fcf data from uploaded CIF, please wait . . . .

checkCIF/PLATON (basic structural check)

Structure factors have been supplied for datablock(s) JD_12_18_23_0m

THIS REPORT IS FOR GUIDANCE ONLY. IF USED AS PART OF A REVIEW PROCEDURE FOR PUBLICATION, IT SHOULD NOT REPLACE THE EXPERTISE OF AN EXPERIENCED CRYSTALLOGRAPHIC REFEREE.

No syntax errors found. CIF dictionary
Please wait while processing .... Interpreting this report

Structure factor report

Datablock: JD_12_18_23_0m

Bond precision:	C-C = 0.0012 A		Wavelength=0.71073
Cell:	a=26.8272(11)	b=10.6360(5)	c=9.7782(4)
	alpha=90	beta=90	gamma=90
Temperature:	150 K		

	Calculated	Reported
Volume	2790.1(2)	2790.1(2)
Space group	P n m a	P n m a
Hall group	-P 2ac 2n	-P 2ac 2n
Moiety formula	C24 H20 B, C7 H20 B N2 O	C24 H20 B, C7 H20 B N2 O
Sum formula	C31 H40 B2 N2 O	C31 H40 B2 N2 O
Mr	478.27	478.27
Dx,g cm-3	1.139	1.139
Z	4	4
Mu (mm-1)	0.067	0.067
F000	1032.0	1032.0
F000'	1032.35	
h,k,lmax	41,16,15	41,16,14
Nref	5577	5563
Tmin,Tmax	0.966,0.974	0.679,0.747
Tmin'	0.966	

Correction method= # Reported T Limits: Tmin=0.679 Tmax=0.747 AbsCorr = MULTI-SCAN

Data completeness= 0.997 Theta(max)= 33.178

R(reflections)= 0.0440(4327) wR2(reflections)= 0.1342(5563)

S = 1.050 Npar= 210

The following ALERTS were generated. Each ALERT has the format
test-name_ALERT_alert-type_alert-level.
Click on the hyperlinks for more details of the test.

Alert level C

PLAT218_ALERT_3_C Constrained U(ij) Components(s) for N2 . 2 Check
PLAT250_ALERT_2_C Large U3/U1 Ratio for <U(i,j)> Tensor(Resd 2) 2.1 Note
PLAT906_ALERT_3_C Large K Value in the Analysis of Variance ...... 3.768 Check

Alert level G

FORMU01_ALERT_2_G There is a discrepancy between the atom counts in the

_chemical_formula_sum and the formula from the _atom_site* data.
Atom count from _chemical_formula_sum:C31 H40 B2 N2 O1
Atom count from the _atom_site data: C31 H40 B2 N1.5 O1
CELLZ01_ALERT_1_G Difference between formula and atom_site contents detected.
CELLZ01_ALERT_1_G ALERT: Large difference may be due to a
symmetry error - see SYMMG tests
From the CIF: _cell_formula_units_Z 4
From the CIF: _chemical_formula_sum C31 H40 B2 N2 O
TEST: Compare cell contents of formula and atom_site data

atom	Z*formula	cif sites	diff
C	124.00	124.00	0.00
H	160.00	160.00	0.00
B	8.00	8.00	0.00
N	8.00	6.00	2.00
O	4.00	4.00	0.00

PLAT002_ALERT_2_G Number of Distance or Angle Restraints on AtSite 4 Note
PLAT003_ALERT_2_G Number of Uiso or Uij Restrained non-H Atoms ... 6 Report
PLAT176_ALERT_4_G The CIF-Embedded .res File Contains SADI Records 2 Report
PLAT178_ALERT_4_G The CIF-Embedded .res File Contains SIMU Records 1 Report
PLAT188_ALERT_3_G A Non-default SIMU Restraint Value has been used 0.0100 Report
PLAT191_ALERT_3_G A Non-default SADI Restraint Value has been used 0.0100 Report
PLAT300_ALERT_4_G Atom Site Occupancy of O1 Constrained at 0.5 Check
And 25 other PLAT300 Alerts
More ...
PLAT302_ALERT_4_G Anion/Solvent/Minor-Residue Disorder (Resd 2) 55% Note
PLAT779_ALERT_4_G Suspect or Irrelevant (Bond) Angle(s) in CIF ... 42.50 Deg.
H3C -C3 -H3A 1_555 1_555 7_565 ...... # 68 Check
And 5 other PLAT779 Alerts
More ...
PLAT789_ALERT_4_G Atoms with Negative _atom_site_disorder_group # 17 Check
PLAT822_ALERT_4_G CIF-embedded .res Contains Negative PART Numbers 1 Check
PLAT860_ALERT_3_G Number of Least-Squares Restraints .... 36 Note
PLAT910_ALERT_3_G Missing # of FCF Reflection(s) Below Theta(Min). 1 Note
2 0 0,
PLAT912_ALERT_4_G Missing # of FCF Reflections Above STh/L= 0.600 6 Note
PLAT969_ALERT_5_G The 'Henn et al.' R-Factor-gap value .... 6.68 Note
Predicted wR2: Based on SigI**2 2.01 or SHELX Weight 13.03
PLAT978_ALERT_2_G Number C-C Bonds with Positive Residual Density. 13 Info

---

0 **ALERT level A** = Most likely a serious problem - resolve or explain
0 **ALERT level B** = A potentially serious problem, consider carefully
3 **ALERT level C** = Check. Ensure it is not caused by an omission or oversight
49 **ALERT level G** = General information/check it is not something unexpected

2 ALERT type 1 CIF construction/syntax error, inconsistent or missing data
5 ALERT type 2 Indicator that the structure model may be wrong or deficient
6 ALERT type 3 Indicator that the structure quality may be low
38 ALERT type 4 Improvement, methodology, query or suggestion
1 ALERT type 5 Informative message, check

It is advisable to attempt to resolve as many as possible of the alerts in all categories. Often the minor alerts point to easily fixed oversights, errors and omissions in your CIF or refinement strategy, so attention to these fine details can be worthwhile. In order to resolve some of the more serious problems it may be necessary to carry out additional measurements or structure refinements. However, the purpose of your study may justify the reported deviations and the more serious of these should normally be commented upon in the discussion or experimental section of a paper or in the "special_details" fields of the CIF. checkCIF was carefully designed to identify outliers and unusual parameters, but every test has its limitations and alerts that are not important in a particular case may appear. Conversely, the absence of alerts does not guarantee there are no aspects of the results needing attention. It is up to the individual to critically assess their own results and, if necessary, seek expert advice.

Publication of your CIF in IUCr journals

A basic structural check has been run on your CIF. These basic checks will be run on all CIFs submitted for publication in IUCr journals (*Acta Crystallographica*, *Journal of Applied Crystallography*, *Journal of Synchrotron Radiation*); however, if you intend to submit to *Acta Crystallographica Section C* or *E* or

IUCrData, you should make sure that full publication checks are run on the final version of your CIF prior to submission.

Publication of your CIF in other journals

Please refer to the *Notes for Authors* of the relevant journal for any special instructions relating to CIF submission.

PLATON version of 13/12/2023; check.def file version of 13/12/2023

Datablock JD_12_18_23_0m - ellipsoid plot

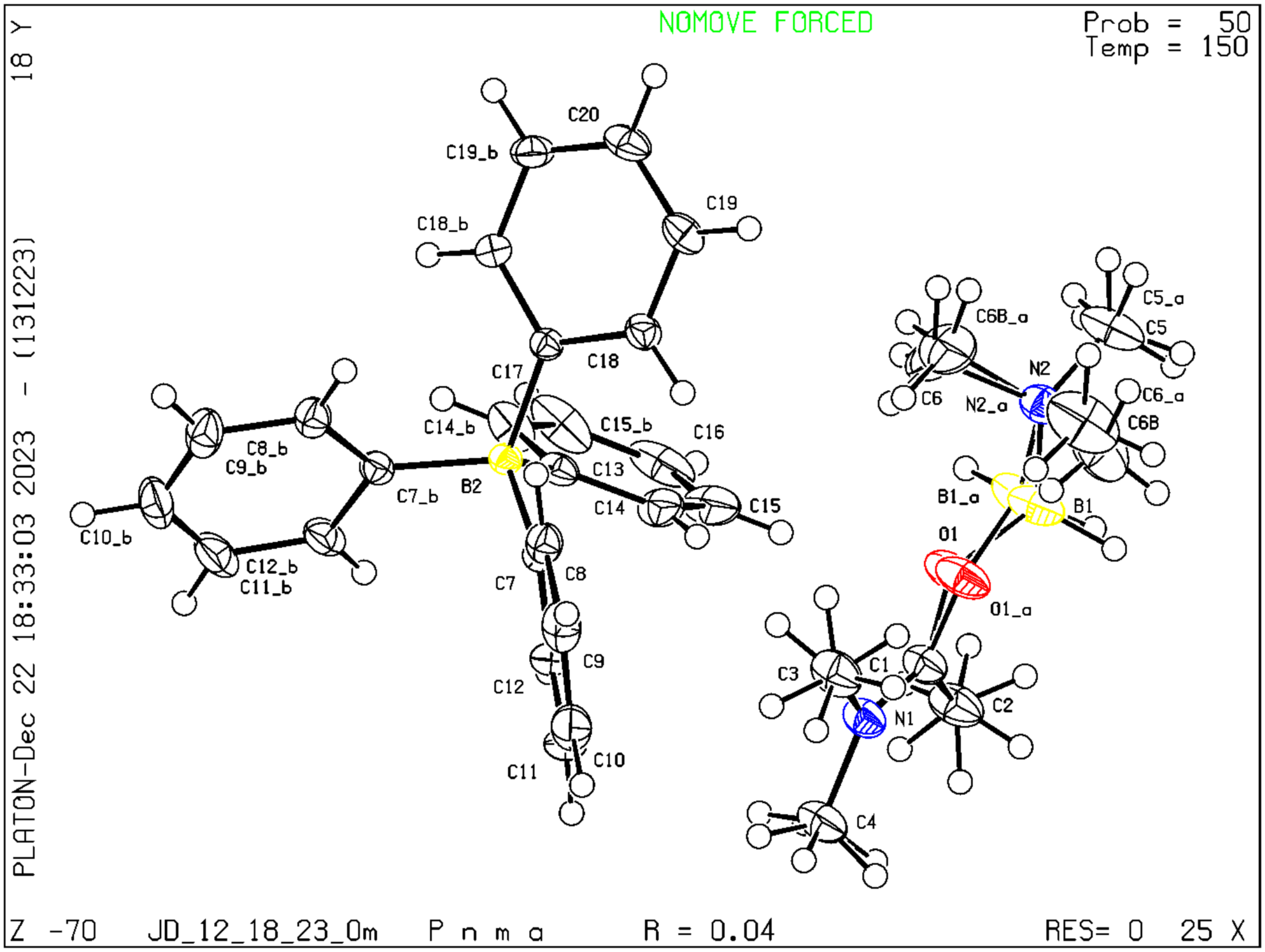

Download CIF editor (publCIF) from the IUCr
Download CIF editor (enCIFer) from the CCDC
Test a new CIF entry

checkCIF (basic structural check) running

Checking for embedded fcf data in CIF ...
Found embedded fcf data in CIF. Extracting fcf data from uploaded CIF, please wait . . . . . .

checkCIF/PLATON (basic structural check)

Structure factors have been supplied for datablock(s) JD_12_13_23_0m

THIS REPORT IS FOR GUIDANCE ONLY. IF USED AS PART OF A REVIEW PROCEDURE FOR PUBLICATION, IT SHOULD NOT REPLACE THE EXPERTISE OF AN EXPERIENCED CRYSTALLOGRAPHIC REFEREE.

No syntax errors found. CIF dictionary
Please wait while processing .... Interpreting this report

Structure factor report

Datablock: JD_12_13_23_0m

Bond precision:	C-C = 0.0015 A		Wavelength=0.71073
Cell:	a=11.1371(6)	b=15.7447(8)	c=17.0090(8)
	alpha=90	beta=104.339(2)	gamma=90
Temperature:	150 K		

	Calculated	Reported
Volume	2889.6(3)	2889.6(3)
Space group	P 21/n	P 21/n
Hall group	-P 2yn	-P 2yn
Moiety formula	C24 H20 B, C8 H20 B N2 O	C24 H20 B, C8 H20 B N2 O
Sum formula	C32 H40 B2 N2 O	C32 H40 B2 N2 O
Mr	490.28	490.28
Dx,g cm-3	1.127	1.127
Z	4	4
Mu (mm-1)	0.066	0.066
F000	1056.0	1056.0
F000'	1056.36	
h,k,lmax	17,24,26	17,24,26
Nref	11046	11039
Tmin,Tmax	0.971,0.980	0.700,0.747
Tmin'	0.971	

Correction method= # Reported T Limits: Tmin=0.700 Tmax=0.747 AbsCorr = MULTI-SCAN

Data completeness= 0.999 Theta(max)= 33.170

R(reflections)= 0.0485(8333) wR2(reflections)= 0.1449(11039)

S = 1.018 Npar= 339

The following ALERTS were generated. Each ALERT has the format
test-name_ALERT_alert-type_alert-level.
Click on the hyperlinks for more details of the test.

Alert level C

PLAT905_ALERT_3_C Negative K value in the Analysis of Variance ... -0.119 Report

Alert level G

PLAT910_ALERT_3_G Missing # of FCF Reflection(s) Below Theta(Min). 2 Note
-1 0 1, 0 1 1,
PLAT912_ALERT_4_G Missing # of FCF Reflections Above STh/L= 0.600 5 Note

PLAT978_ALERT_2_G Number C-C Bonds with Positive Residual Density. 17 Info

0 **ALERT level A** = Most likely a serious problem - resolve or explain
0 **ALERT level B** = A potentially serious problem, consider carefully
1 **ALERT level C** = Check. Ensure it is not caused by an omission or oversight
3 **ALERT level G** = General information/check it is not something unexpected

0 ALERT type 1 CIF construction/syntax error, inconsistent or missing data
1 ALERT type 2 Indicator that the structure model may be wrong or deficient
2 ALERT type 3 Indicator that the structure quality may be low
1 ALERT type 4 Improvement, methodology, query or suggestion
0 ALERT type 5 Informative message, check

It is advisable to attempt to resolve as many as possible of the alerts in all categories. Often the minor alerts point to easily fixed oversights, errors and omissions in your CIF or refinement strategy, so attention to these fine details can be worthwhile. In order to resolve some of the more serious problems it may be necessary to carry out additional measurements or structure refinements. However, the purpose of your study may justify the reported deviations and the more serious of these should normally be commented upon in the discussion or experimental section of a paper or in the "special_details" fields of the CIF. checkCIF was carefully designed to identify outliers and unusual parameters, but every test has its limitations and alerts that are not important in a particular case may appear. Conversely, the absence of alerts does not guarantee there are no aspects of the results needing attention. It is up to the individual to critically assess their own results and, if necessary, seek expert advice.

Publication of your CIF in IUCr journals

A basic structural check has been run on your CIF. These basic checks will be run on all CIFs submitted for publication in IUCr journals (*Acta Crystallographica*, *Journal of Applied Crystallography*, *Journal of Synchrotron Radiation*); however, if you intend to submit to *Acta Crystallographica Section C* or *E* or *IUCrData*, you should make sure that full publication checks are run on the final version of your CIF prior to submission.

Publication of your CIF in other journals

Please refer to the *Notes for Authors* of the relevant journal for any special instructions relating to CIF submission.

PLATON version of 29/11/2023; check.def file version of 14/09/2023

Datablock JD_12_13_23_0m - ellipsoid plot

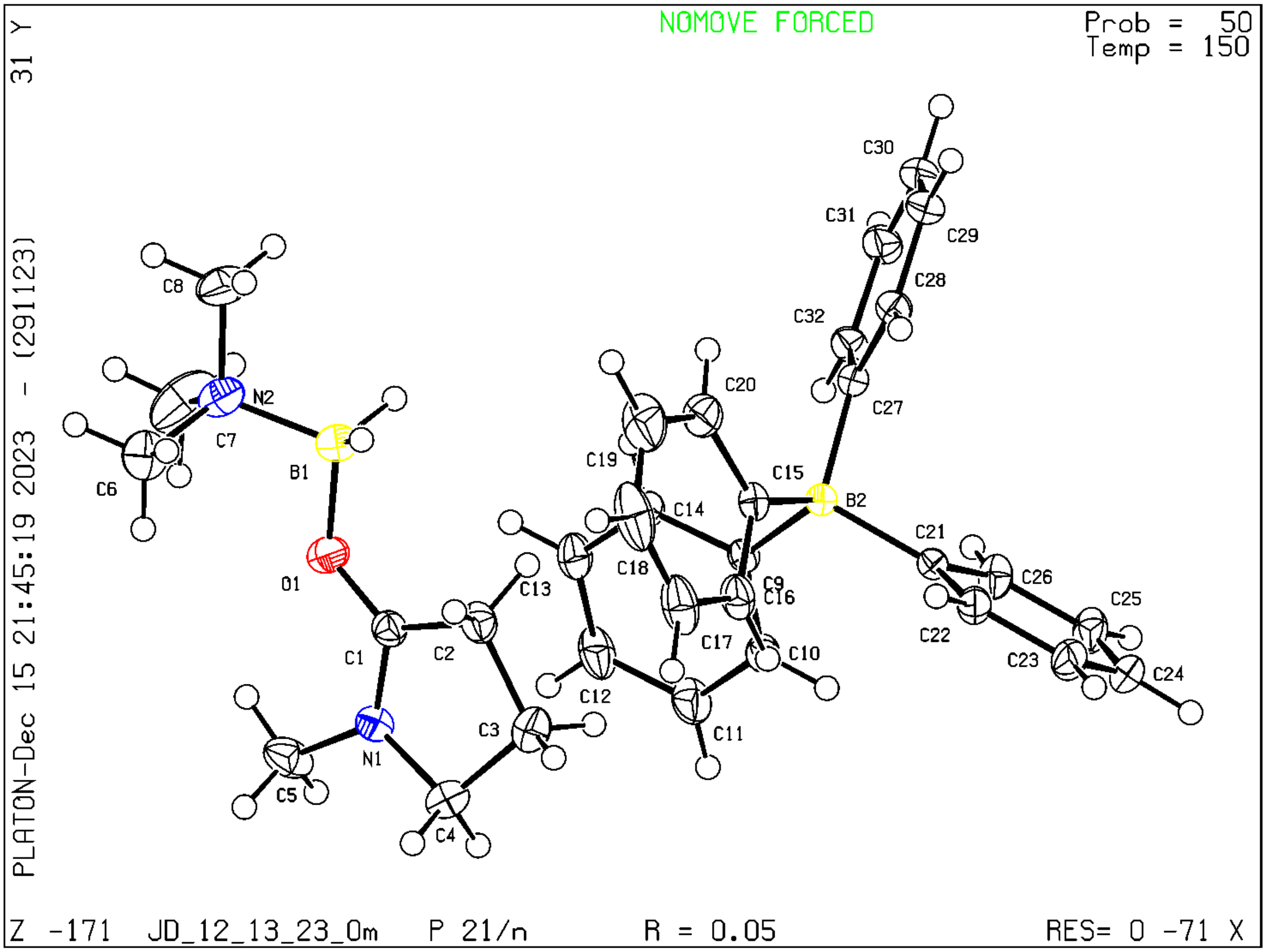

Download CIF editor (publCIF) from the IUCr
Download CIF editor (enCIFer) from the CCDC
Test a new CIF entry

checkCIF (basic structural check) running

Checking for embedded fcf data in CIF ...
Found embedded fcf data in CIF. Extracting fcf data from uploaded CIF, please wait . . . .

checkCIF/PLATON (basic structural check)

Structure factors have been supplied for datablock(s) JD_04_08_24_0m

THIS REPORT IS FOR GUIDANCE ONLY. IF USED AS PART OF A REVIEW PROCEDURE FOR PUBLICATION, IT SHOULD NOT REPLACE THE EXPERTISE OF AN EXPERIENCED CRYSTALLOGRAPHIC REFEREE.

No syntax errors found. CIF dictionary
Please wait while processing .... Interpreting this report

Structure factor report

Datablock: JD_04_08_24_0m

Bond precision:	C-C = 0.0017 A		Wavelength=0.71073
Cell:	a=17.2691(7)	b=11.3208(6)	c=16.7577(7)
	alpha=90	beta=112.730(2)	gamma=90
Temperature:	150 K		

	Calculated	Reported
Volume	3021.7(2)	3021.7(2)
Space group	P 21/c	P 21/c
Hall group	-P 2ybc	-P 2ybc
Moiety formula	C24 H20 B, C9 H19 B N3 O2	C24 H20 B, C9 H19 B N3 O2
Sum formula	C33 H39 B2 N3 O2	C33 H39 B2 N3 O2
Mr	531.29	531.29
Dx,g cm-3	1.168	1.168
Z	4	4
Mu (mm-1)	0.072	0.072
F000	1136.0	1136.0
F000'	1136.41	
h,k,lmax	26,17,25	26,17,25
Nref	11559	11361
Tmin,Tmax	0.968,0.977	0.682,0.747
Tmin'	0.968	

Correction method= # Reported T Limits: Tmin=0.682 Tmax=0.747 AbsCorr = MULTI-SCAN

Data completeness= 0.983   Theta(max)= 33.176

R(reflections)= 0.0497(7882)   wR2(reflections)= 0.1401(11361)

S = 1.022   Npar= 367

The following ALERTS were generated. Each ALERT has the format
test-name_ALERT_alert-type_alert-level.
Click on the hyperlinks for more details of the test.

Alert level C

PLAT911_ALERT_3_C Missing FCF Refl Between Thmin & STh/L= 0.600 2 Report
-1 0 2, -7 0 8,

Alert level G

PLAT910_ALERT_3_G Missing # of FCF Reflection(s) Below Theta(Min). 3 Note
1 0 0, 1 1 0, 0 1 1,

PLAT912_ALERT_4_G Missing # of FCF Reflections Above STh/L= 0.600 193 Note
PLAT933_ALERT_2_G Number of HKL-OMIT Records in Embedded .res File 1 Note
0 1 1,
PLAT969_ALERT_5_G The 'Henn et al.' R-Factor-gap value .... 3.59 Note
Predicted wR2: Based on SigI**2 3.90 or SHELX Weight 13.93
PLAT978_ALERT_2_G Number C-C Bonds with Positive Residual Density. 13 Info

---

0 **ALERT level A** = Most likely a serious problem - resolve or explain
0 **ALERT level B** = A potentially serious problem, consider carefully
1 **ALERT level C** = Check. Ensure it is not caused by an omission or oversight
5 **ALERT level G** = General information/check it is not something unexpected

0 ALERT type 1 CIF construction/syntax error, inconsistent or missing data
2 ALERT type 2 Indicator that the structure model may be wrong or deficient
2 ALERT type 3 Indicator that the structure quality may be low
1 ALERT type 4 Improvement, methodology, query or suggestion
1 ALERT type 5 Informative message, check

It is advisable to attempt to resolve as many as possible of the alerts in all categories. Often the minor alerts point to easily fixed oversights, errors and omissions in your CIF or refinement strategy, so attention to these fine details can be worthwhile. In order to resolve some of the more serious problems it may be necessary to carry out additional measurements or structure refinements. However, the purpose of your study may justify the reported deviations and the more serious of these should normally be commented upon in the discussion or experimental section of a paper or in the "special_details" fields of the CIF. checkCIF was carefully designed to identify outliers and unusual parameters, but every test has its limitations and alerts that are not important in a particular case may appear. Conversely, the absence of alerts does not guarantee there are no aspects of the results needing attention. It is up to the individual to critically assess their own results and, if necessary, seek expert advice.

Publication of your CIF in IUCr journals

A basic structural check has been run on your CIF. These basic checks will be run on all CIFs submitted for publication in IUCr journals (*Acta Crystallographica*, *Journal of Applied Crystallography*, *Journal of Synchrotron Radiation*); however, if you intend to submit to *Acta Crystallographica Section C* or *E* or *IUCrData*, you should make sure that full publication checks are run on the final version of your CIF prior to submission.

Publication of your CIF in other journals

Please refer to the *Notes for Authors* of the relevant journal for any special instructions relating to CIF submission.

PLATON version of 06/01/2024; check.def file version of 05/01/2024

Datablock JD_04_08_24_0m - ellipsoid plot

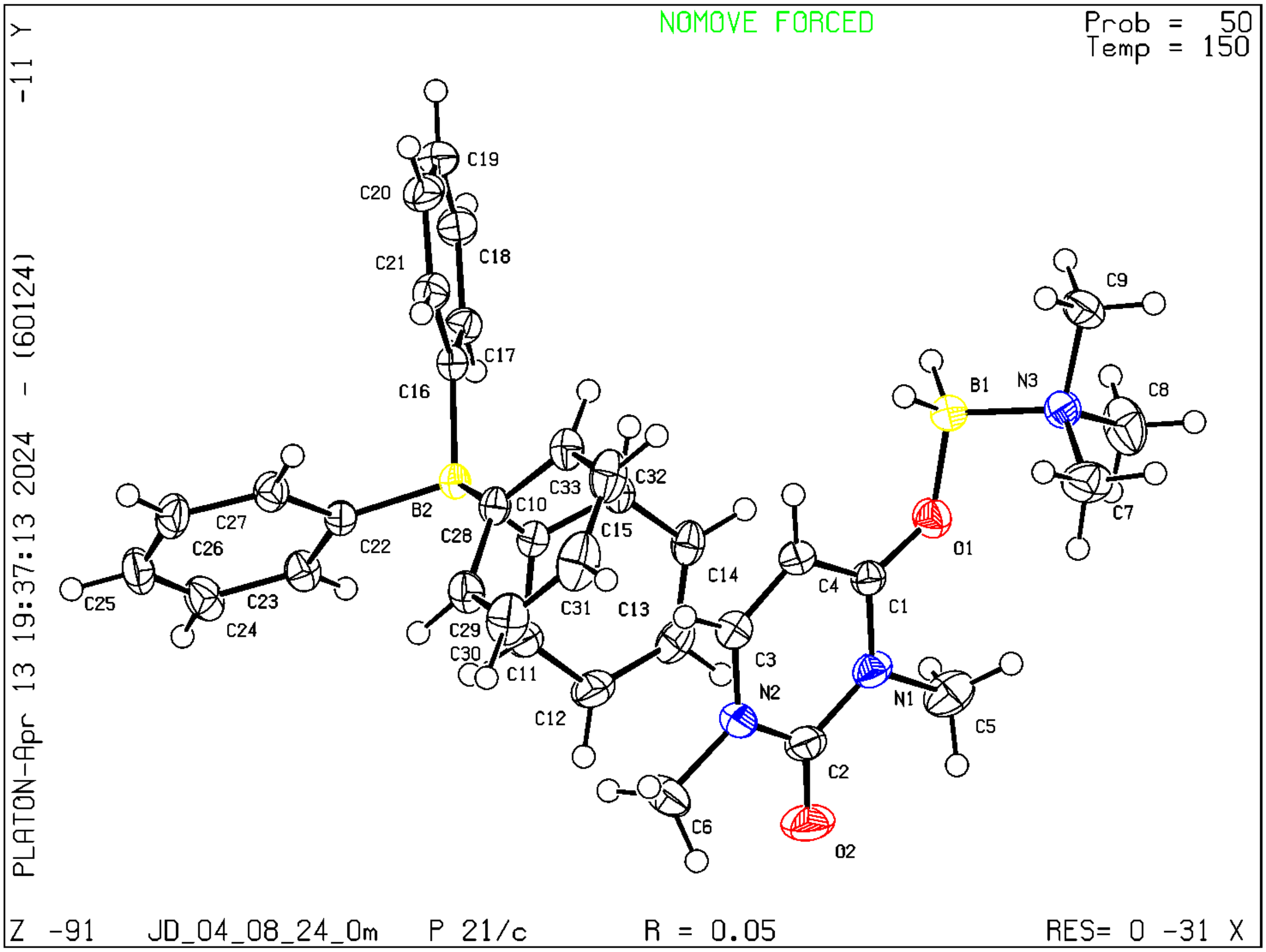

Download CIF editor (publCIF) from the IUCr
Download CIF editor (enCIFer) from the CCDC
Test a new CIF entry

①

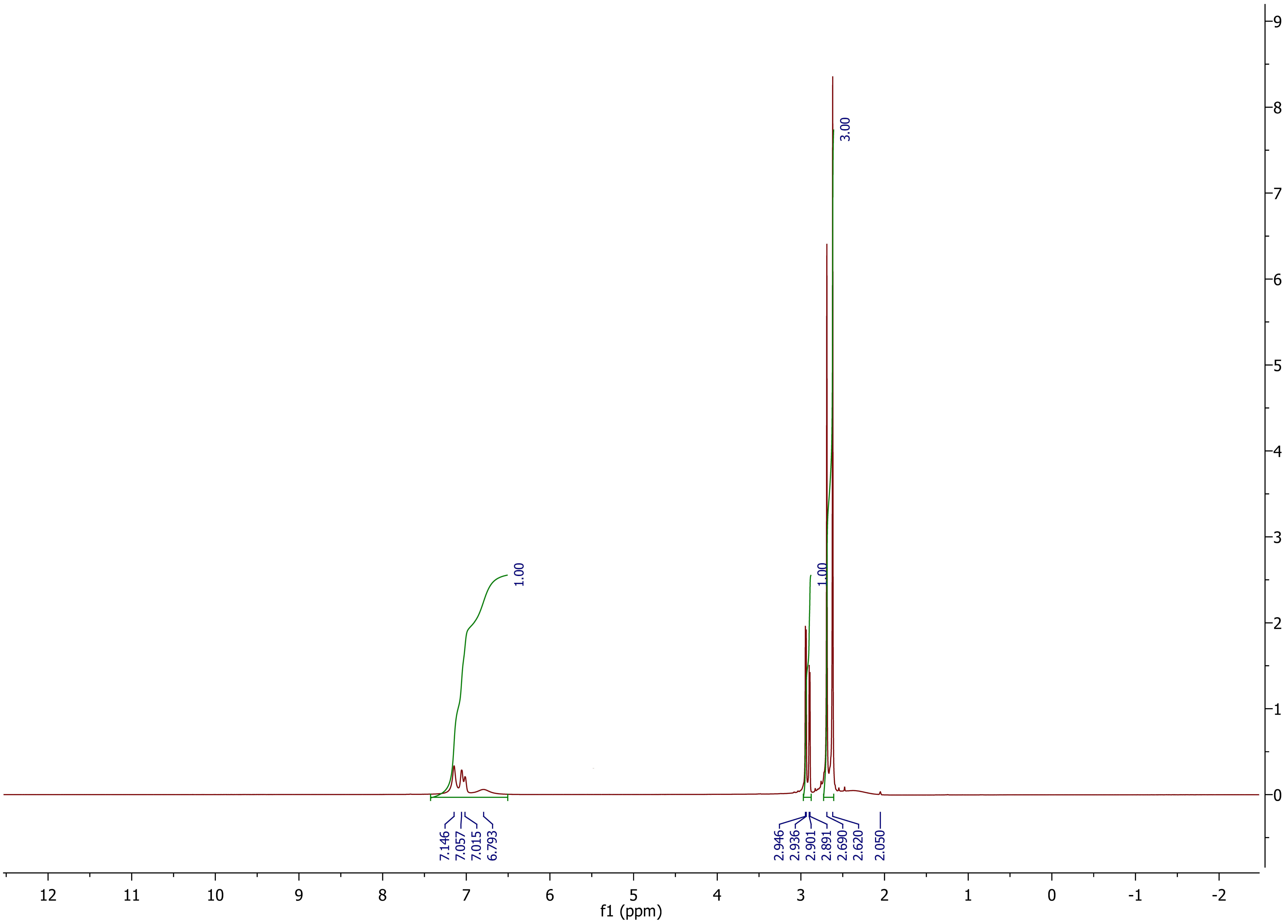

3.00
1.00
1.00
7.146
7.057
7.015
6.793
2.946
2.936
2.901
2.891
2.690
2.620
2.050
f1 (ppm)

①

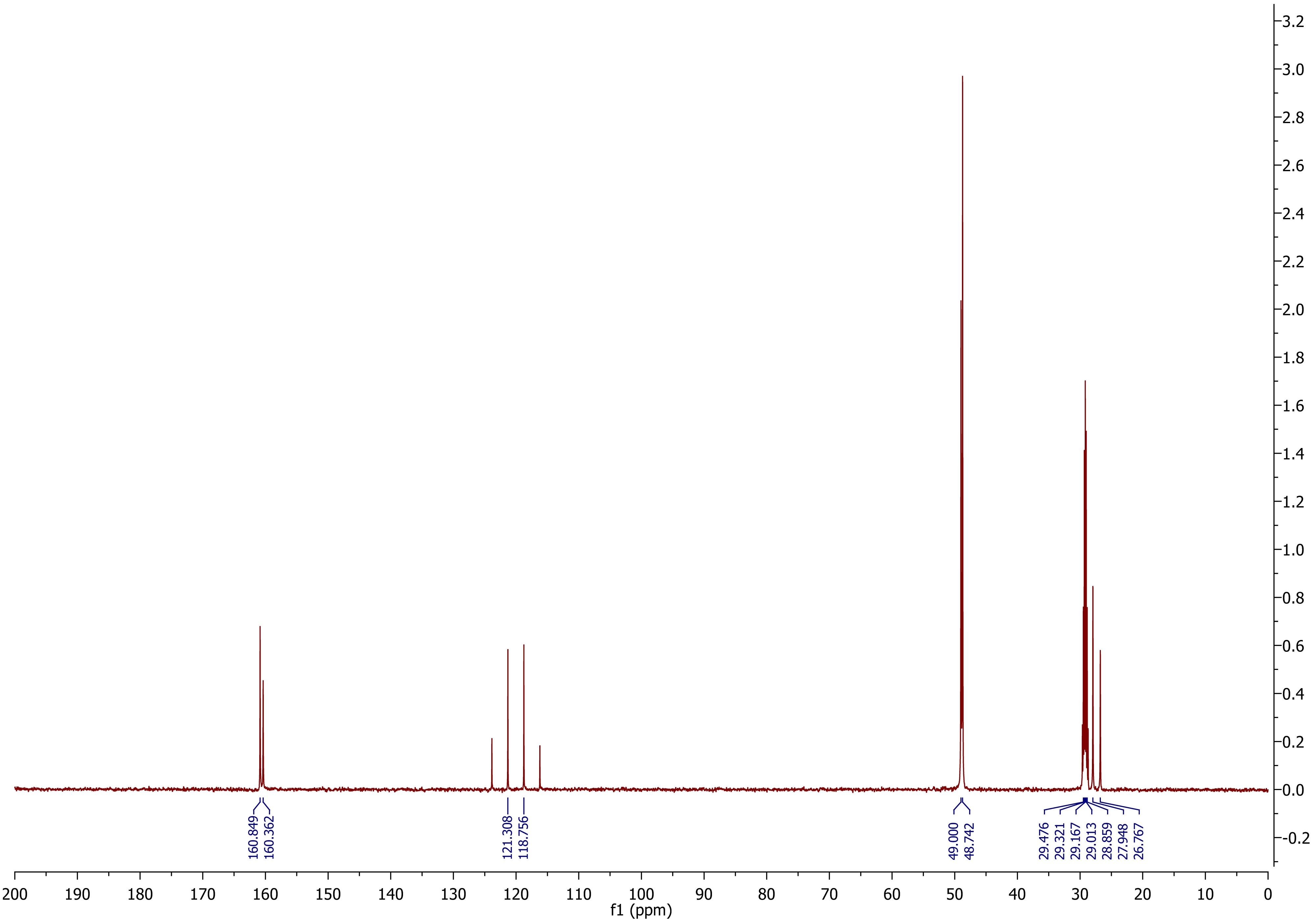

160.849
160.362
121.308
118.756
49.000
48.742
29.476
29.321
29.167
29.013
28.859
27.948
26.767
3.2
3.0
2.8
2.6
2.4
2.2
2.0
1.8
1.6
1.4
1.2
1.0
0.8
0.6
0.4
0.2
0.0
-0.2
200
190
180
170
160
150
140
130
120
110
100
90
80
70
60
50
40
30
20
10
0
f1 (ppm)

①

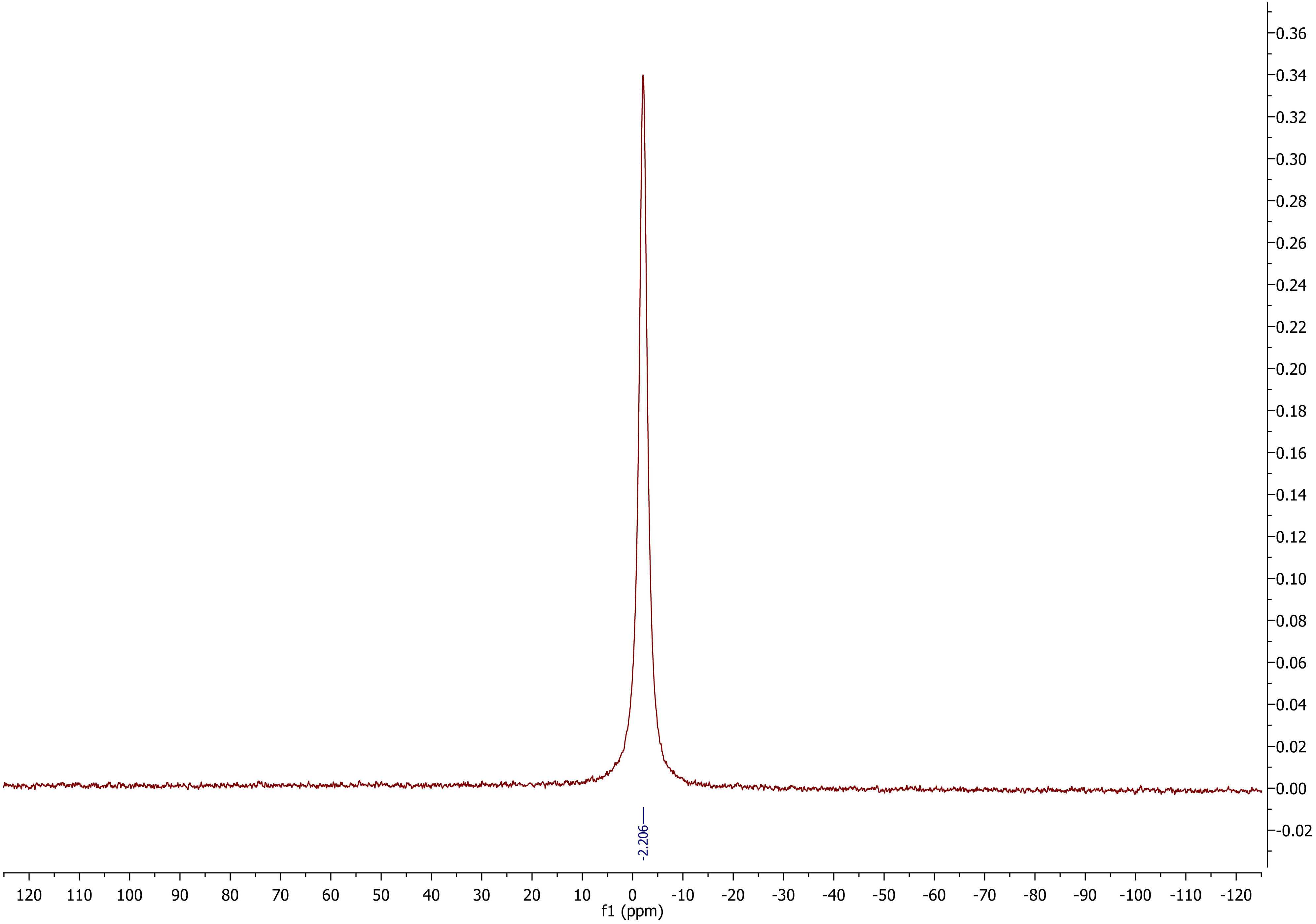

-2.206
120 110 100 90 80 70 60 50 40 30 20 10 0 -10 -20 -30 -40 -50 -60 -70 -80 -90 -100 -110 -120
f1 (ppm)
0.36 0.34 0.32 0.30 0.28 0.26 0.24 0.22 0.20 0.18 0.16 0.14 0.12 0.10 0.08 0.06 0.04 0.02 0.00 -0.02

①

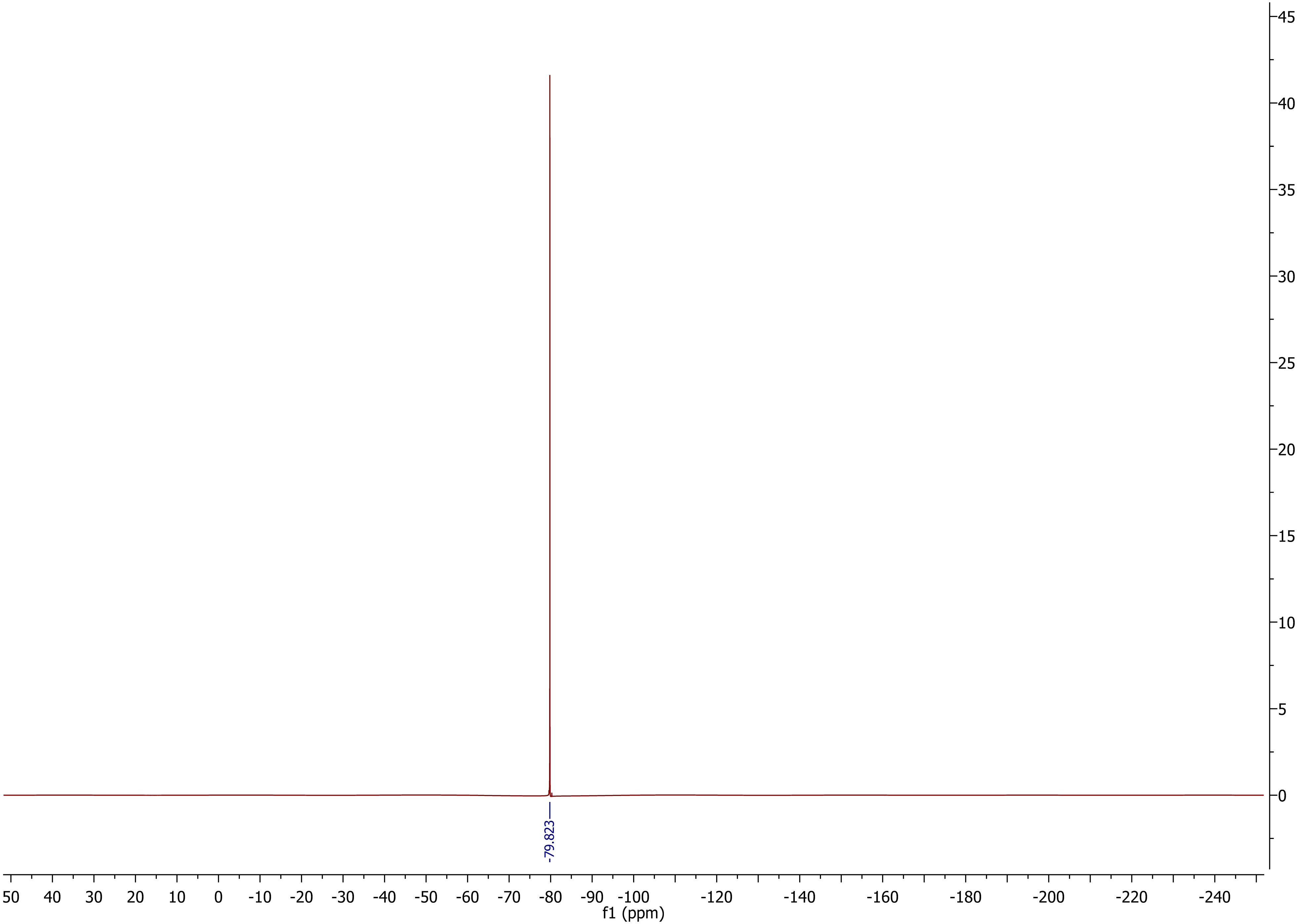
-79.823
50
40
30
20
10
0
-10
-20
-30
-40
-50
-60
-70
-80
-90
-100
-120
-140
-160
-180
-200
-220
-240
f1 (ppm)
45
40
35
30
25
20
15
10
5
0

②

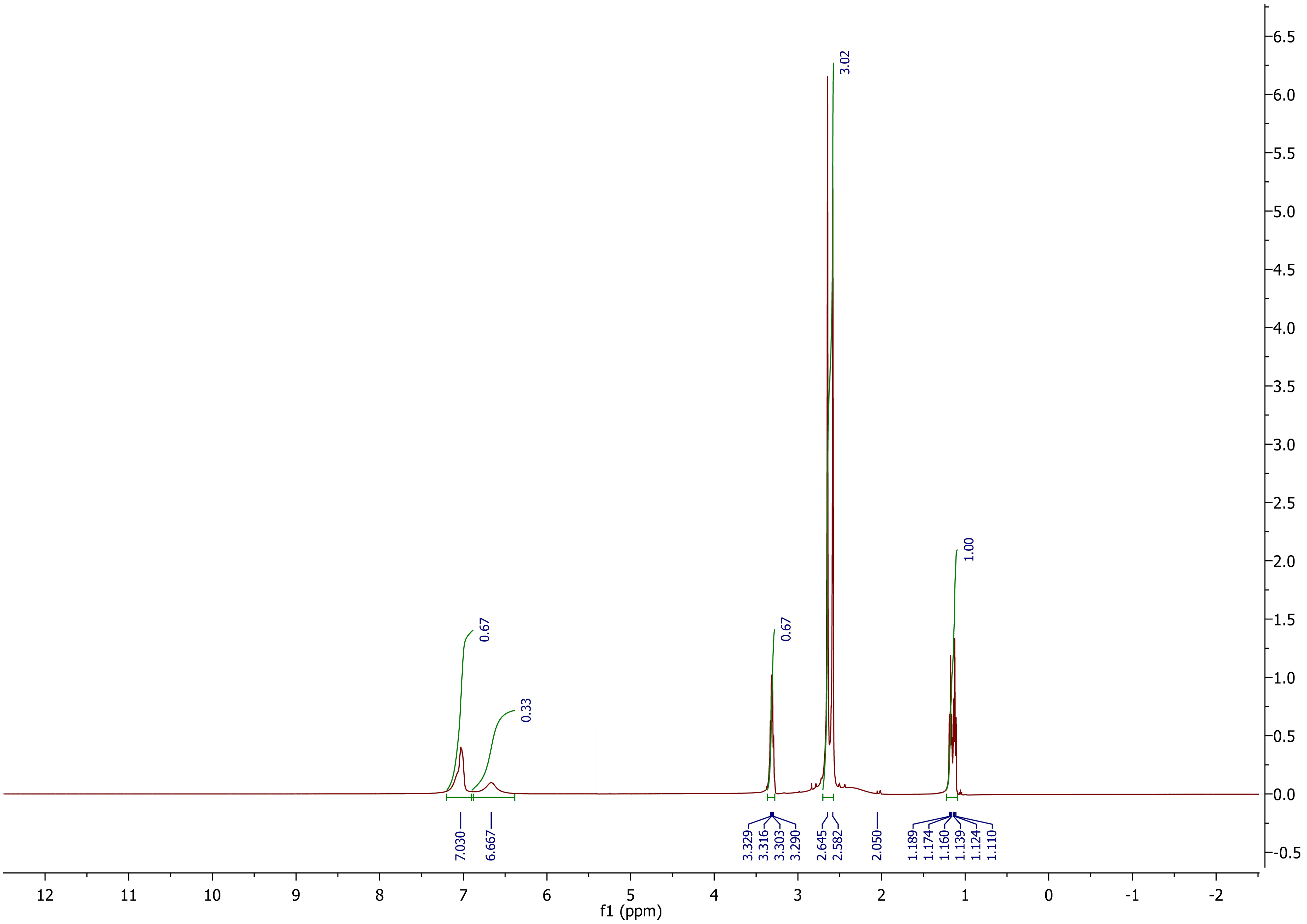

0.67
0.33
0.67
3.02
1.00
7.030
6.667
3.329
3.316
3.303
3.290
2.645
2.582
2.050
1.189
1.174
1.160
1.139
1.124
1.110
f1 (ppm)

②

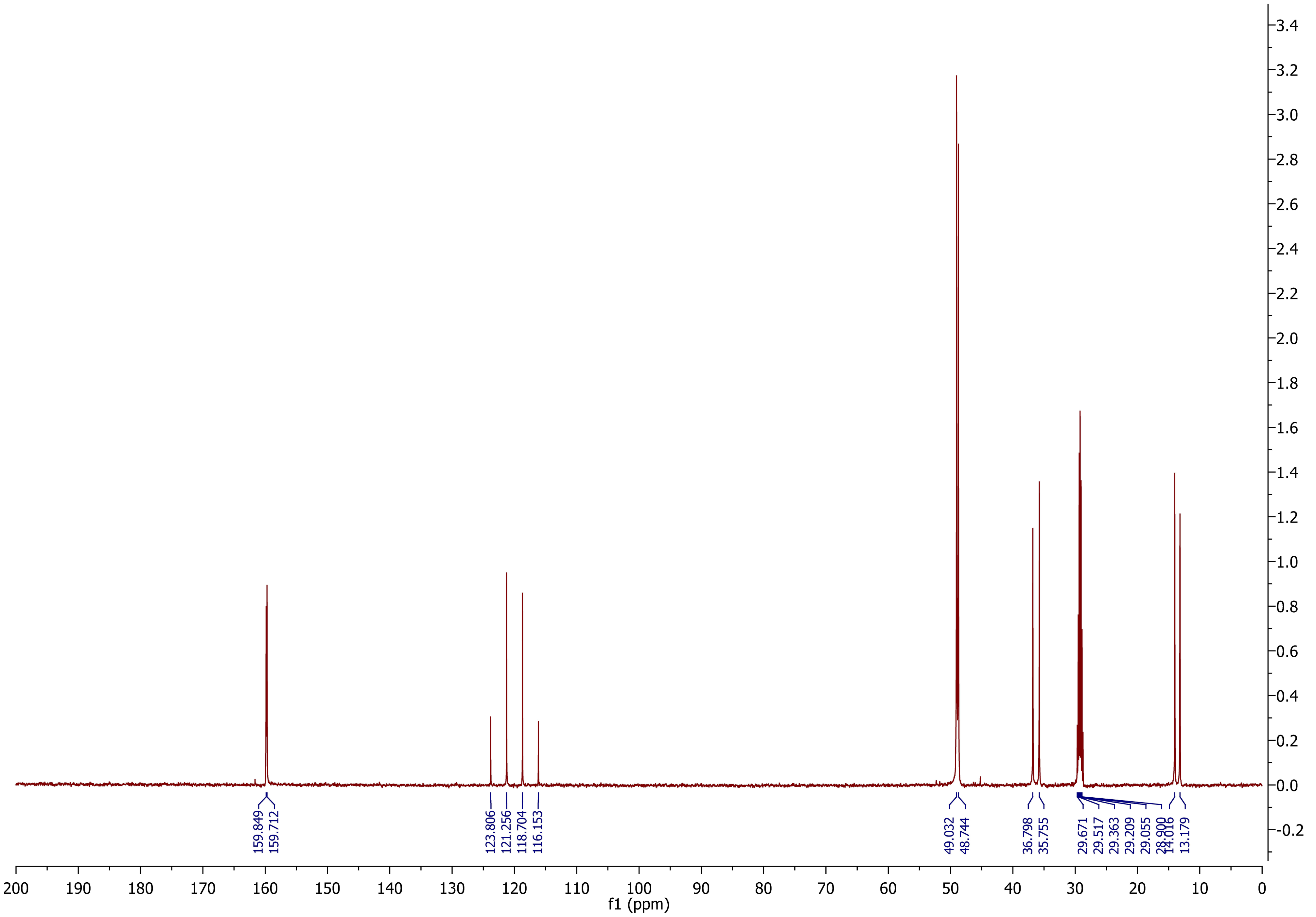

159.849
159.712
123.806
121.256
118.704
116.153
49.032
48.744
36.798
35.755
29.671
29.517
29.363
29.209
29.055
28.900
14.016
13.179
f1 (ppm)

②

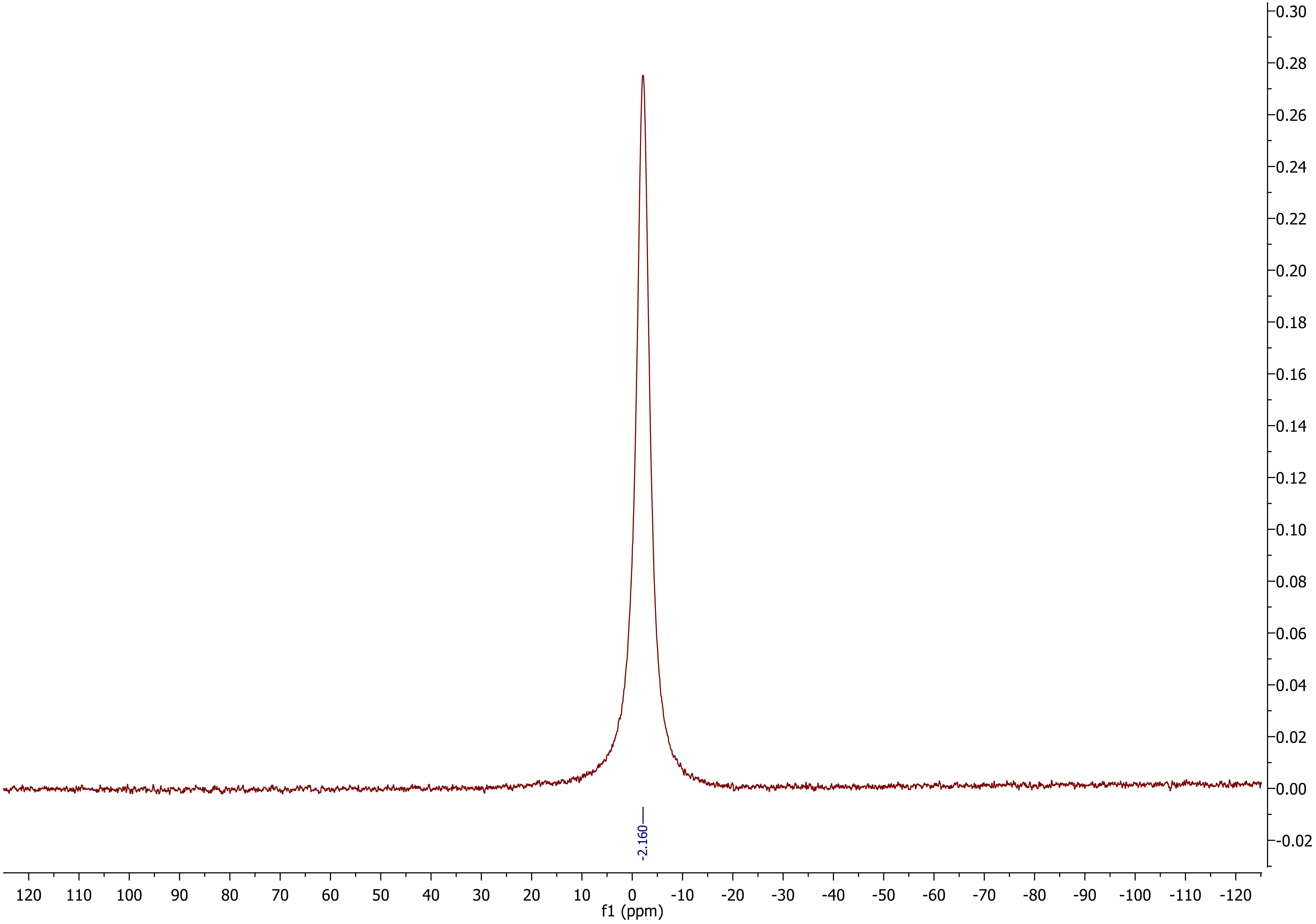

0.30
0.28
0.26
0.24
0.22
0.20
0.18
0.16
0.14
0.12
0.10
0.08
0.06
0.04
0.02
0.00
-0.02
-2.160
120 110 100 90 80 70 60 50 40 30 20 10 0 -10 -20 -30 -40 -50 -60 -70 -80 -90 -100 -110 -120
f1 (ppm)

②

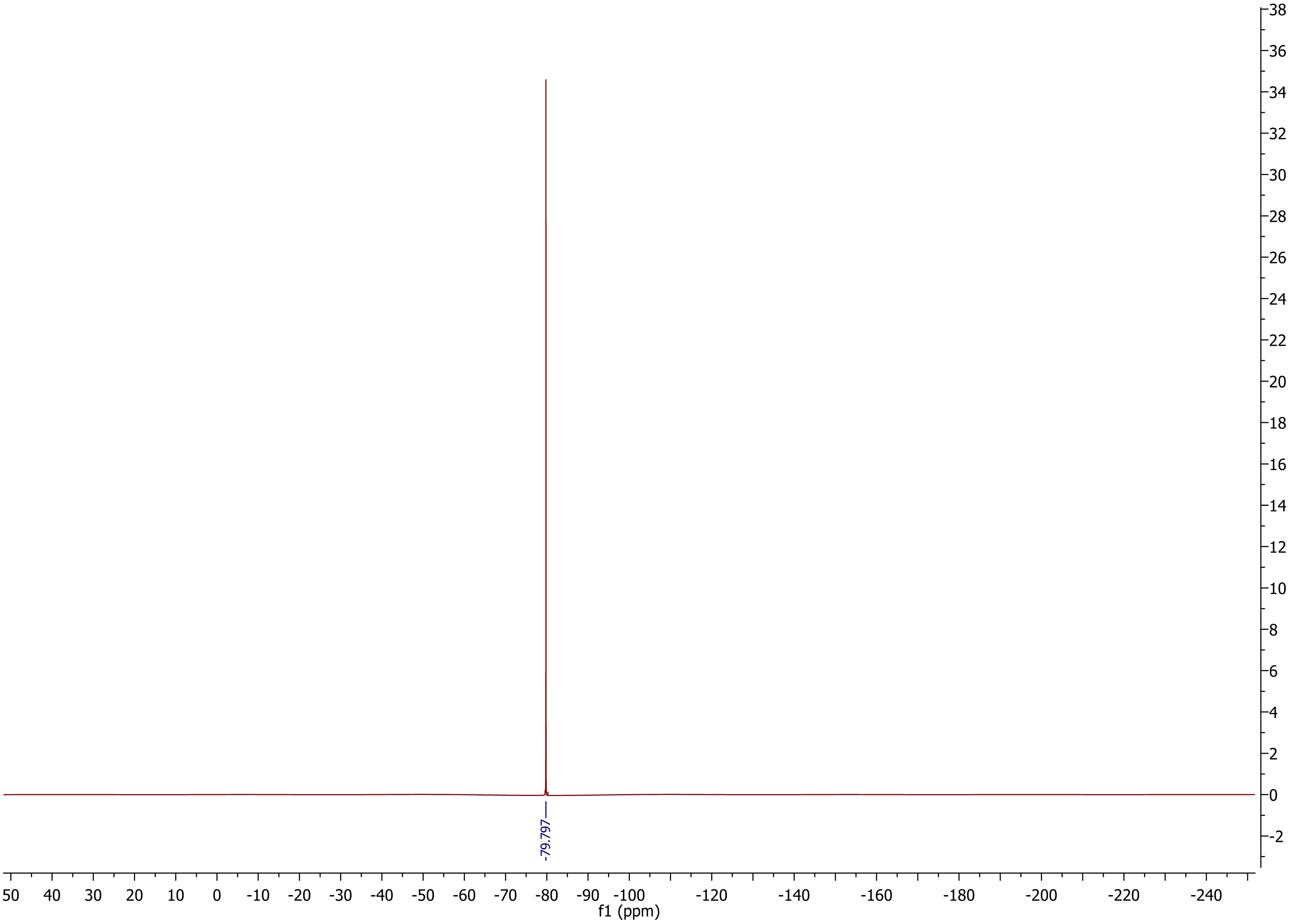

-79.797
50 40 30 20 10 0 -10 -20 -30 -40 -50 -60 -70 -80 -90 -100 -120 -140 -160 -180 -200 -220 -240
f1 (ppm)
38 36 34 32 30 28 26 24 22 20 18 16 14 12 10 8 6 4 2 0 -2

③

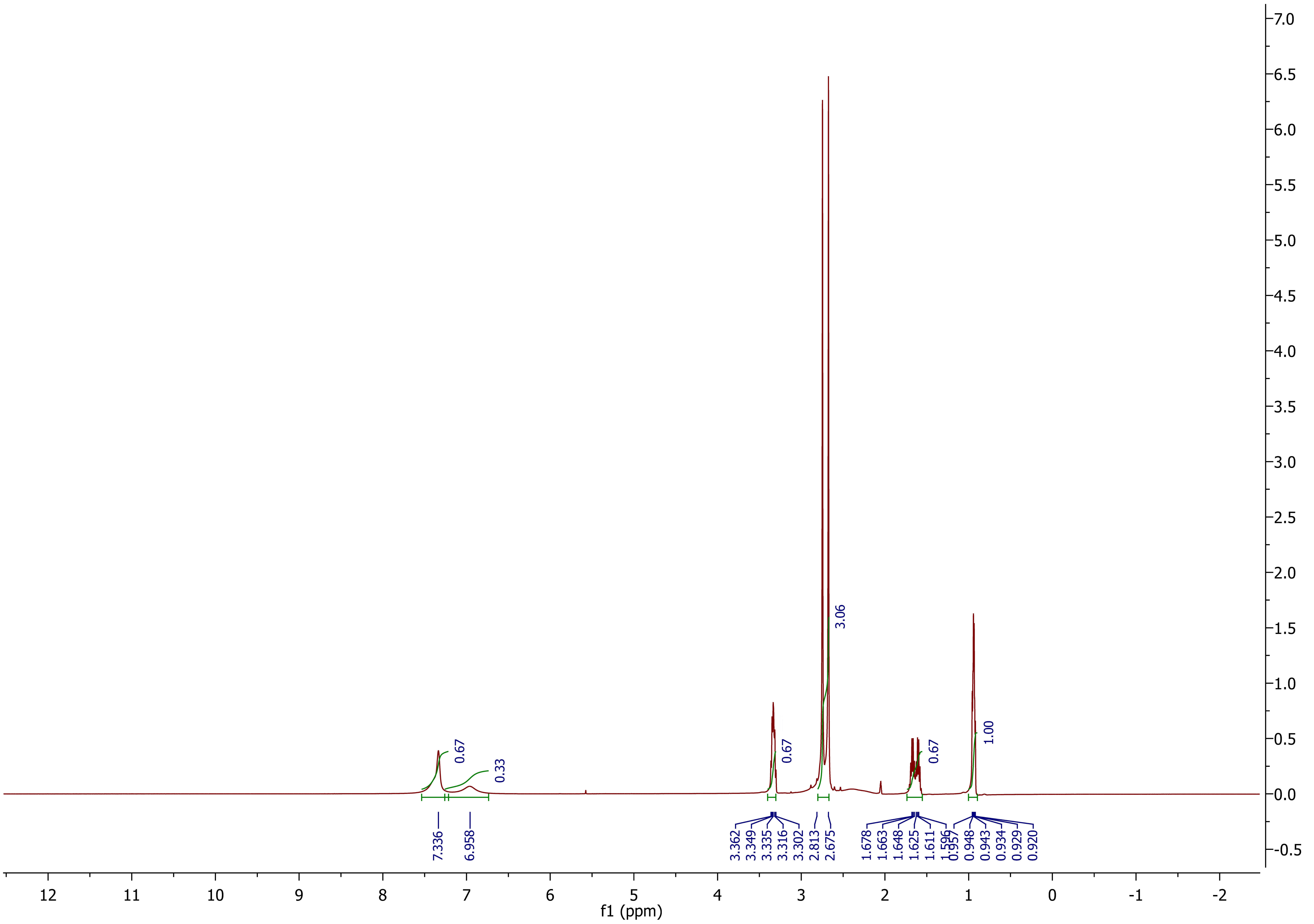

7.336
6.958
3.362
3.349
3.335
3.316
3.302
2.813
2.675
1.678
1.663
1.648
1.625
1.611
1.596
0.957
0.948
0.943
0.934
0.929
0.920
0.67
0.33
0.67
3.06
0.67
1.00
f1 (ppm)

③

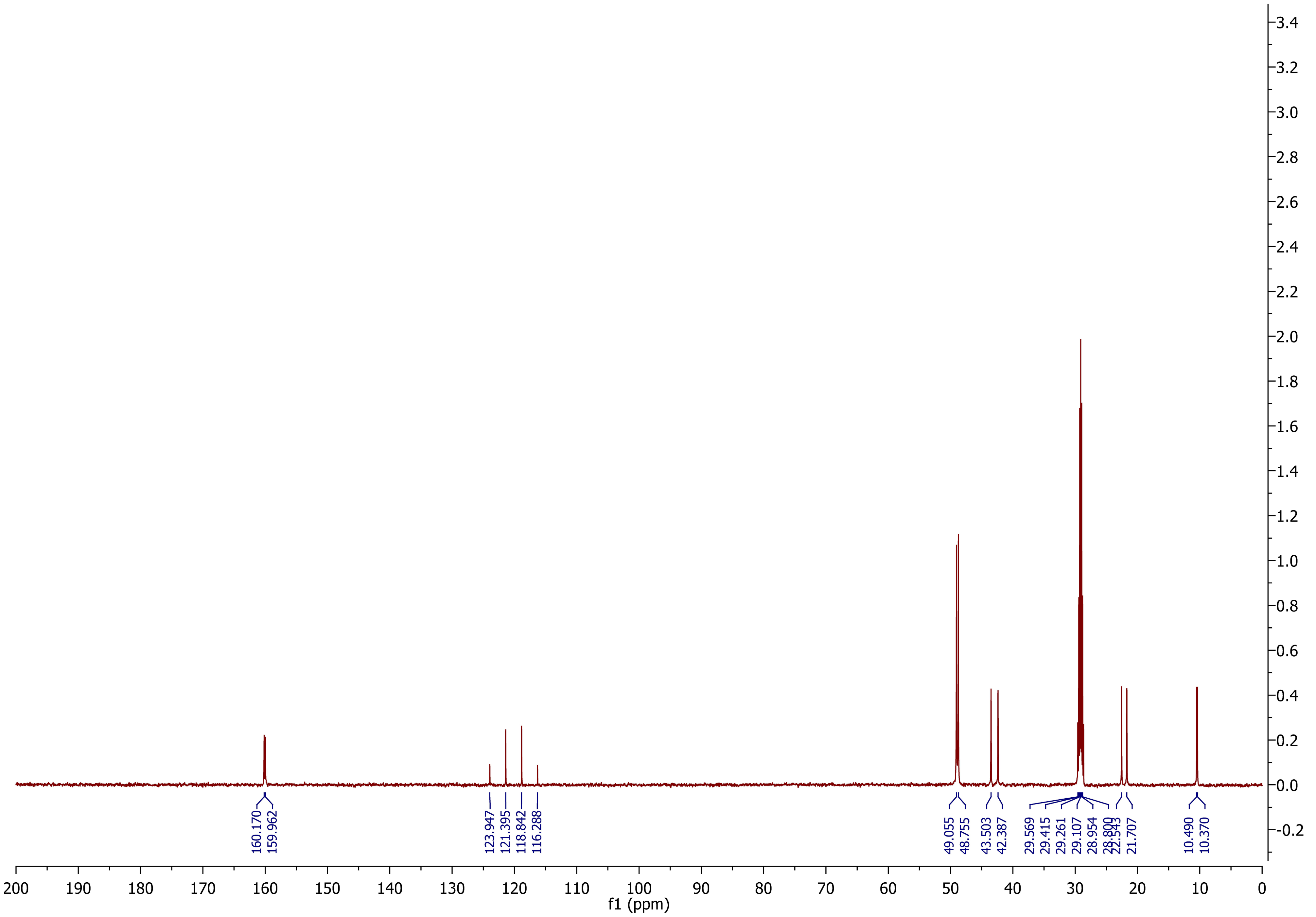

160.170
159.962
123.947
121.395
118.842
116.288
49.055
48.755
43.503
42.387
29.569
29.415
29.261
29.107
28.954
28.800
22.543
21.707
10.490
10.370
f1 (ppm)

③

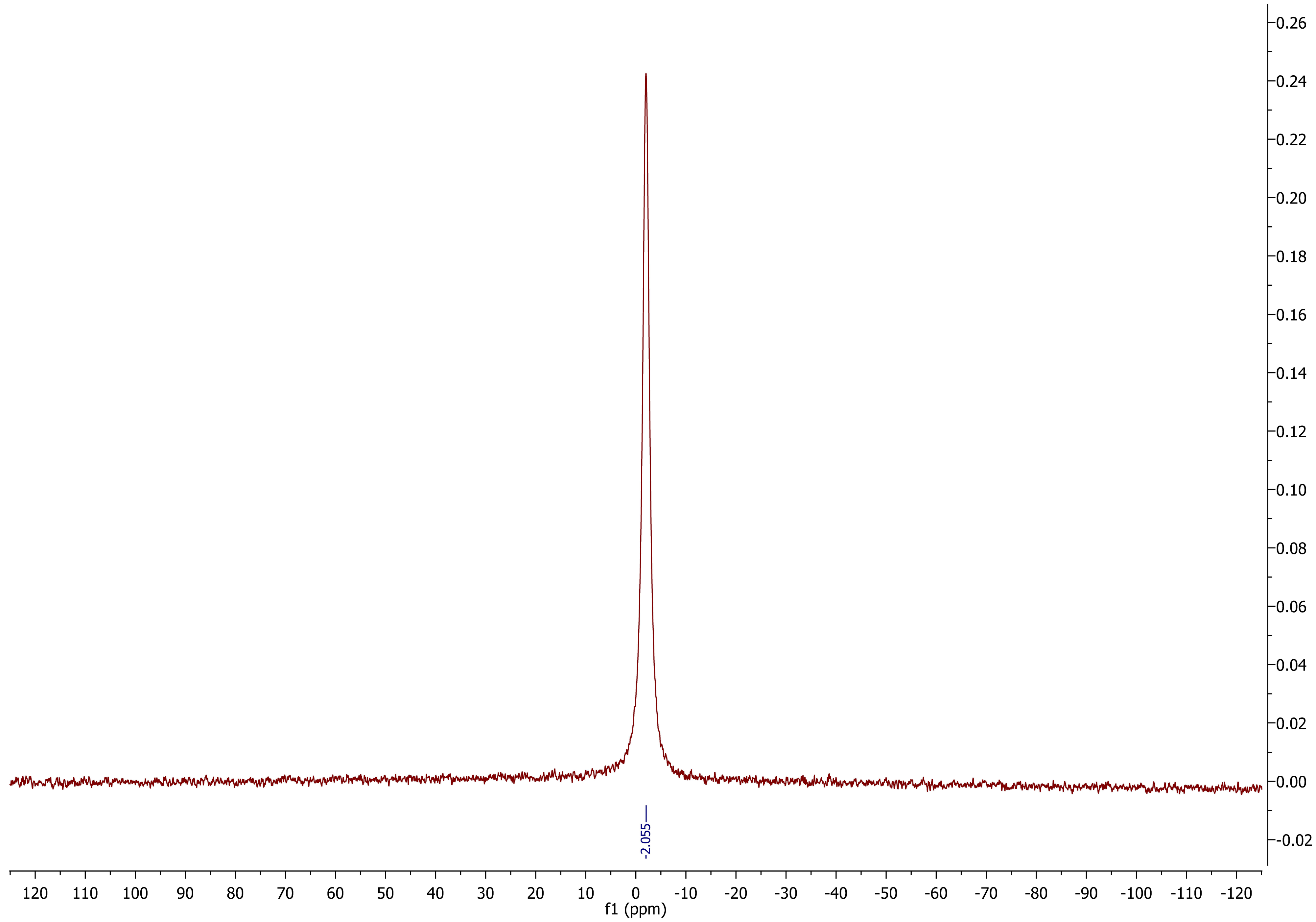

-2.055
0.26
0.24
0.22
0.20
0.18
0.16
0.14
0.12
0.10
0.08
0.06
0.04
0.02
0.00
-0.02
120 110 100 90 80 70 60 50 40 30 20 10 0 -10 -20 -30 -40 -50 -60 -70 -80 -90 -100 -110 -120
f1 (ppm)

③

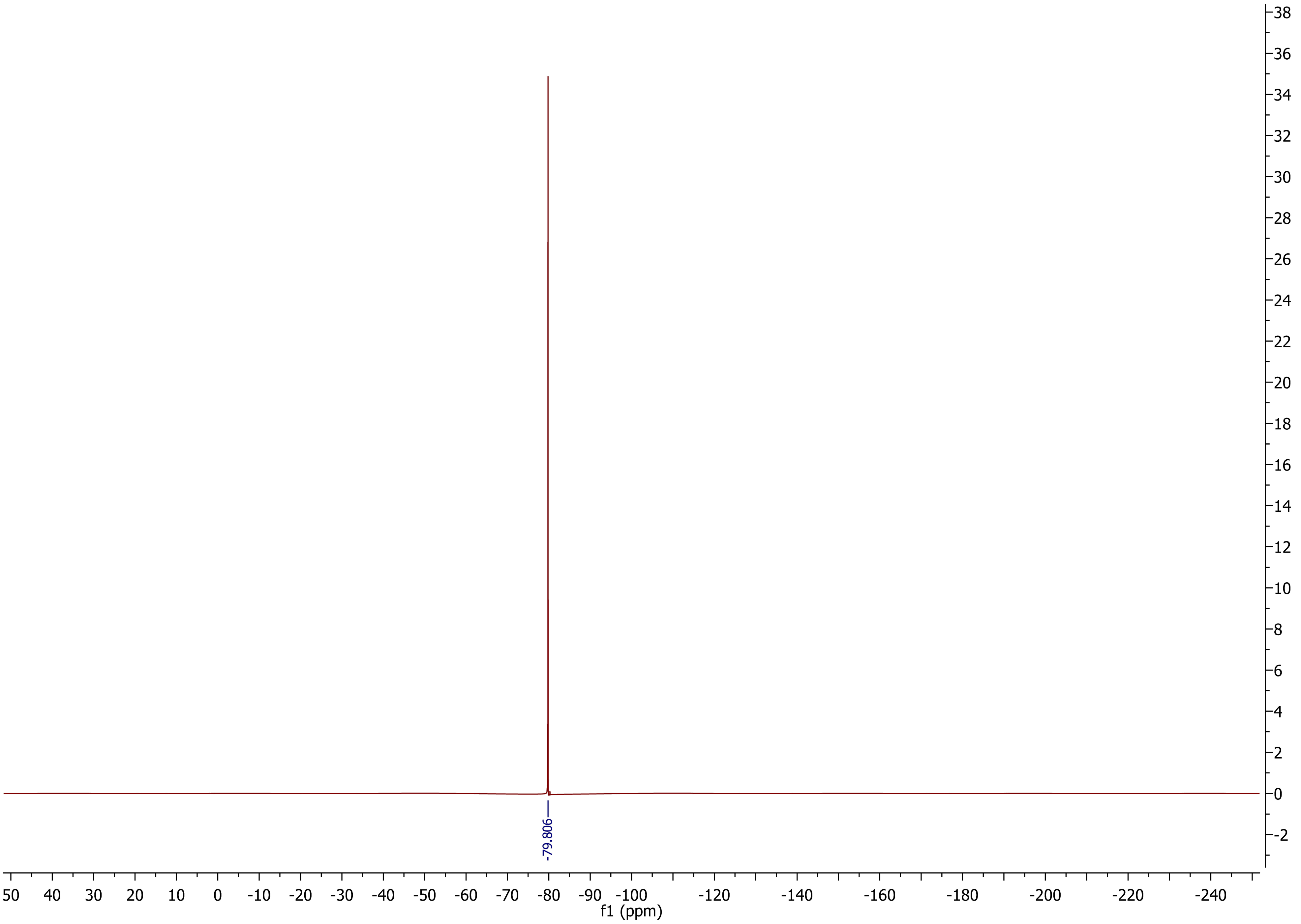

-79.806
50
40
30
20
10
0
-10
-20
-30
-40
-50
-60
-70
-80
-90
-100
-120
-140
-160
-180
-200
-220
-240
f1 (ppm)
38
36
34
32
30
28
26
24
22
20
18
16
14
12
10
8
6
4
2
0
-2

④

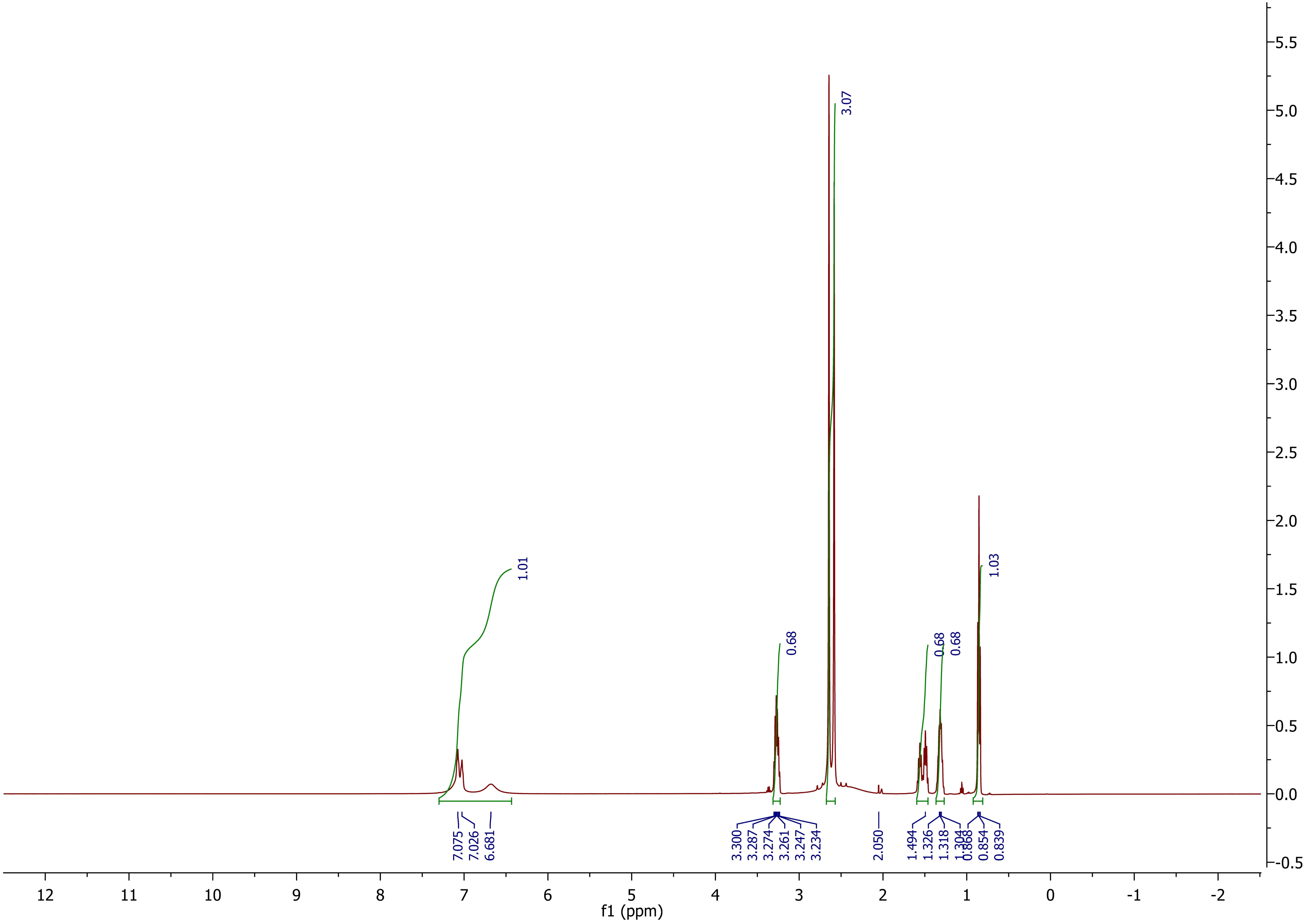

1.01
0.68
3.07
0.68
0.68
1.03
7.075
7.026
6.681
3.300
3.287
3.274
3.261
3.247
3.234
2.050
1.494
1.326
1.318
1.304
0.868
0.854
0.839
f1 (ppm)

④

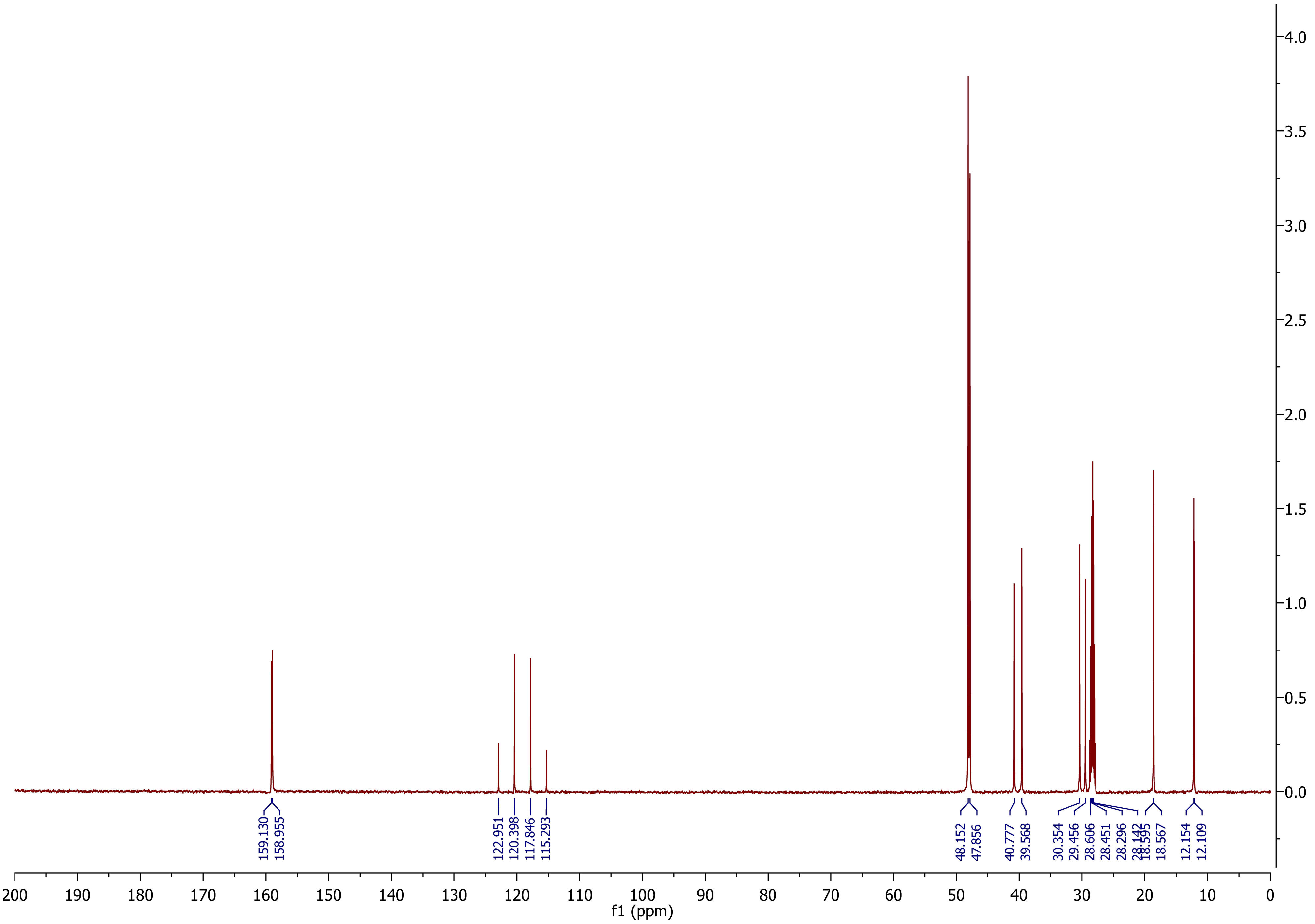
159.130
158.955
122.951
120.398
117.846
115.293
48.152
47.856
40.777
39.568
30.354
29.456
28.606
28.451
28.296
28.142
18.595
18.567
12.154
12.109
4.0
3.5
3.0
2.5
2.0
1.5
1.0
0.5
0.0
200
190
180
170
160
150
140
130
120
110
100
90
80
70
60
50
40
30
20
10
0
f1 (ppm)

④

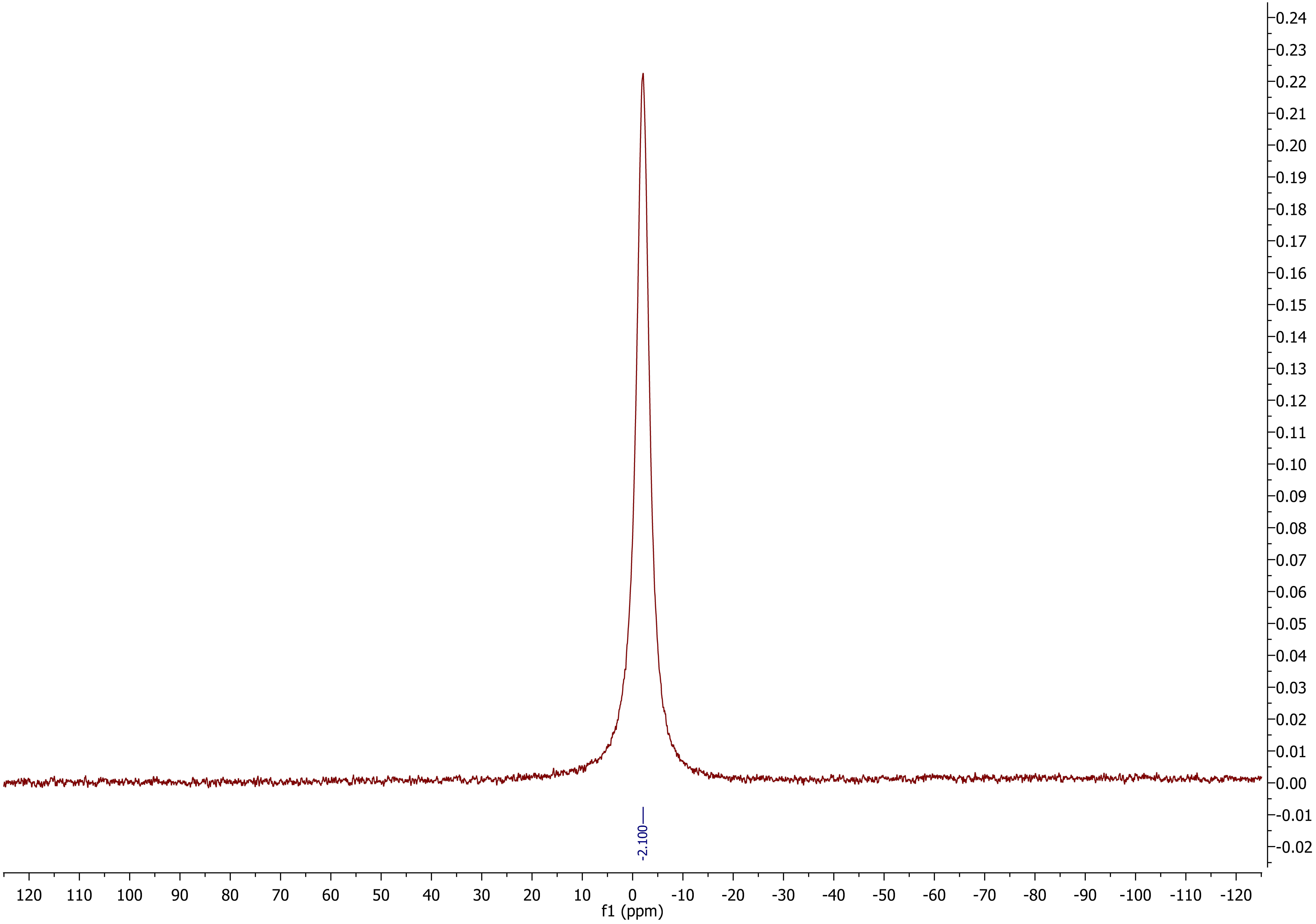
-2.100
f1 (ppm)
120
110
100
90
80
70
60
50
40
30
20
10
0
-10
-20
-30
-40
-50
-60
-70
-80
-90
-100
-110
-120
0.24
0.23
0.22
0.21
0.20
0.19
0.18
0.17
0.16
0.15
0.14
0.13
0.12
0.11
0.10
0.09
0.08
0.07
0.06
0.05
0.04
0.03
0.02
0.01
0.00
-0.01
-0.02

④

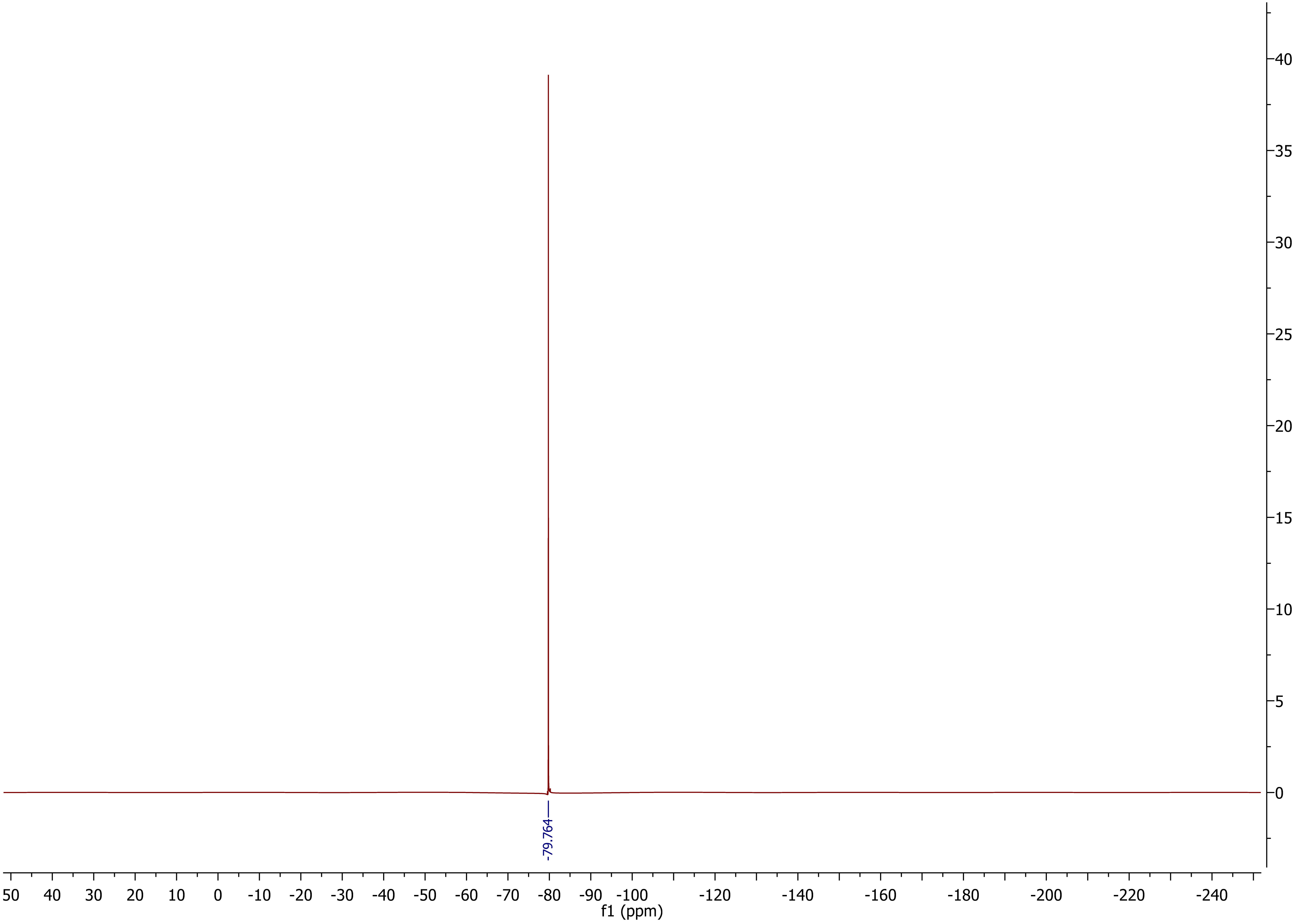
-79.764
50 40 30 20 10 0 -10 -20 -30 -40 -50 -60 -70 -80 -90 -100 -120 -140 -160 -180 -200 -220 -240
f1 (ppm)
40 35 30 25 20 15 10 5 0

⑤

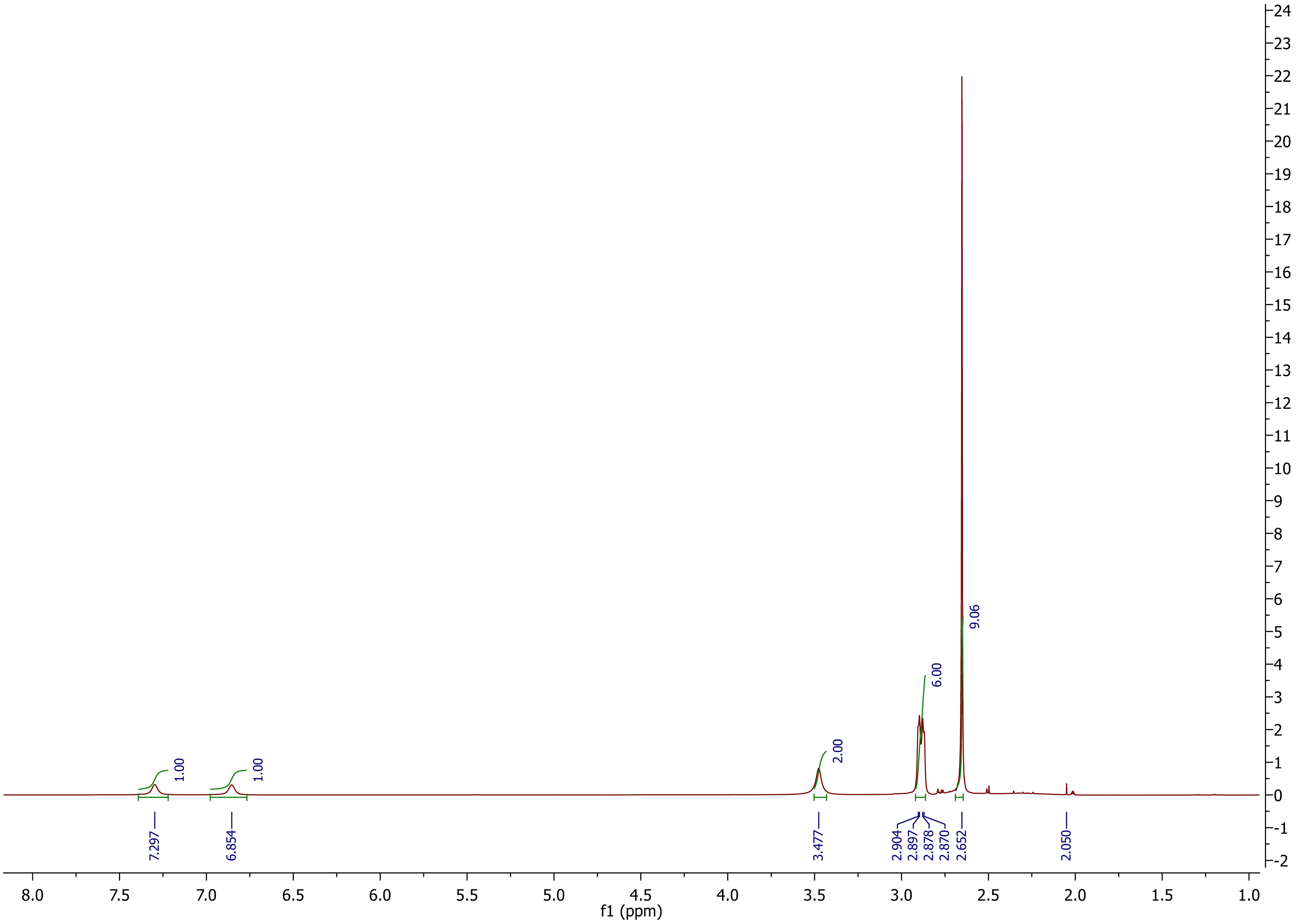

1.00
1.00
2.00
6.00
9.06
7.297
6.854
3.477
2.904
2.897
2.878
2.870
2.652
2.050
f1 (ppm)

⑤

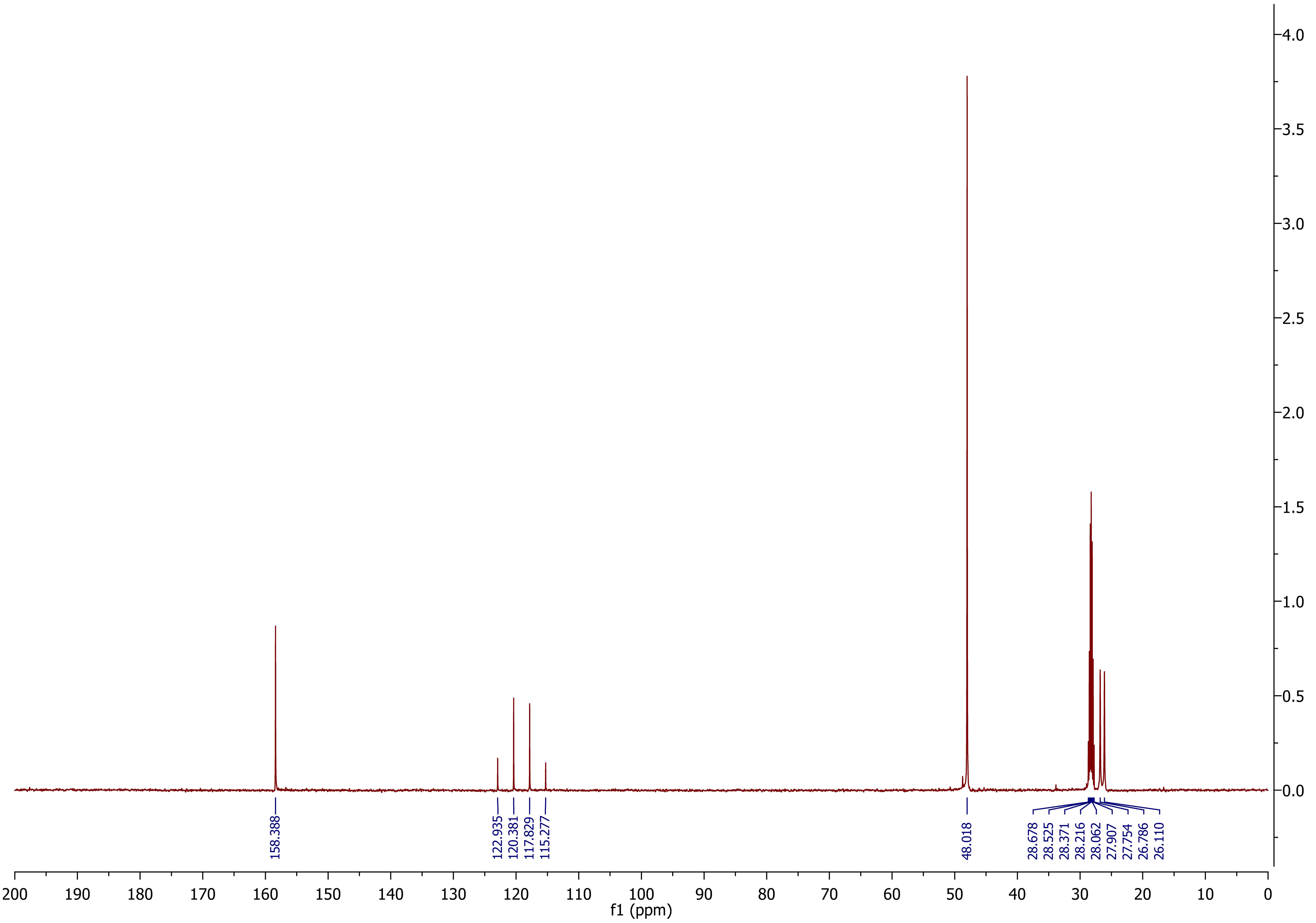
158.388
122.935
120.381
117.829
115.277
48.018
28.678
28.525
28.371
28.216
28.062
27.907
27.754
26.786
26.110
4.0
3.5
3.0
2.5
2.0
1.5
1.0
0.5
0.0
200 190 180 170 160 150 140 130 120 110 100 90 80 70 60 50 40 30 20 10 0
f1 (ppm)

⑤

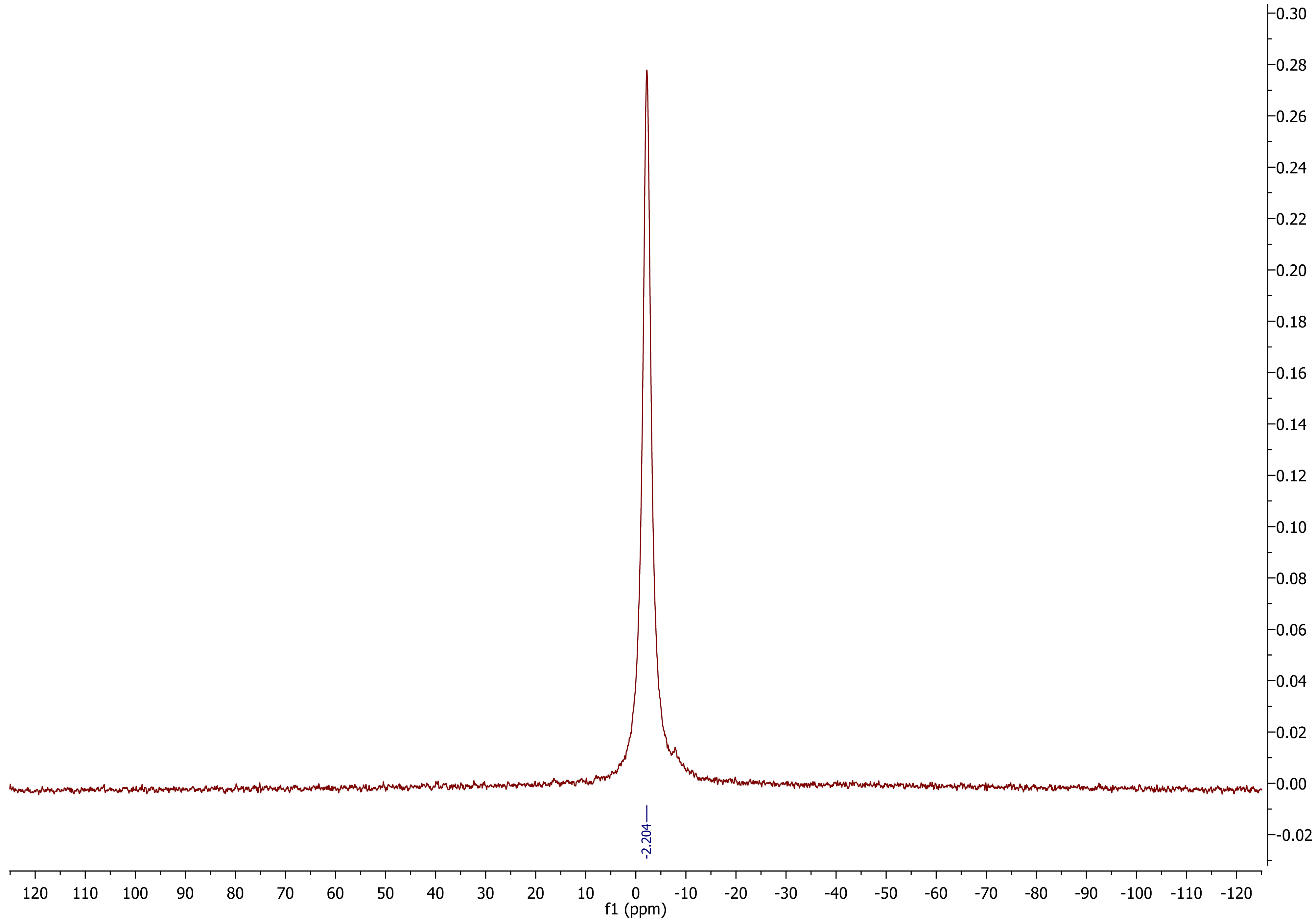

0.30
0.28
0.26
0.24
0.22
0.20
0.18
0.16
0.14
0.12
0.10
0.08
0.06
0.04
0.02
0.00
-0.02
-2.204
120 110 100 90 80 70 60 50 40 30 20 10 0 -10 -20 -30 -40 -50 -60 -70 -80 -90 -100 -110 -120
f1 (ppm)

⑤

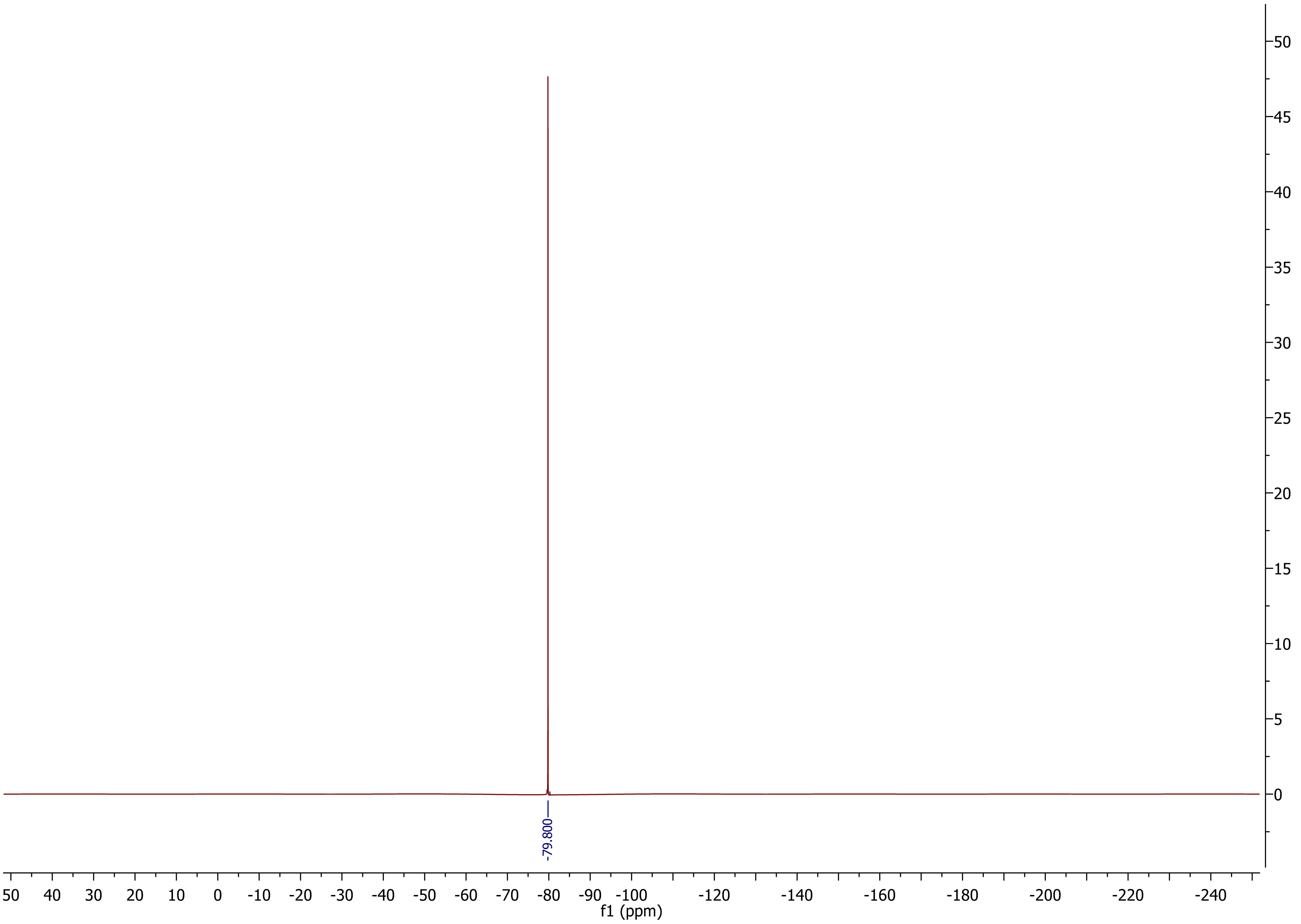

-79.800
50 40 30 20 10 0 -10 -20 -30 -40 -50 -60 -70 -80 -90 -100 -120 -140 -160 -180 -200 -220 -240
f1 (ppm)
50 45 40 35 30 25 20 15 10 5 0

⑥

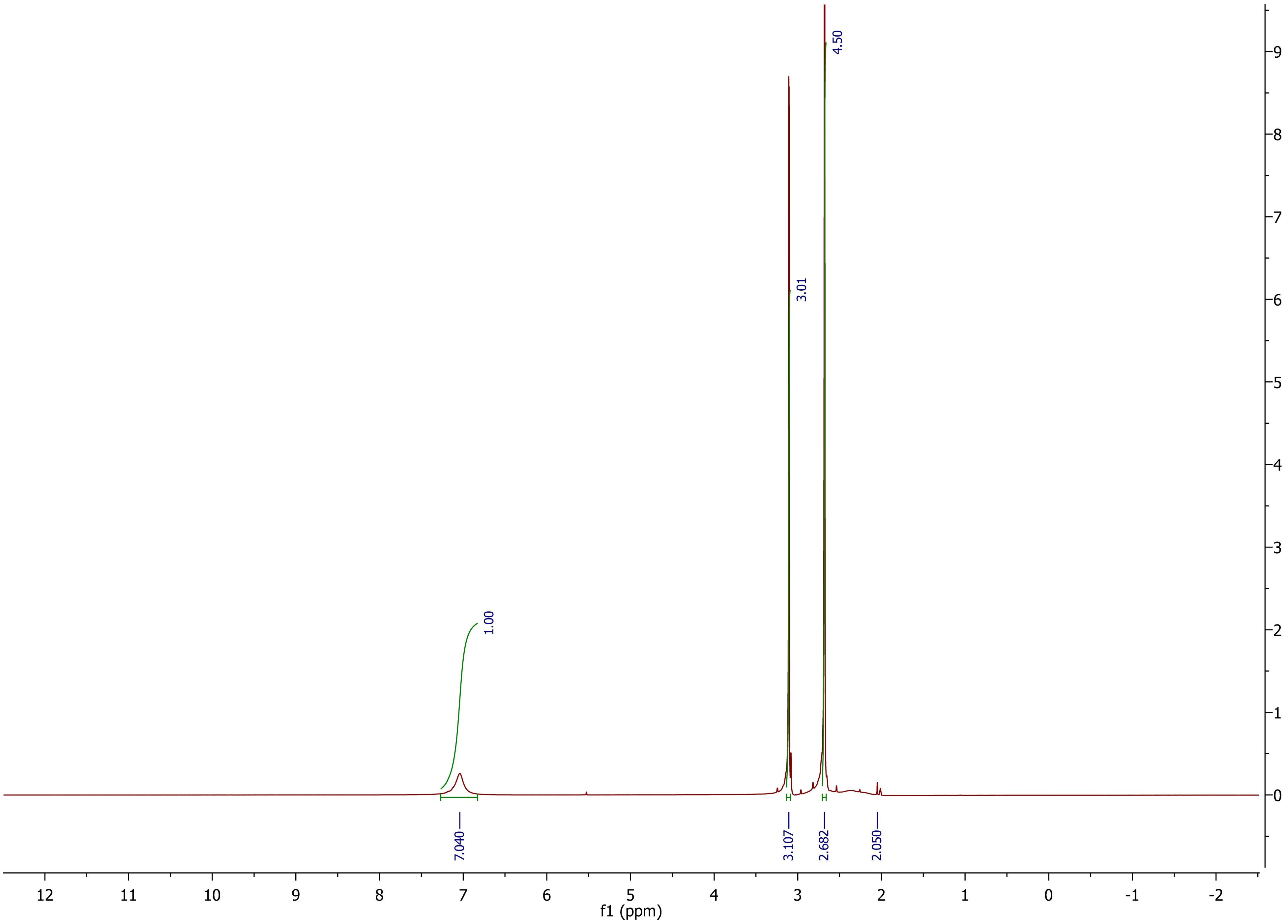

⑥

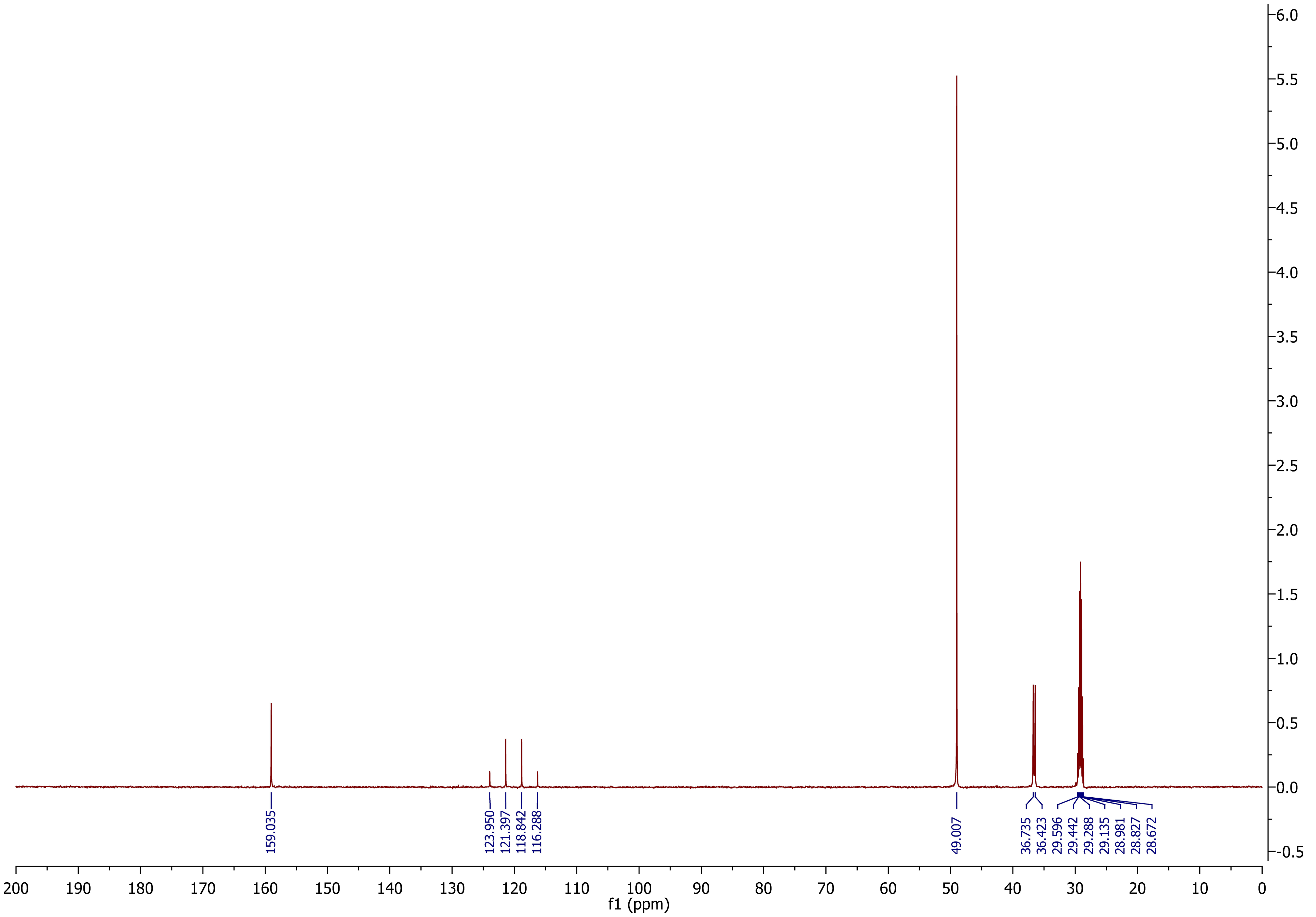

159.035
123.950
121.397
118.842
116.288
49.007
36.735
36.423
29.596
29.442
29.288
29.135
28.981
28.827
28.672
6.0
5.5
5.0
4.5
4.0
3.5
3.0
2.5
2.0
1.5
1.0
0.5
0.0
-0.5
200 190 180 170 160 150 140 130 120 110 100 90 80 70 60 50 40 30 20 10 0
f1 (ppm)

⑥

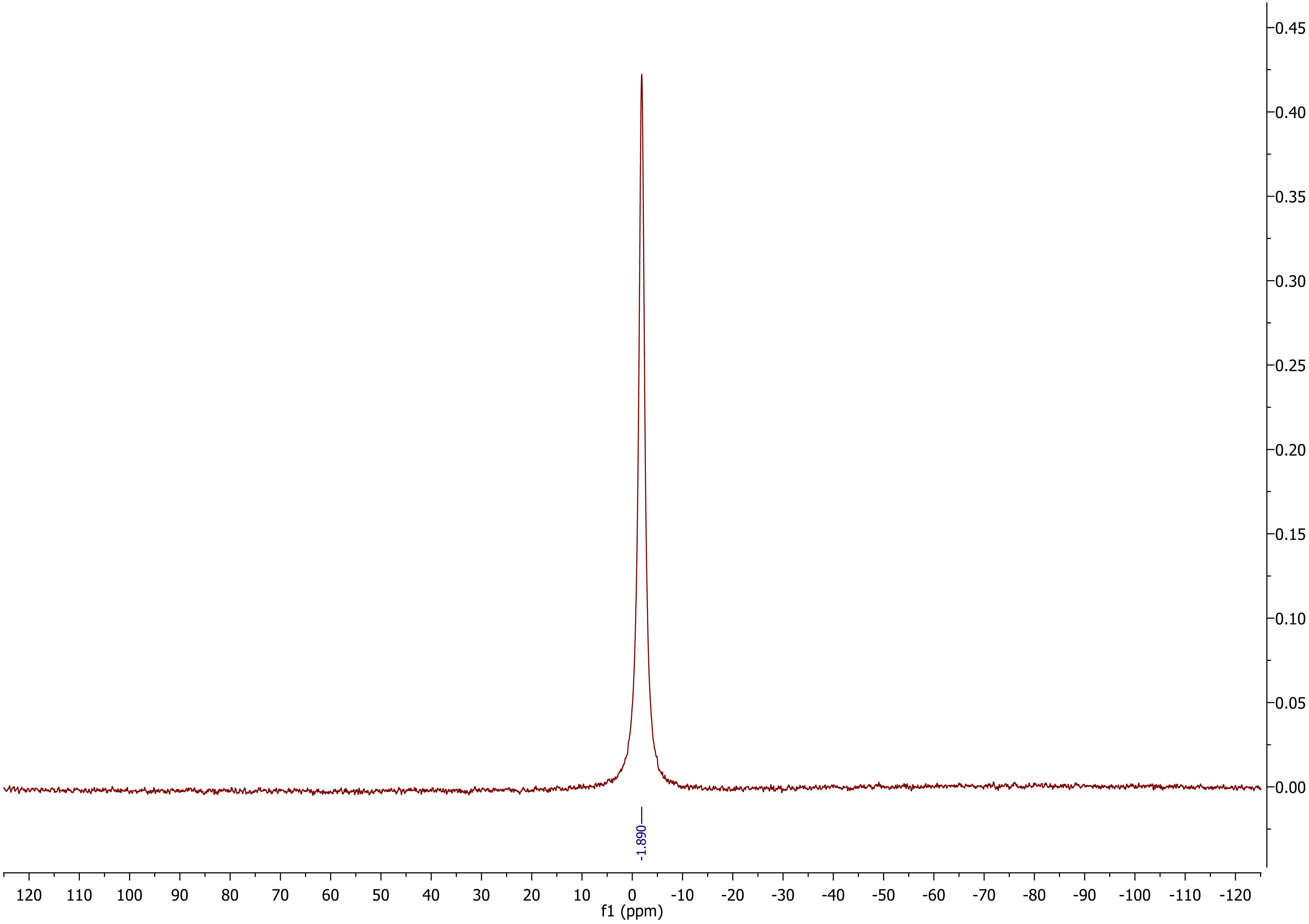

-1.890
0.45
0.40
0.35
0.30
0.25
0.20
0.15
0.10
0.05
0.00
120 110 100 90 80 70 60 50 40 30 20 10 0 -10 -20 -30 -40 -50 -60 -70 -80 -90 -100 -110 -120
f1 (ppm)

⑥

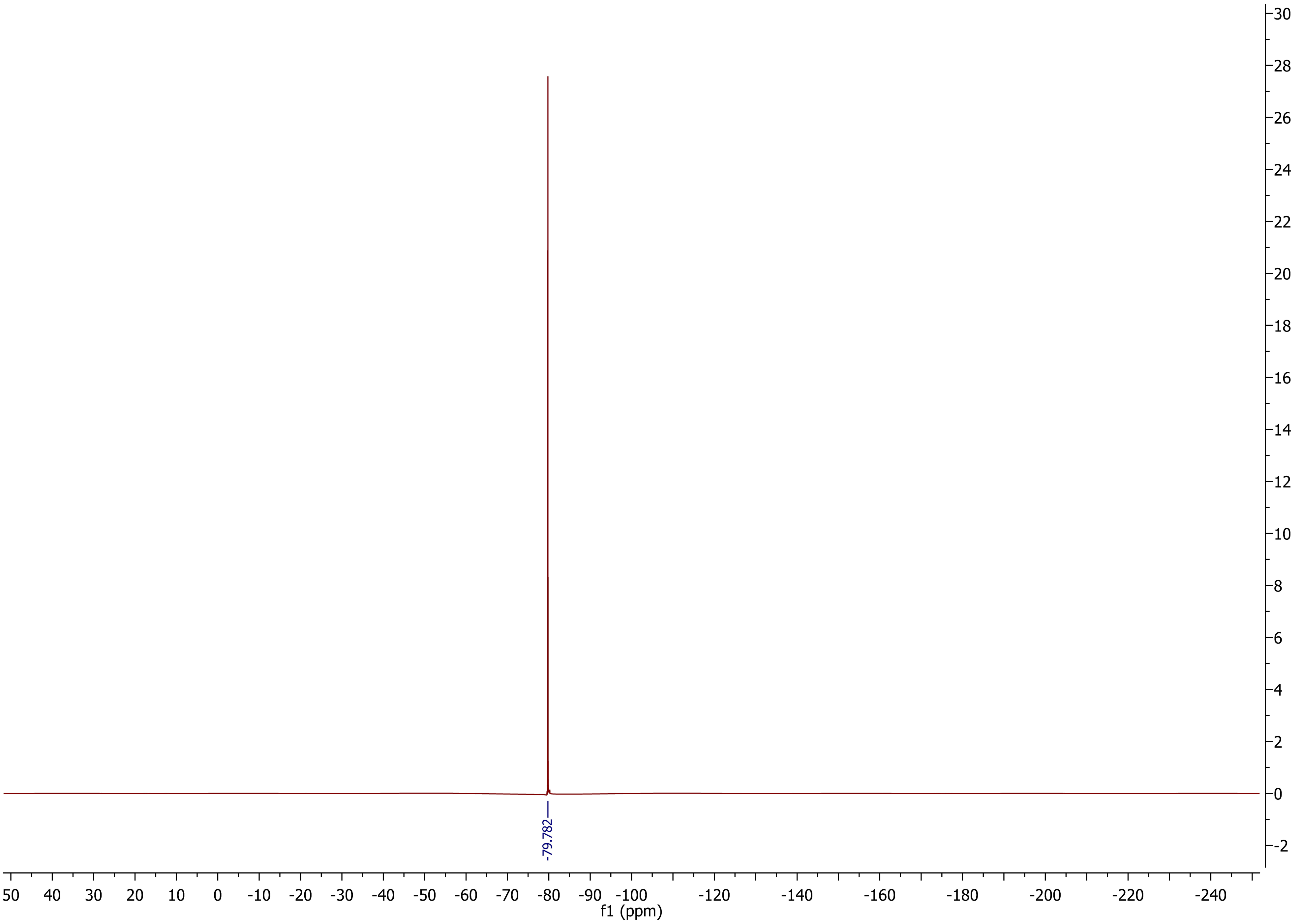

⑦

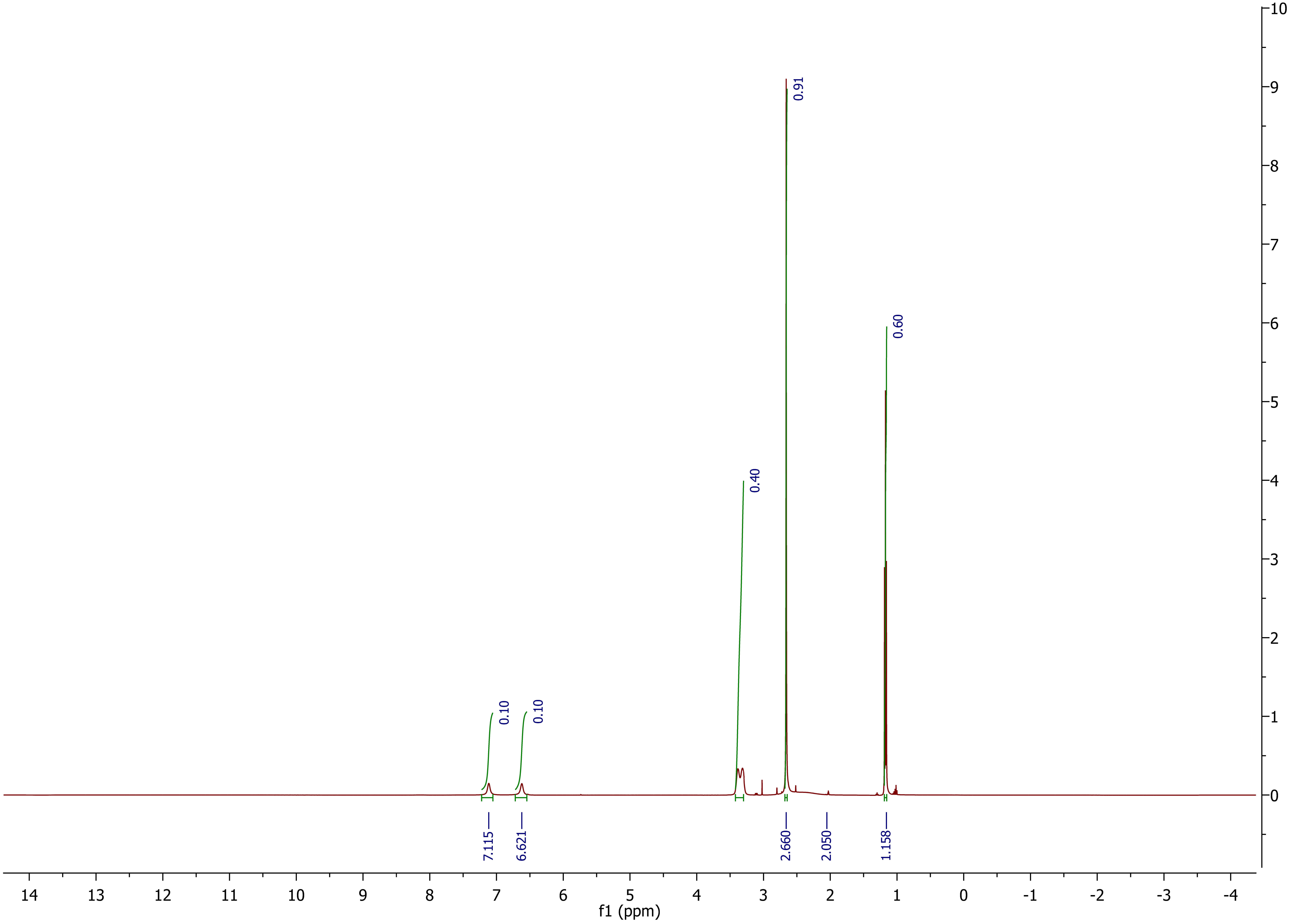

⑦

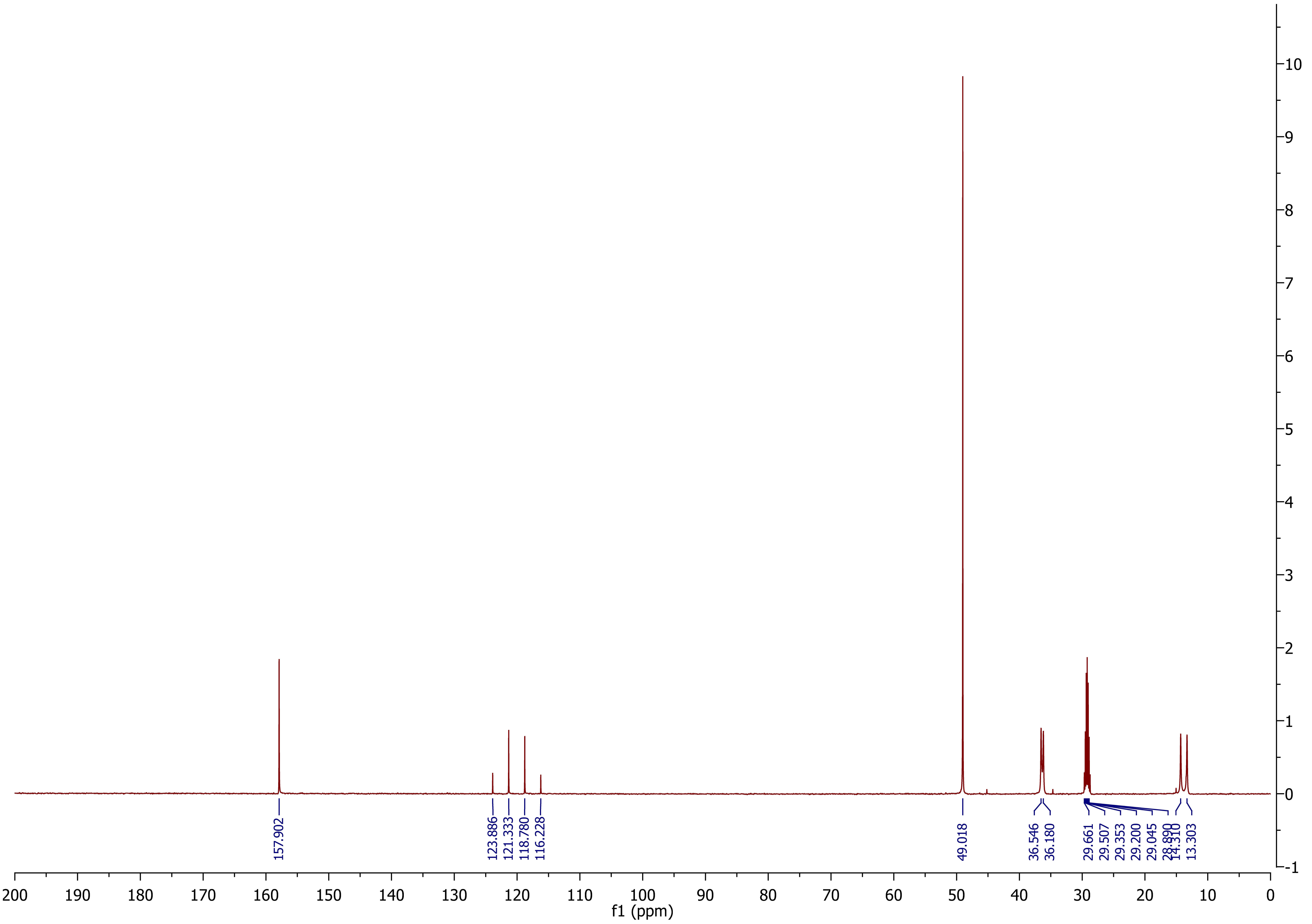
157.902
123.886
121.333
118.780
116.228
49.018
36.546
36.180
29.661
29.507
29.353
29.200
29.045
13.303
10
9
8
7
6
5
4
3
2
1
0
-1
200
190
180
170
160
150
140
130
120
110
100
90
80
70
60
50
40
30
20
10
0
f1 (ppm)

⑦

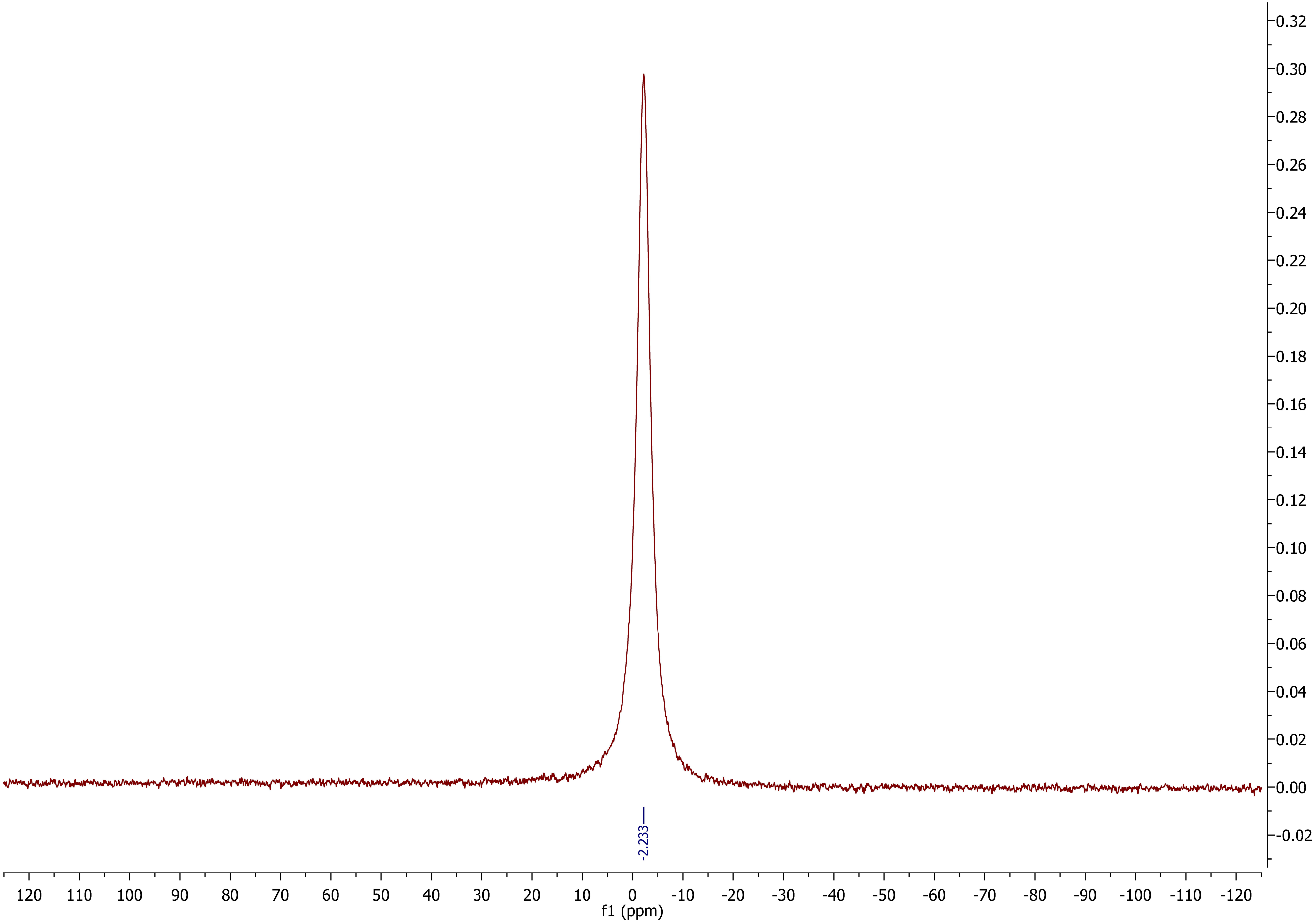

-2.233
f1 (ppm)
120
110
100
90
80
70
60
50
40
30
20
10
0
-10
-20
-30
-40
-50
-60
-70
-80
-90
-100
-110
-120
0.32
0.30
0.28
0.26
0.24
0.22
0.20
0.18
0.16
0.14
0.12
0.10
0.08
0.06
0.04
0.02
0.00
-0.02

⑦

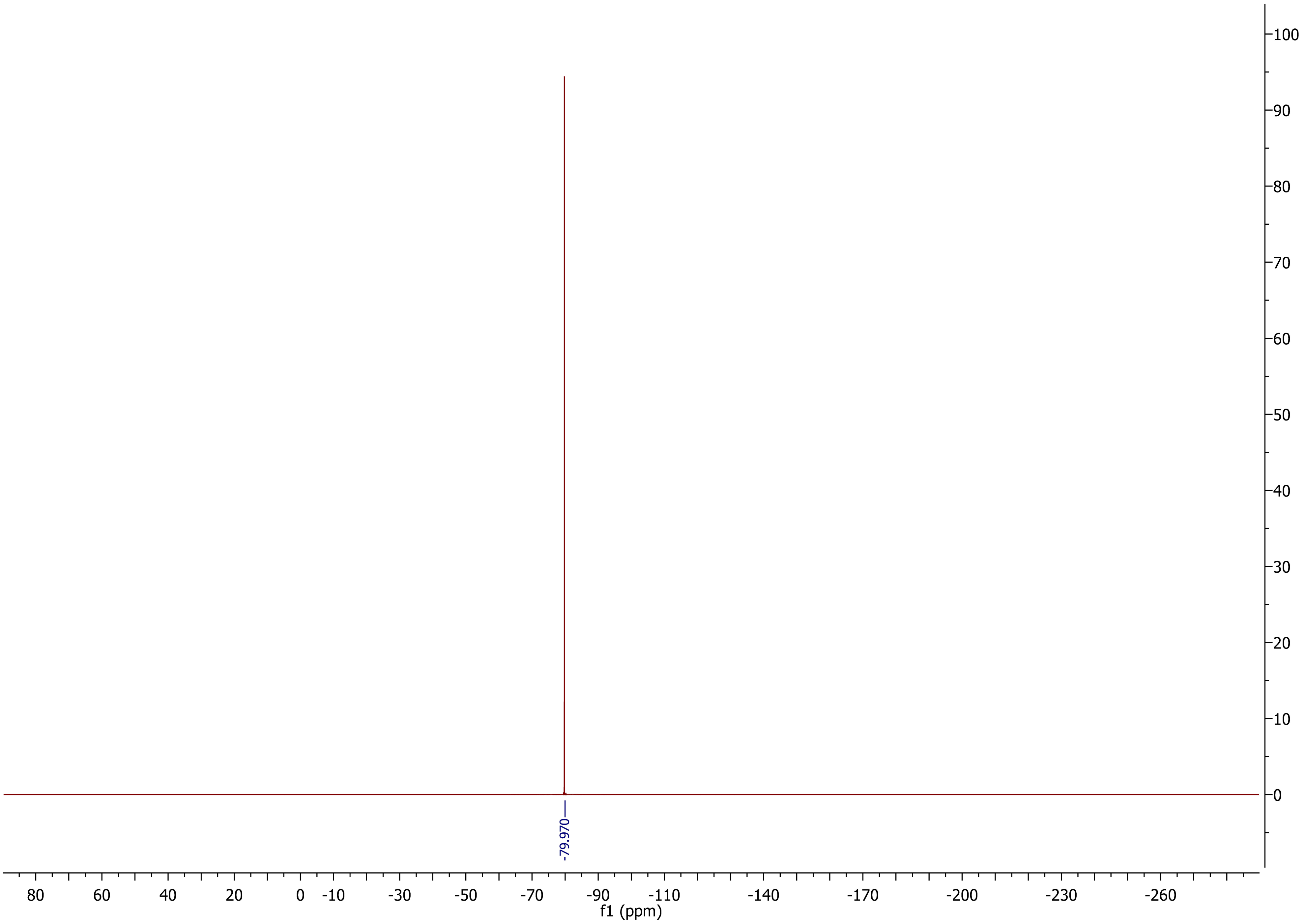
-79.970
100
90
80
70
60
50
40
30
20
10
0
80 60 40 20 0 -10 -30 -50 -70 -90 -110 -140 -170 -200 -230 -260
f1 (ppm)

⑧

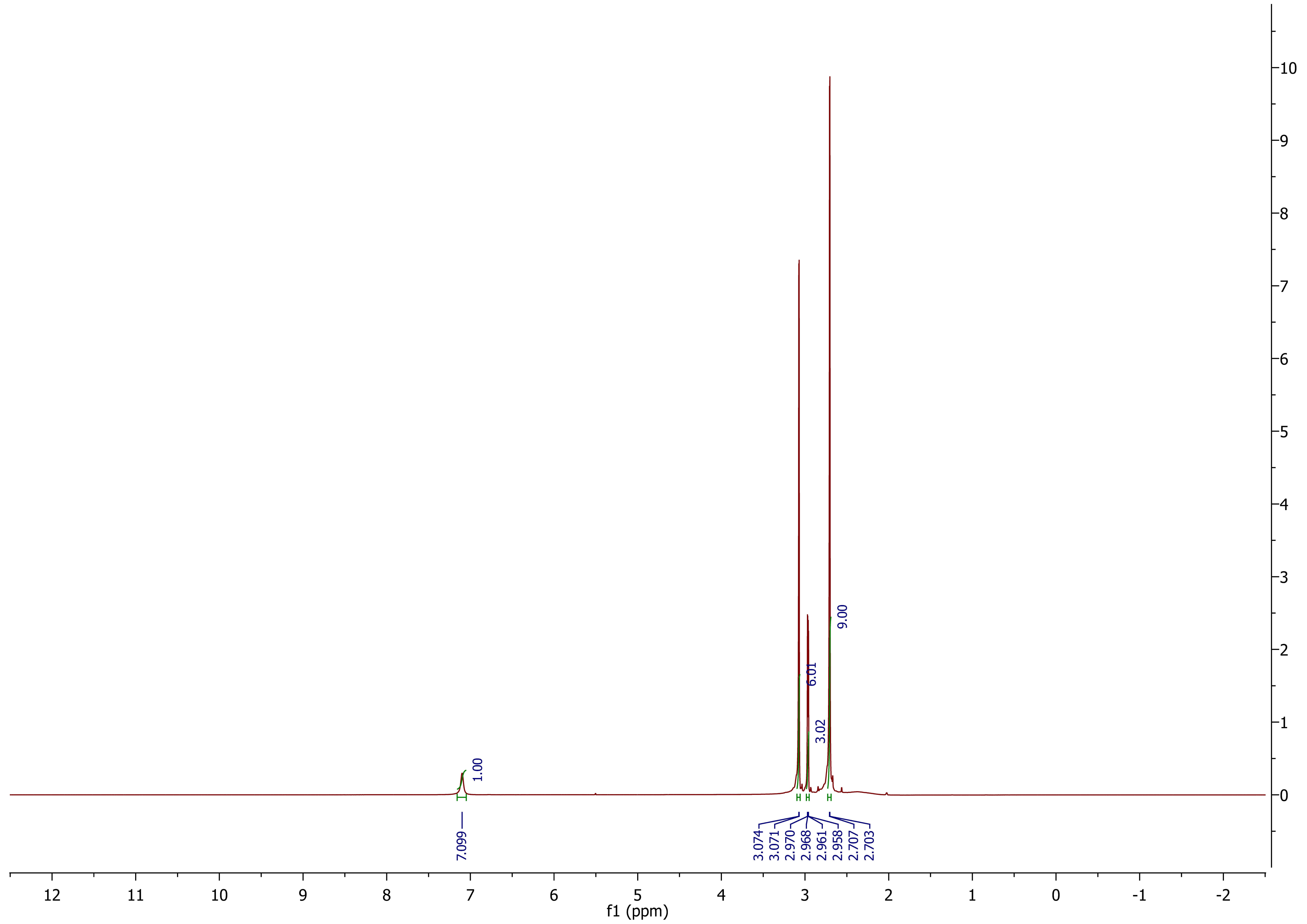

1.00
6.01
3.02
9.00
7.099
3.074
3.071
2.970
2.968
2.961
2.958
2.707
2.703
f1 (ppm)

⑧

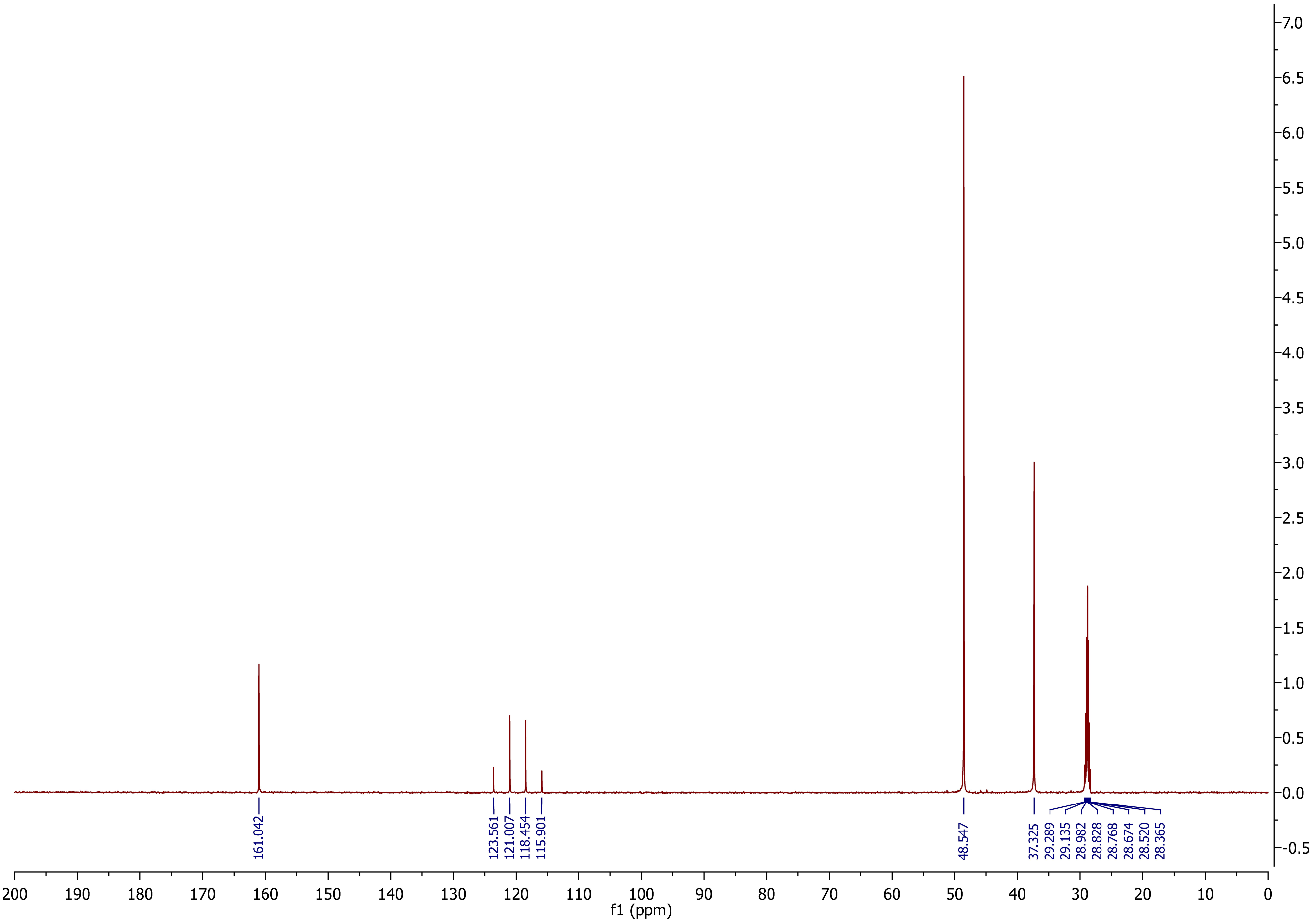

7.0
6.5
6.0
5.5
5.0
4.5
4.0
3.5
3.0
2.5
2.0
1.5
1.0
0.5
0.0
-0.5
161.042
123.561
121.007
118.454
115.901
48.547
37.325
29.289
29.135
28.982
28.828
28.768
28.674
28.520
28.365
200
190
180
170
160
150
140
130
120
110
100
90
80
70
60
50
40
30
20
10
0
f1 (ppm)

⑧

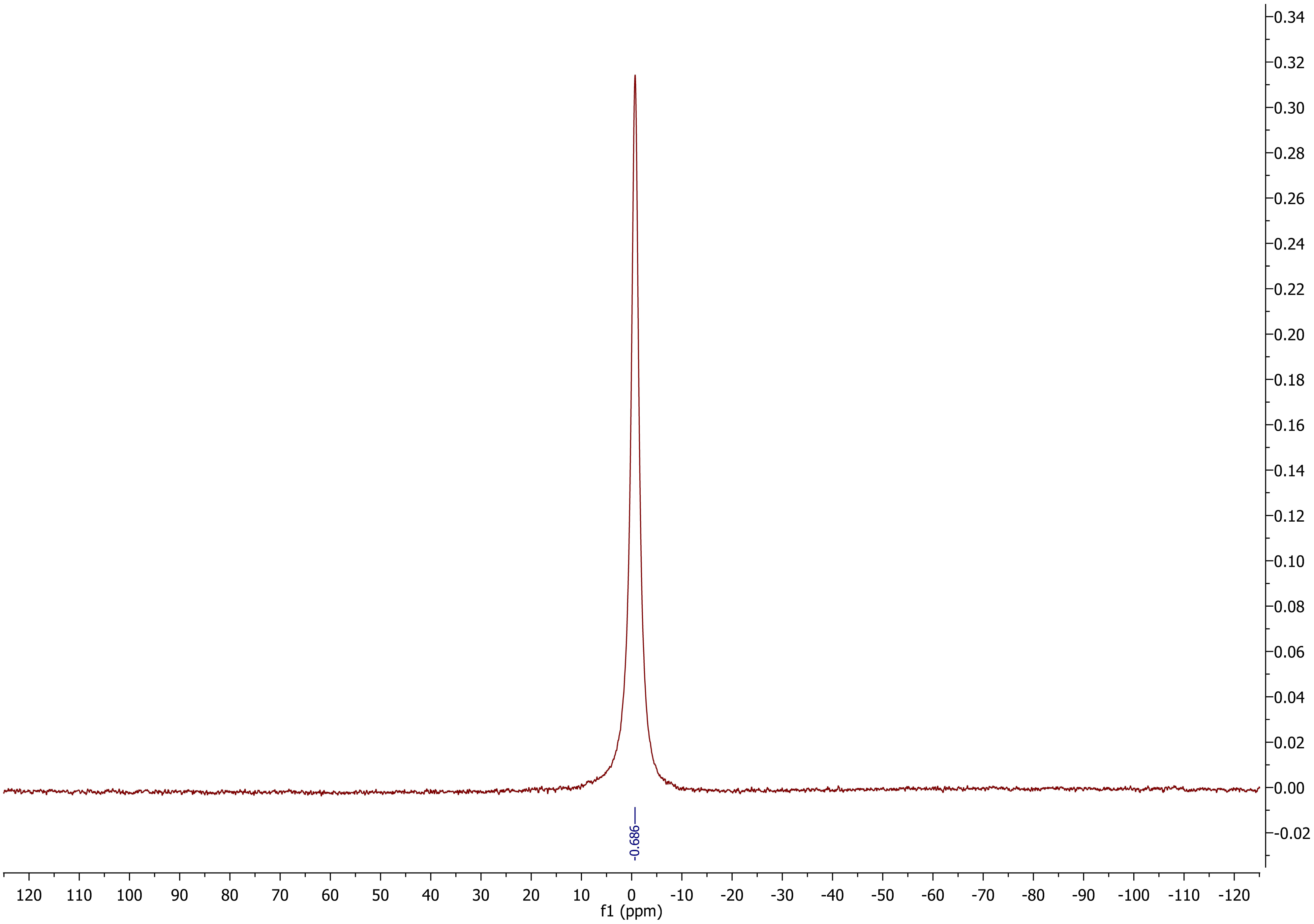
-0.686
f1 (ppm)
120
110
100
90
80
70
60
50
40
30
20
10
0
-10
-20
-30
-40
-50
-60
-70
-80
-90
-100
-110
-120
0.34
0.32
0.30
0.28
0.26
0.24
0.22
0.20
0.18
0.16
0.14
0.12
0.10
0.08
0.06
0.04
0.02
0.00
-0.02

⑧

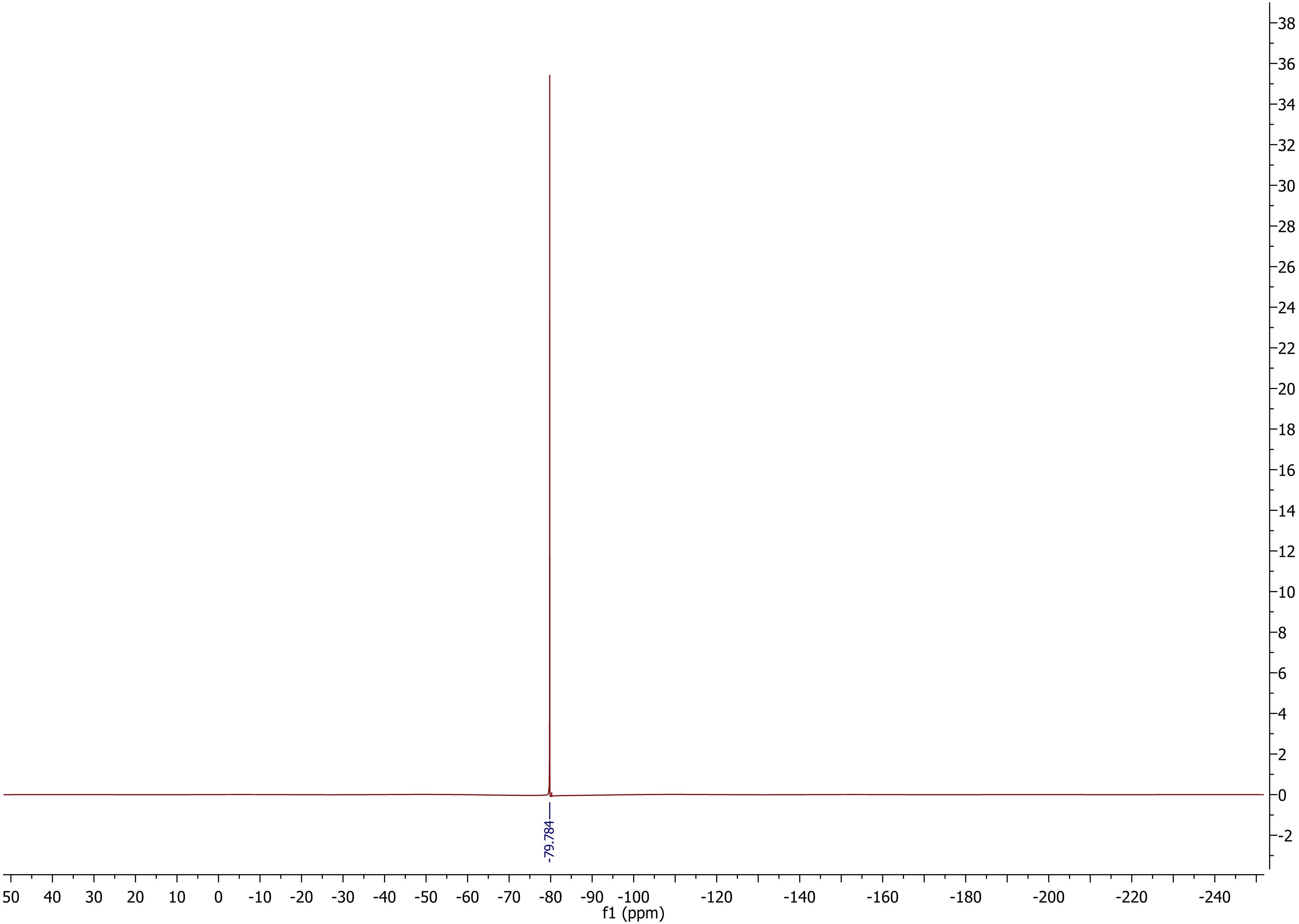
-79.784
50 40 30 20 10 0 -10 -20 -30 -40 -50 -60 -70 -80 -90 -100 -120 -140 -160 -180 -200 -220 -240
f1 (ppm)
38 36 34 32 30 28 26 24 22 20 18 16 14 12 10 8 6 4 2 0 -2

⑨

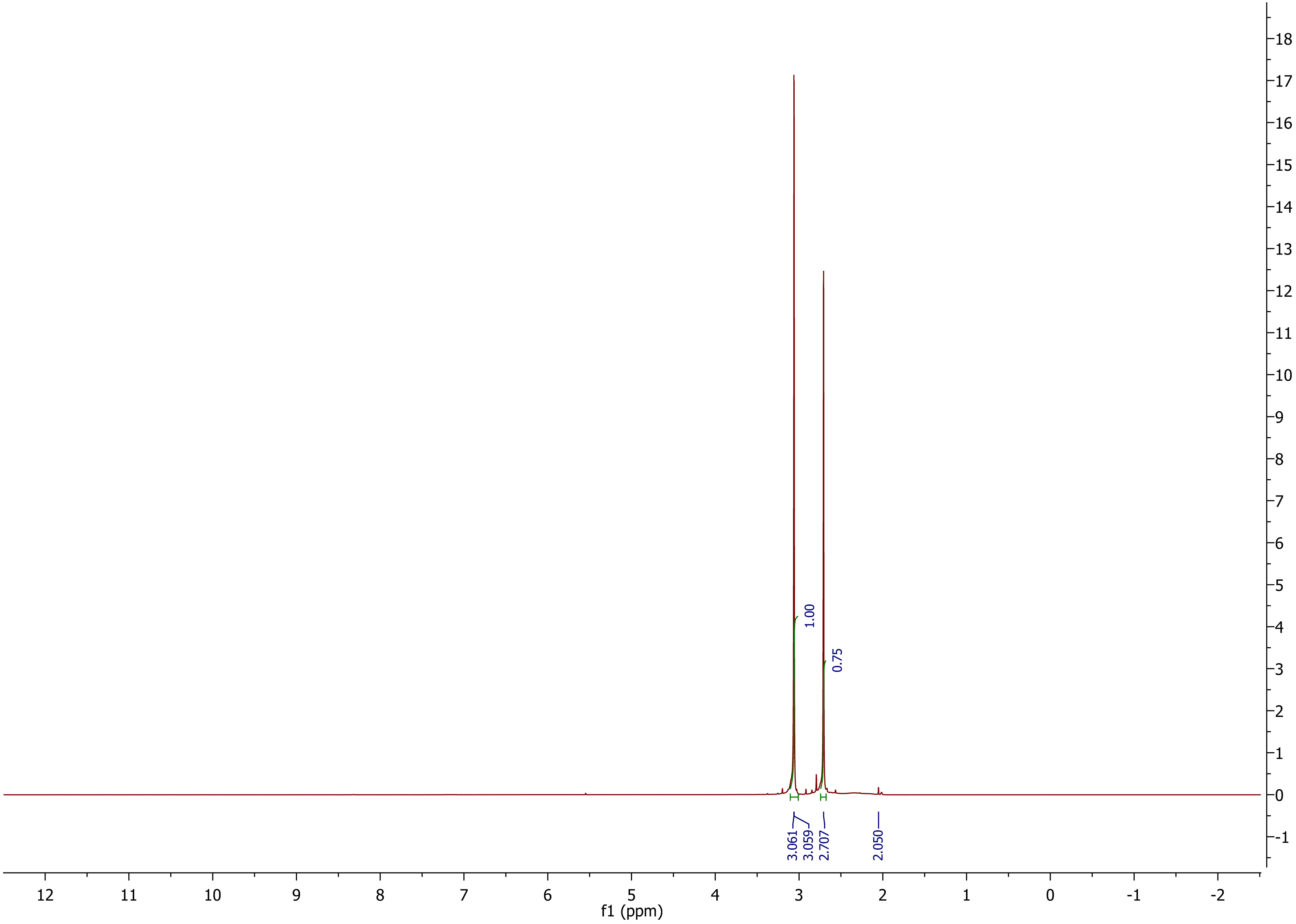

⑨

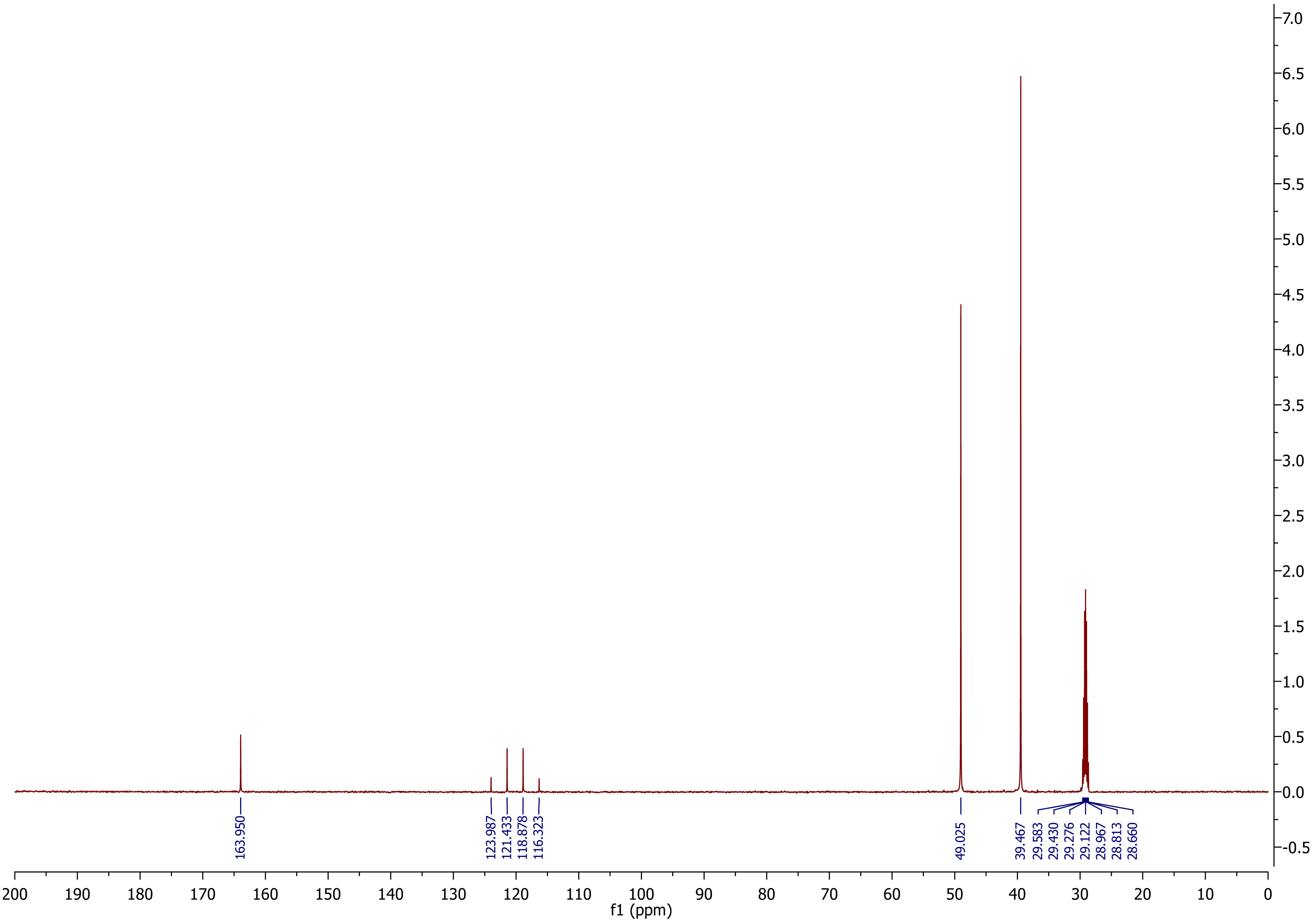
163.950
123.987
121.433
118.878
116.323
49.025
39.467
29.583
29.430
29.276
29.122
28.967
28.813
28.660
7.0
6.5
6.0
5.5
5.0
4.5
4.0
3.5
3.0
2.5
2.0
1.5
1.0
0.5
0.0
-0.5
200
190
180
170
160
150
140
130
120
110
100
90
80
70
60
50
40
30
20
10
0
f1 (ppm)

⑨

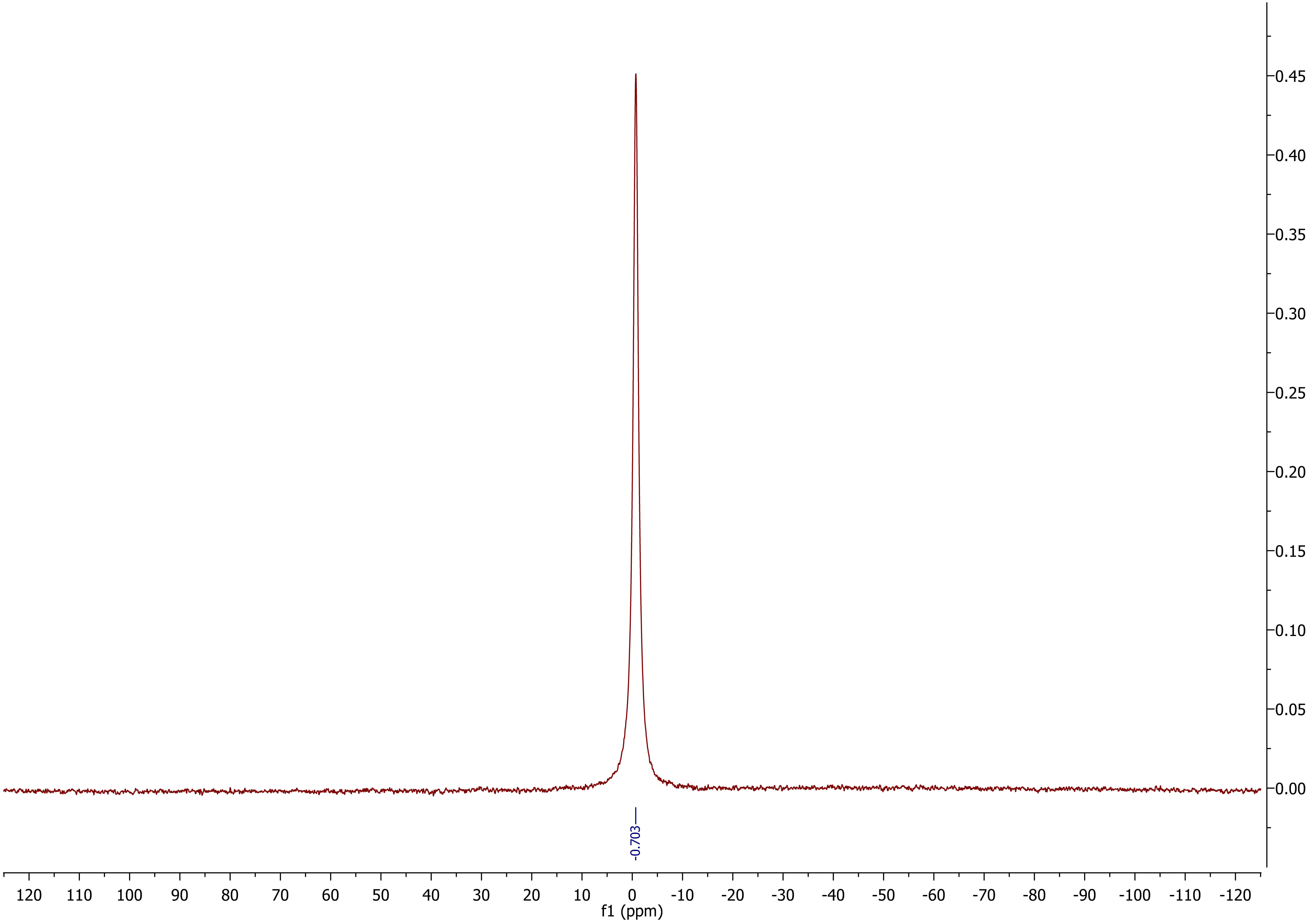
-0.703
0.45
0.40
0.35
0.30
0.25
0.20
0.15
0.10
0.05
0.00
120 110 100 90 80 70 60 50 40 30 20 10 0 -10 -20 -30 -40 -50 -60 -70 -80 -90 -100 -110 -120
f1 (ppm)

⑨

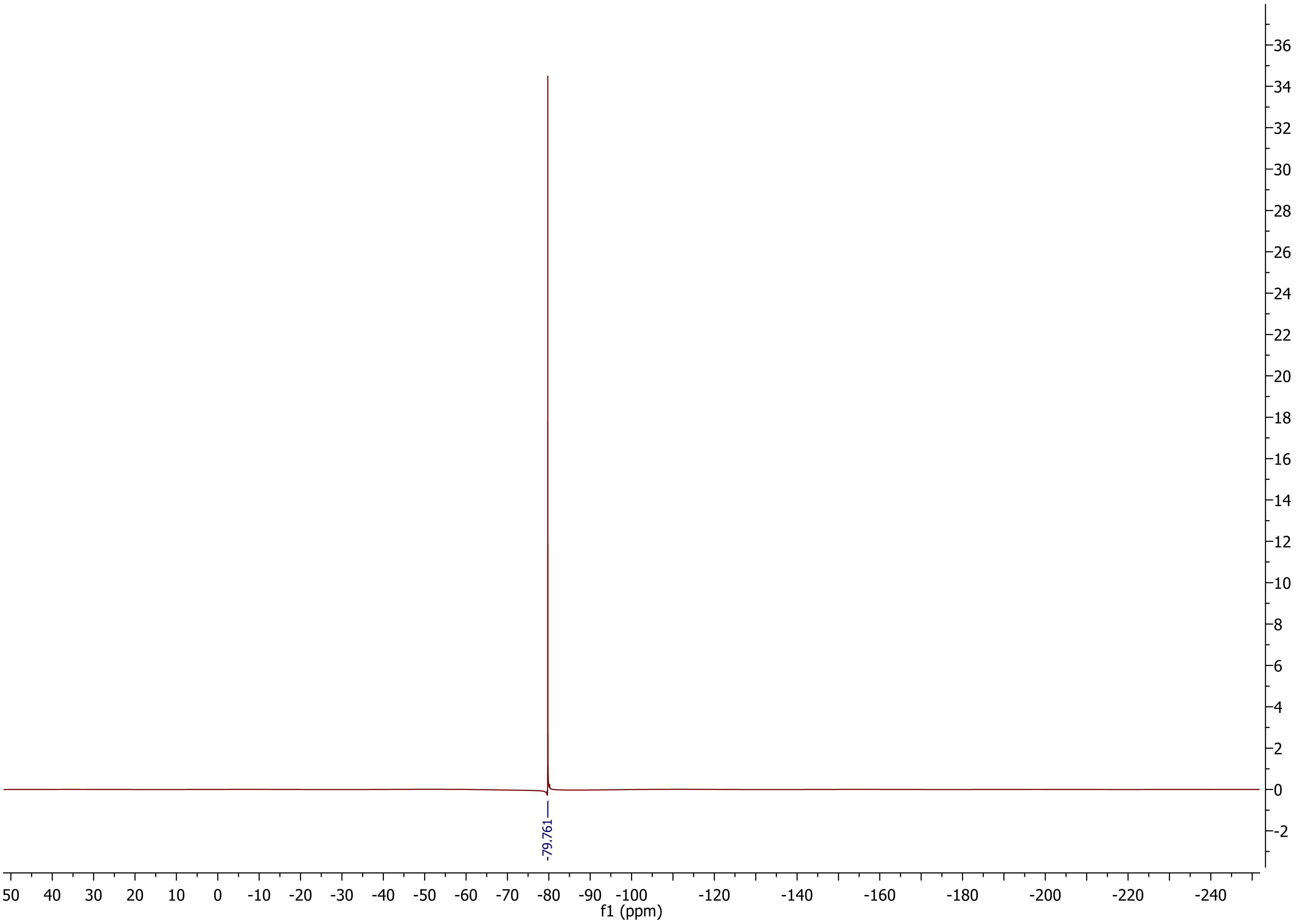
-79.761
50 40 30 20 10 0 -10 -20 -30 -40 -50 -60 -70 -80 -90 -100 -120 -140 -160 -180 -200 -220 -240
f1 (ppm)
36 34 32 30 28 26 24 22 20 18 16 14 12 10 8 6 4 2 0 -2

⑩

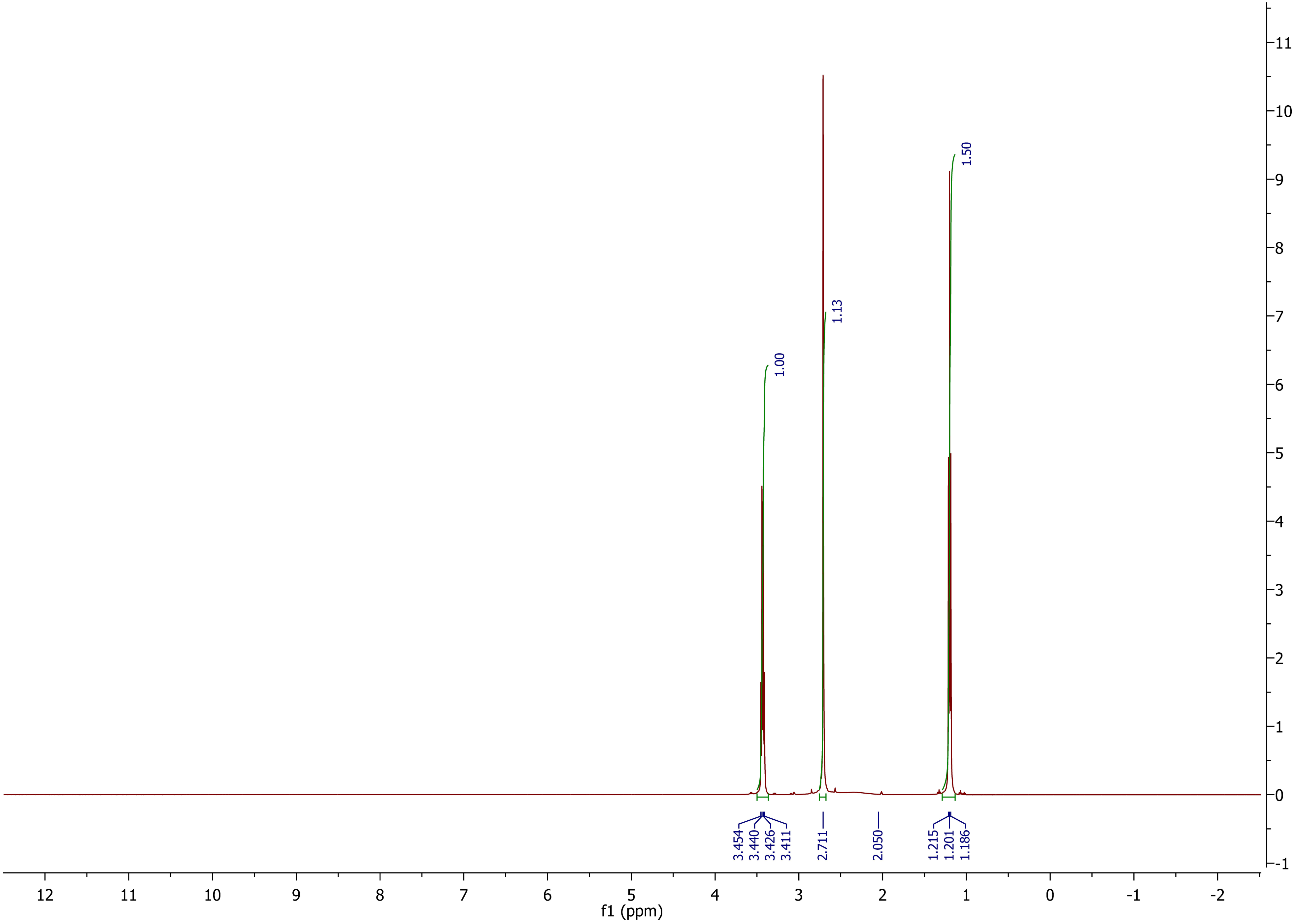
1.00
1.13
1.50
3.454
3.440
3.426
3.411
2.711
2.050
1.215
1.201
1.186
f1 (ppm)

⑩

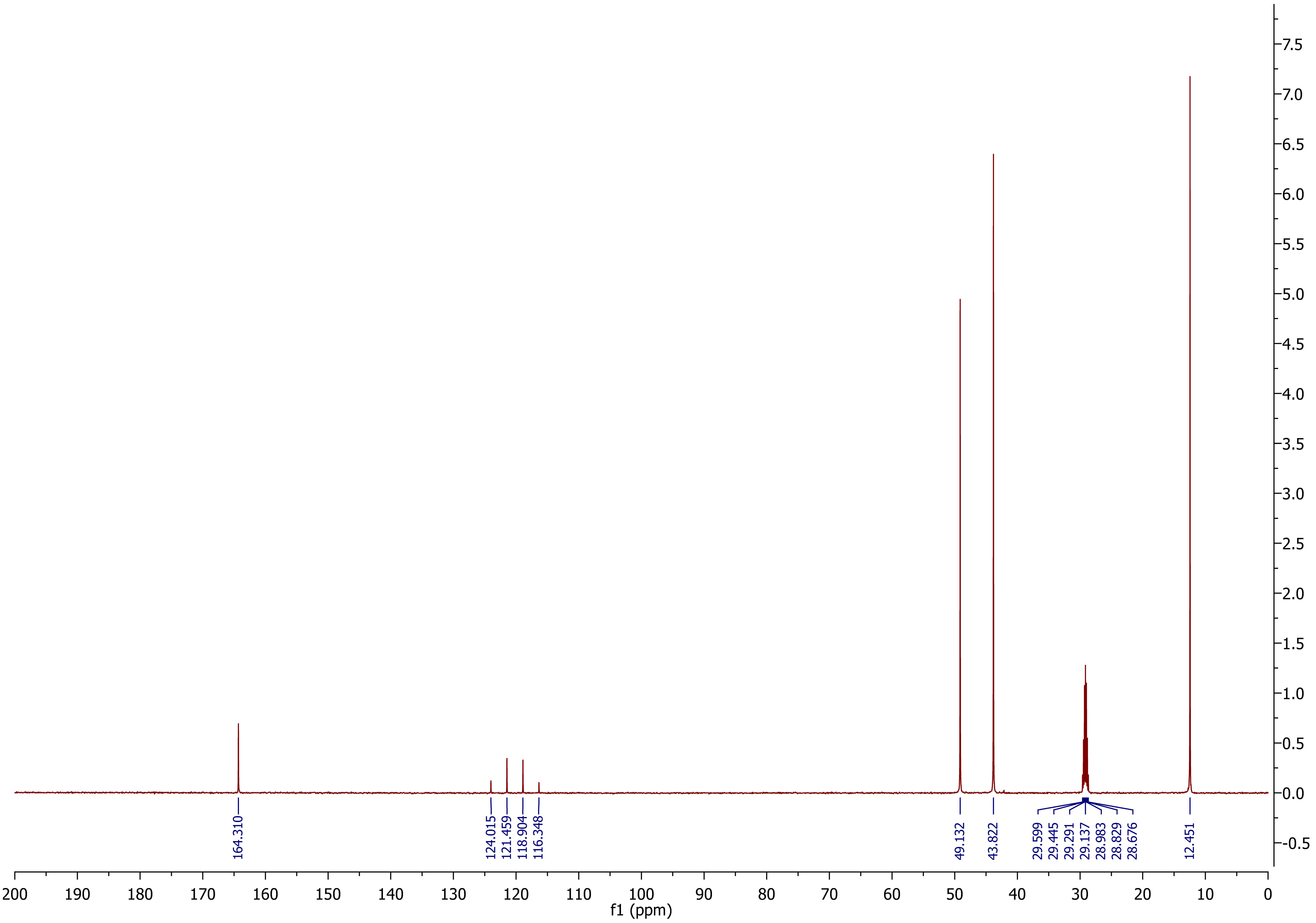
164.310
124.015
121.459
118.904
116.348
49.132
43.822
29.599
29.445
29.291
29.137
28.983
28.829
28.676
12.451
7.5
7.0
6.5
6.0
5.5
5.0
4.5
4.0
3.5
3.0
2.5
2.0
1.5
1.0
0.5
0.0
-0.5
200
190
180
170
160
150
140
130
120
110
100
90
80
70
60
50
40
30
20
10
0
f1 (ppm)

⑩

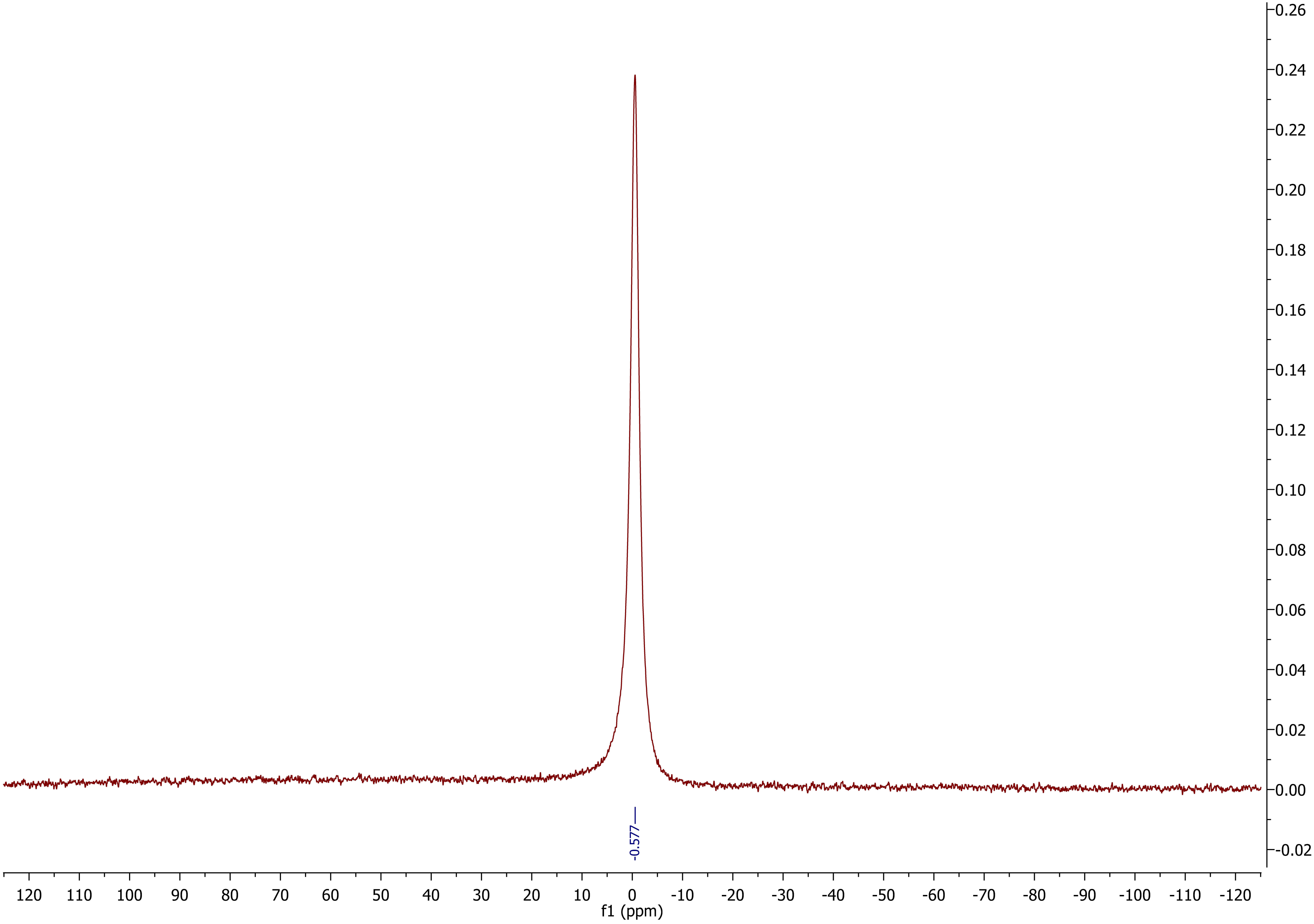
-0.577
0.26
0.24
0.22
0.20
0.18
0.16
0.14
0.12
0.10
0.08
0.06
0.04
0.02
0.00
-0.02
120 110 100 90 80 70 60 50 40 30 20 10 0 -10 -20 -30 -40 -50 -60 -70 -80 -90 -100 -110 -120
f1 (ppm)

⑩

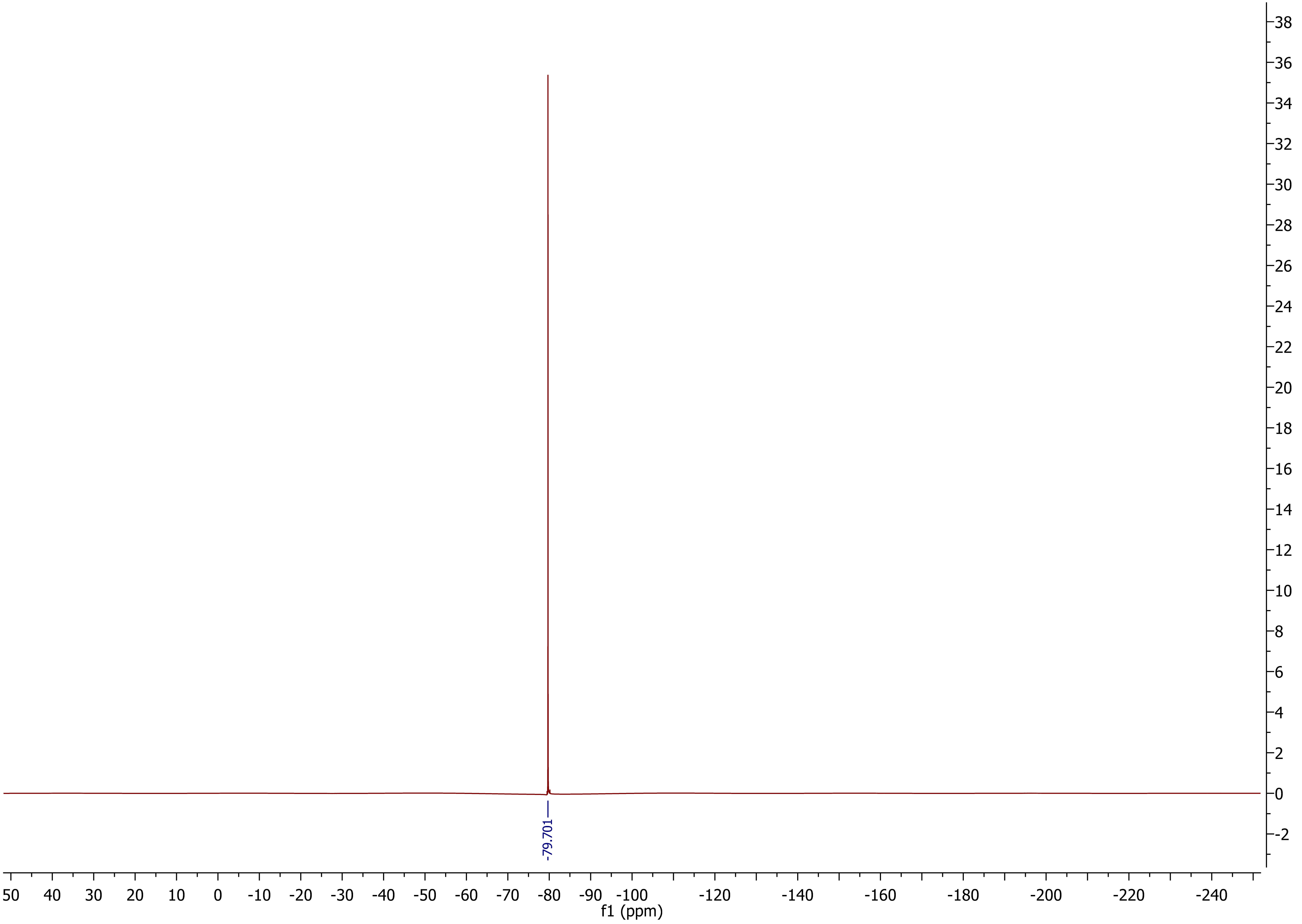

-79.701
50 40 30 20 10 0 -10 -20 -30 -40 -50 -60 -70 -80 -90 -100 -120 -140 -160 -180 -200 -220 -240
f1 (ppm)
38 36 34 32 30 28 26 24 22 20 18 16 14 12 10 8 6 4 2 0 -2

⑪

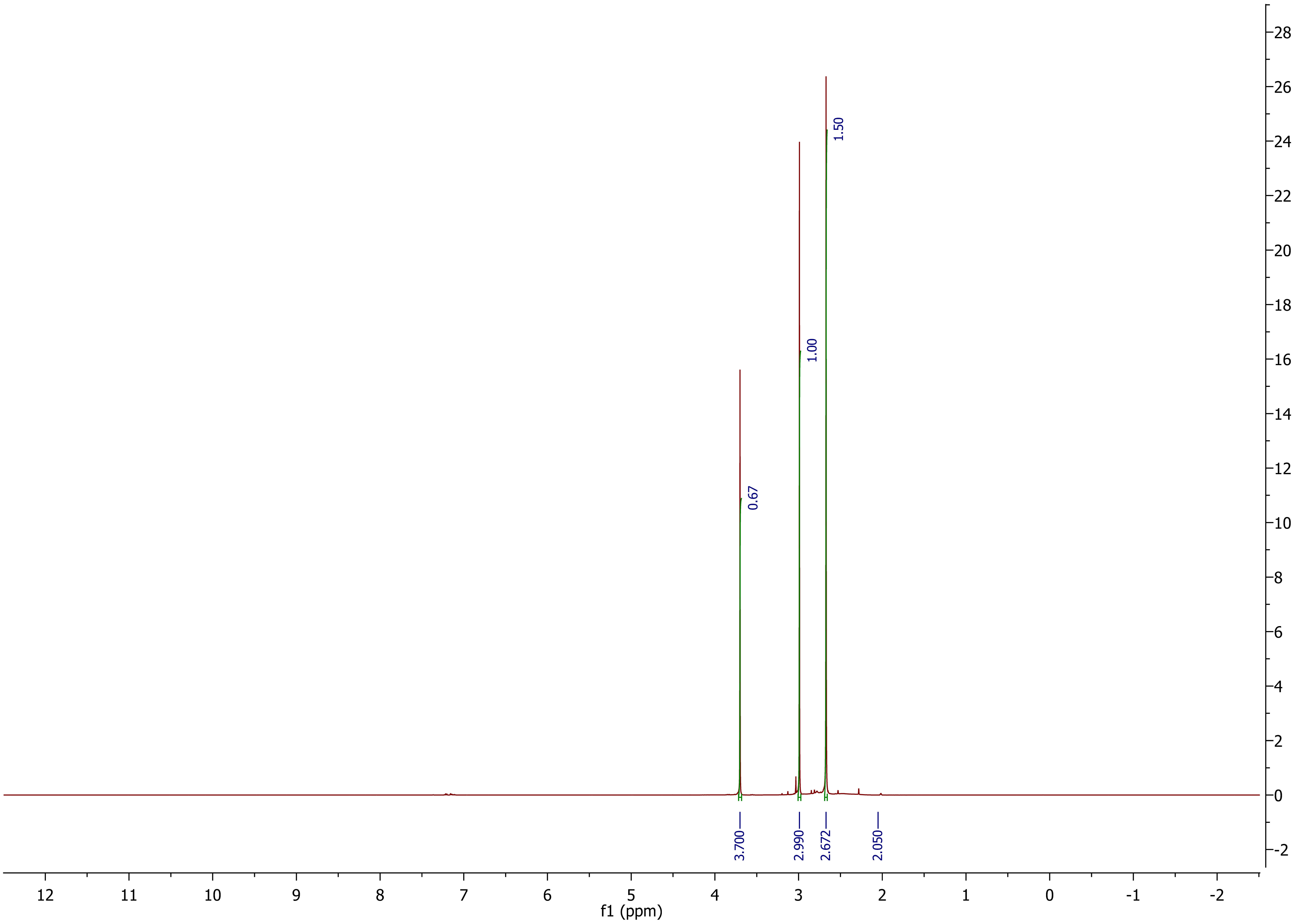

⑪

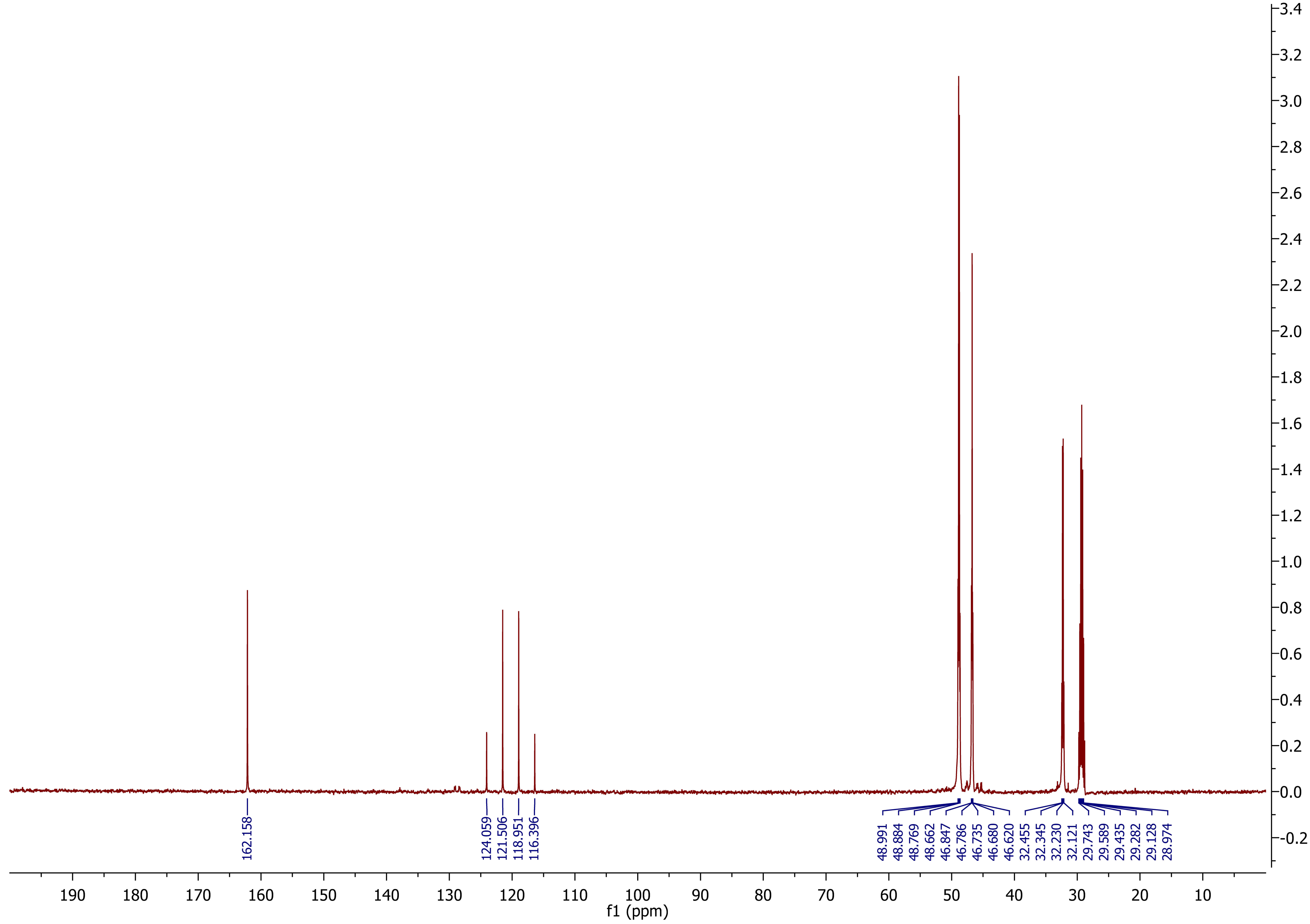
162.158
124.059
121.506
118.951
116.396
48.991
48.884
48.769
48.662
46.847
46.786
46.735
46.680
46.620
32.455
32.345
32.230
32.121
29.743
29.589
29.435
29.282
29.128
28.974
f1 (ppm)

⑪

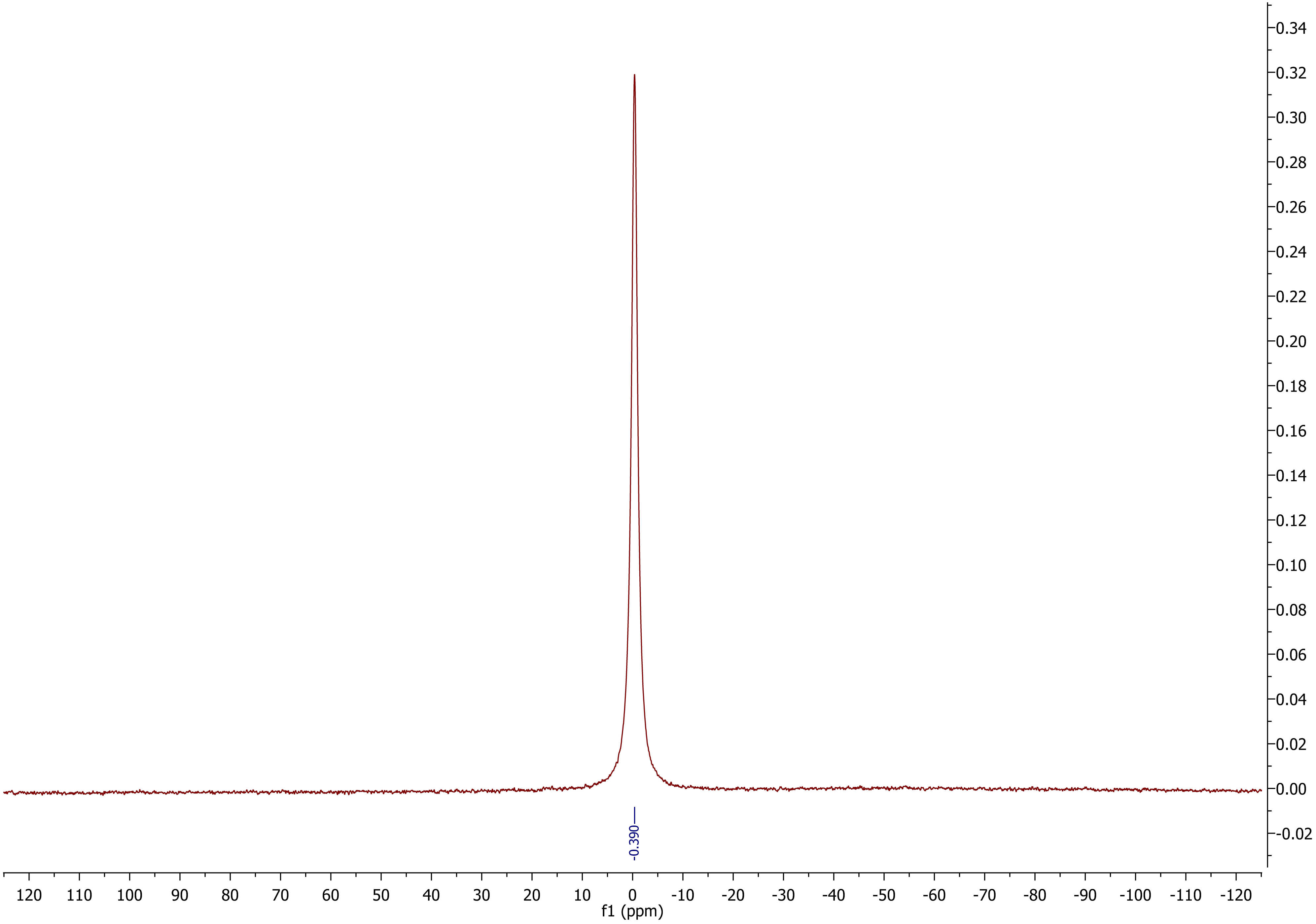

0.34
0.32
0.30
0.28
0.26
0.24
0.22
0.20
0.18
0.16
0.14
0.12
0.10
0.08
0.06
0.04
0.02
0.00
-0.02
-0.390
120 110 100 90 80 70 60 50 40 30 20 10 0 -10 -20 -30 -40 -50 -60 -70 -80 -90 -100 -110 -120
f1 (ppm)

⑪

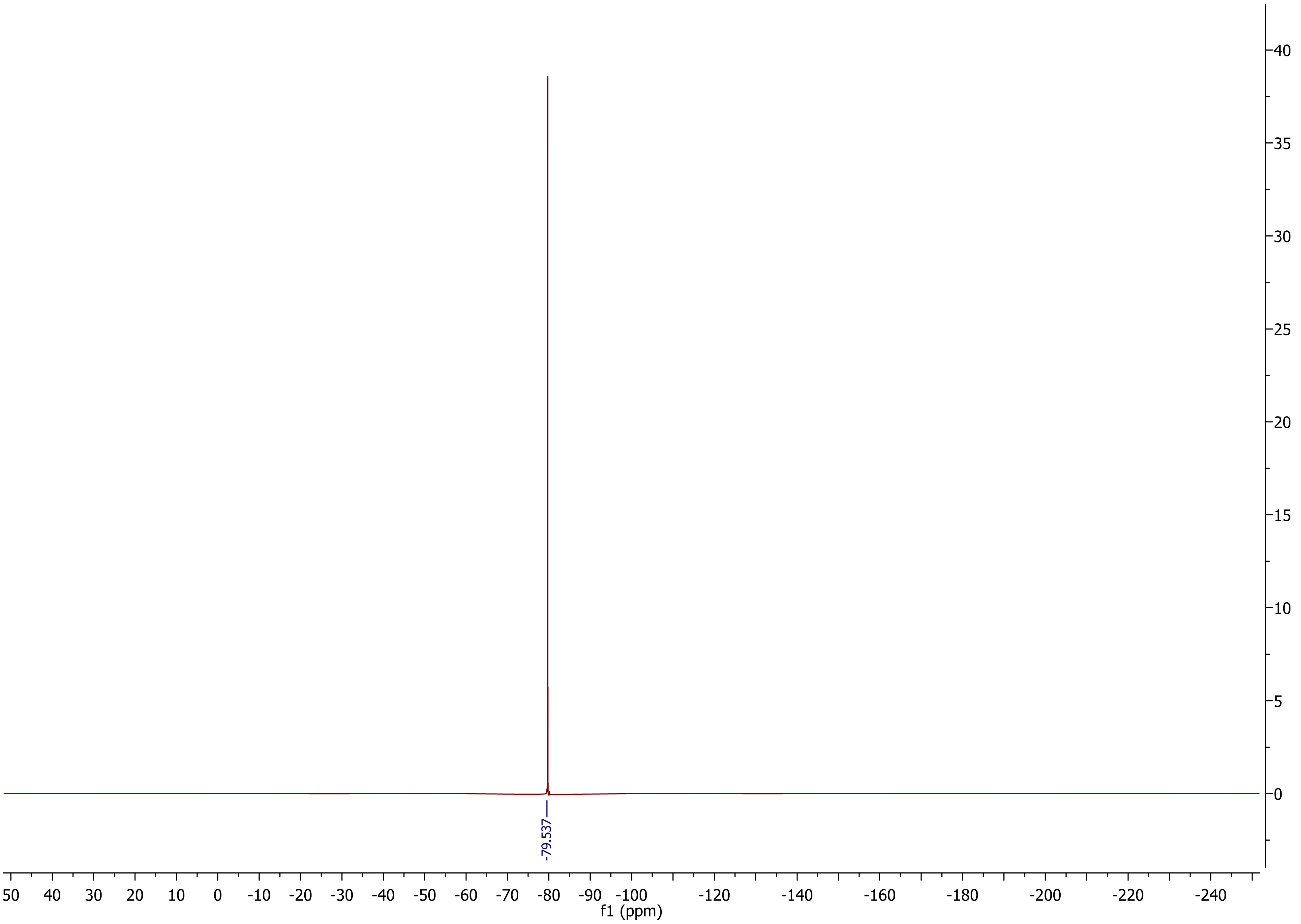
-79.537
40
35
30
25
20
15
10
5
0
50 40 30 20 10 0 -10 -20 -30 -40 -50 -60 -70 -80 -90 -100 -120 -140 -160 -180 -200 -220 -240
f1 (ppm)

⑫

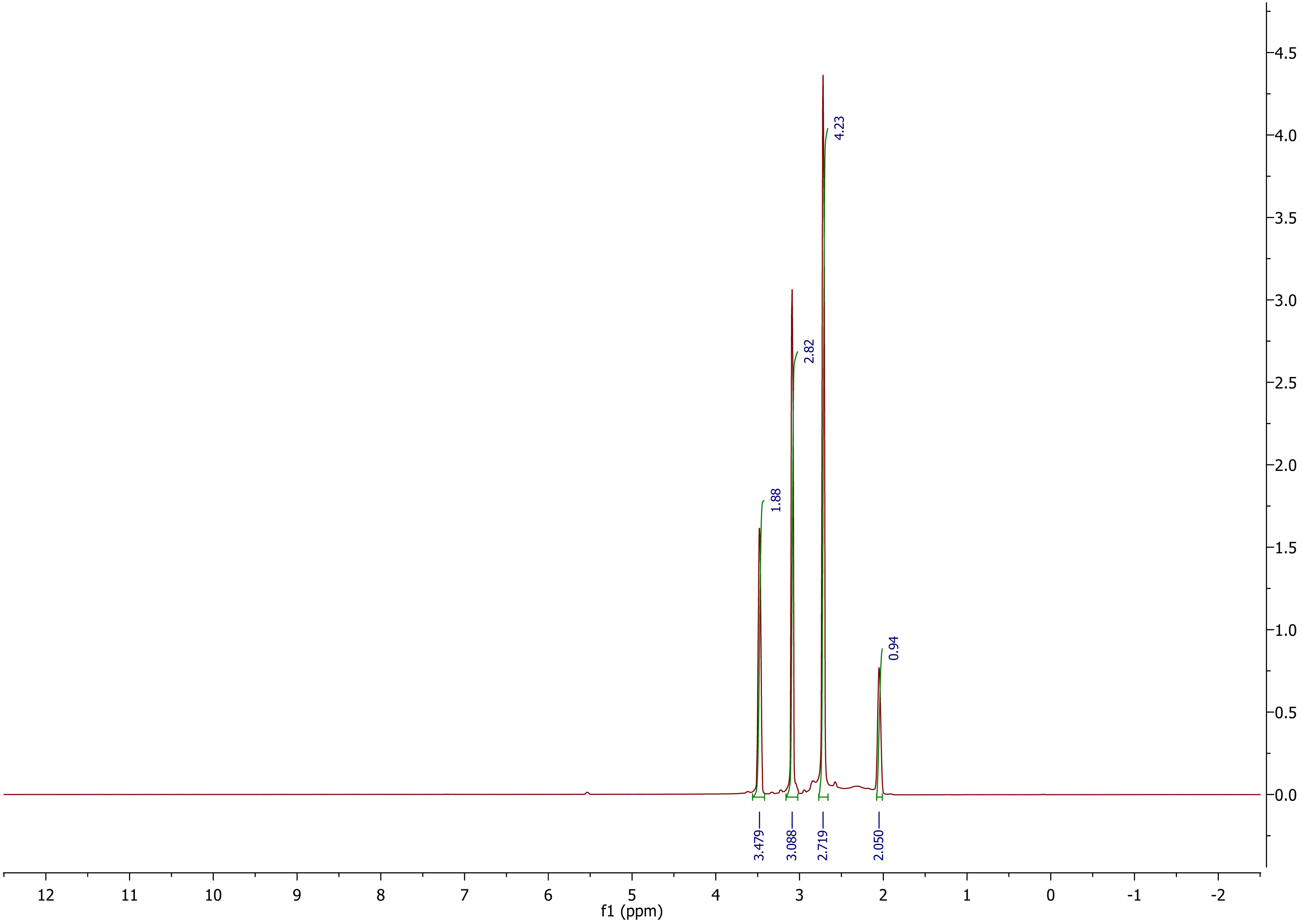

⑫

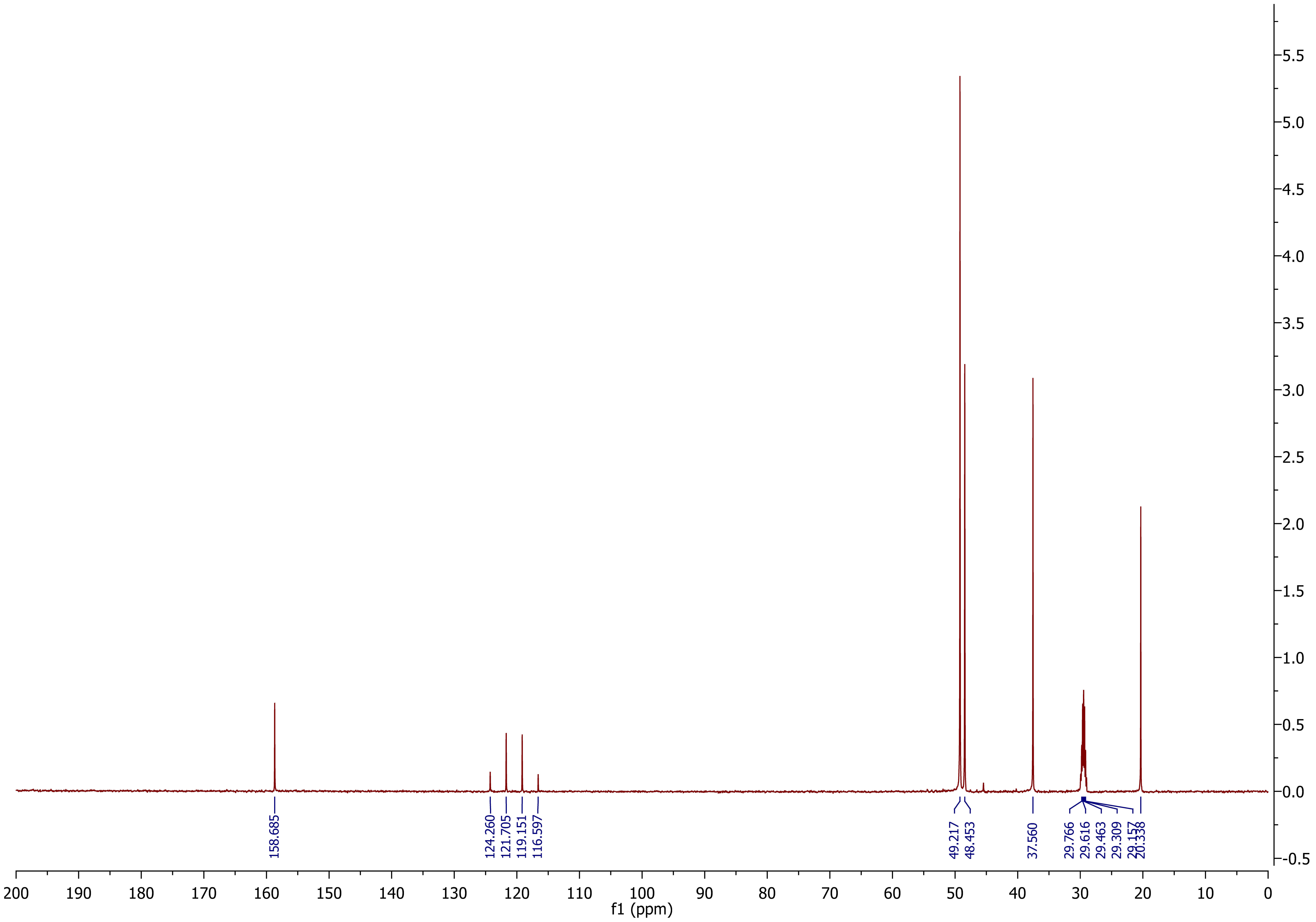
158.685
124.260
121.705
119.151
116.597
49.217
48.453
37.560
29.766
29.616
29.463
29.309
29.157
20.338
5.5
5.0
4.5
4.0
3.5
3.0
2.5
2.0
1.5
1.0
0.5
0.0
-0.5
200
190
180
170
160
150
140
130
120
110
100
90
80
70
60
50
40
30
20
10
0
f1 (ppm)

⑫

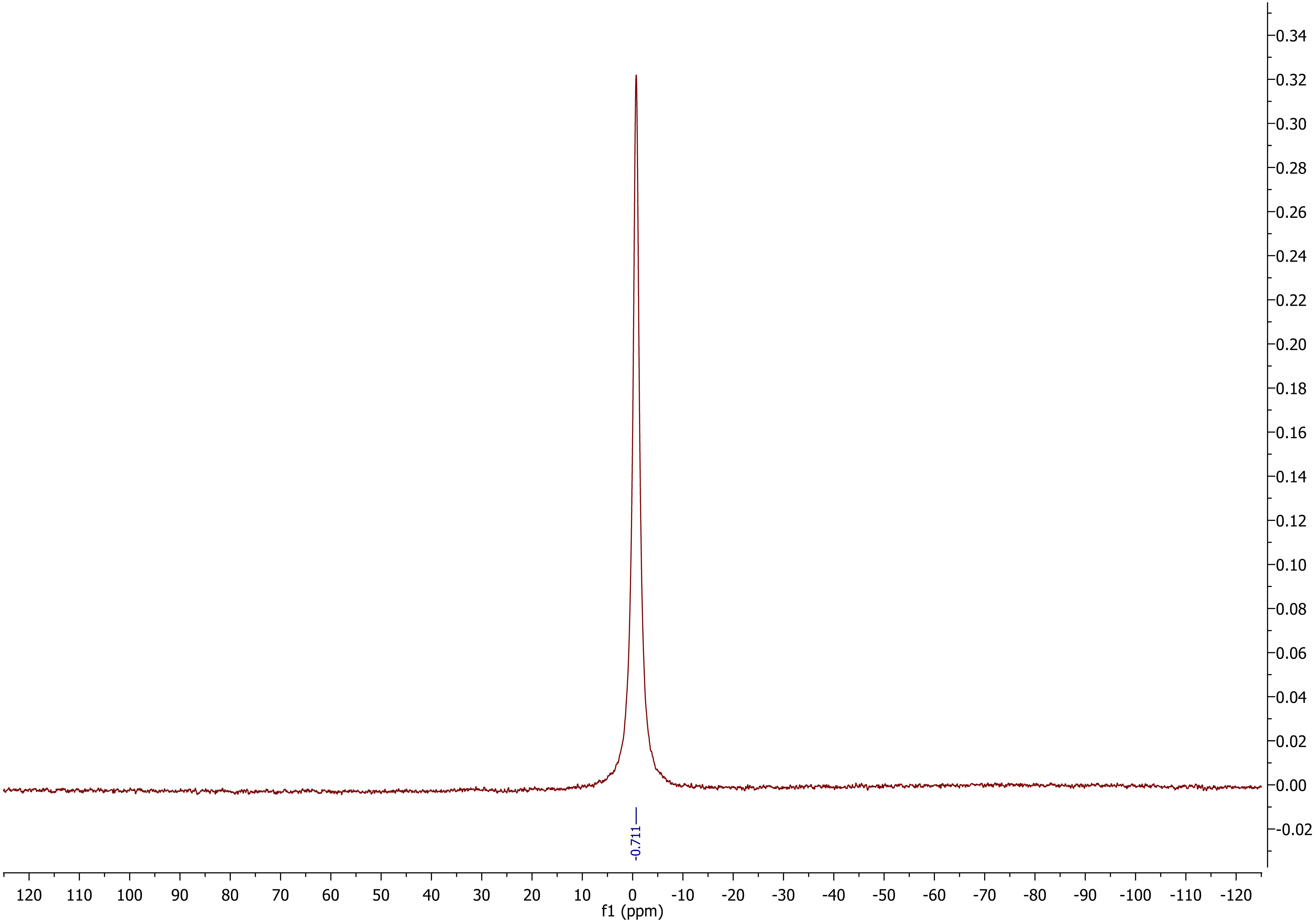

-0.711
0.34
0.32
0.30
0.28
0.26
0.24
0.22
0.20
0.18
0.16
0.14
0.12
0.10
0.08
0.06
0.04
0.02
0.00
-0.02
120 110 100 90 80 70 60 50 40 30 20 10 0 -10 -20 -30 -40 -50 -60 -70 -80 -90 -100 -110 -120
f1 (ppm)

⑫

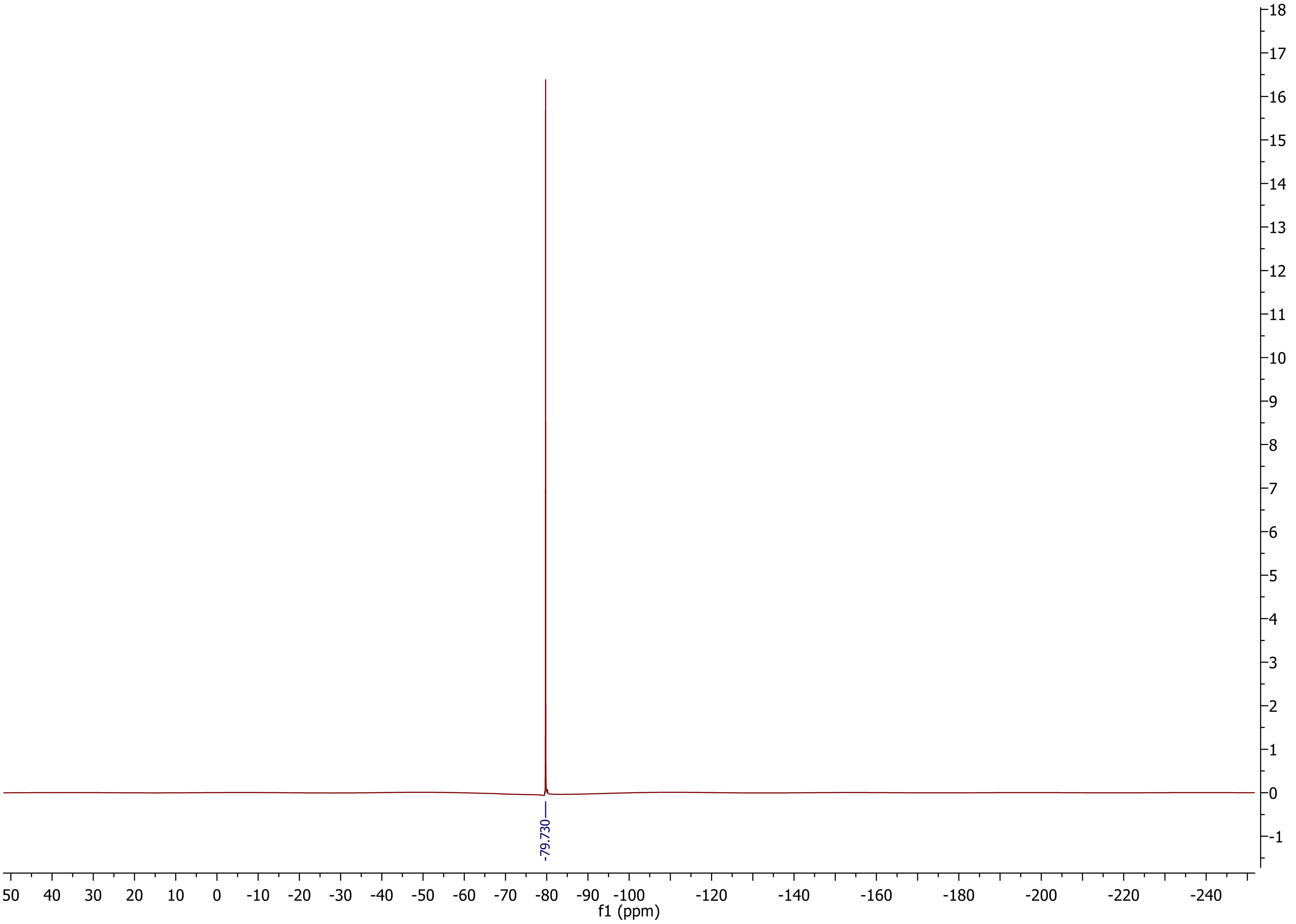

Supplement: RA-015-D5RA05311K-s001 [file RA-015-D5RA05311K-s001.pdf]
